# Integrating Bulk and Single-cell RNA-seq to Construct a Macrophage-related Prognostic Model for Prognostic Stratification in Triple-negative Breast Cancer

**DOI:** 10.7150/jca.101042

**Published:** 2024-09-23

**Authors:** Hongmeng Zhao, Xuejie Zhou, Guixin Wang, Yue Yu, Yingxi Li, Zhaohui Chen, Wenbin Song, Liwei Zhao, Li Wang, Xin Wang, Xuchen Cao, Yao Tian

**Affiliations:** 1 The First Department of Breast Cancer, Tianjin Medical University Cancer Institute and Hospital, National Clinical Research Center for Cancer, Tianjin 300060, China.; 2 Key Laboratory of Cancer Prevention and Therapy, Tianjin 300060, China.; 3 Tianjin's Clinical Research Center for Cancer, Tianjin 300060, China.; 4 Key Laboratory of Breast Cancer Prevention and Therapy, Tianjin Medical University, Ministry of Education, Tianjin 300060, China.; 5 Key Laboratory of Immune Microenvironment and Disease (Ministry of Education), Tianjin Medical University, Tianjin 30007, China.; 6 Department of General Surgery, Tianjin Medical University General Hospital, Tianjin 300052, China.

**Keywords:** single-cell RNA-seq, triple-negative breast cancer, macrophage, prognostic model, individual treatment

## Abstract

**Background:** Triple-negative breast cancer (TNBC) is a poor prognostic subtype of breast cancer due to limited treatment. Macrophage plays a critical role in tumor growth and survival. Our study intends to explore the heterogeneity of macrophage in TNBC and establish a macrophage-related prognostic model for TNBC prognostic stratification.

**Materials and Methods:** Seurat package was conducted to analyze the single-cell RNA expression profilers. The cell types were identified by the markers derived from public research and online database. The cell-cell interactions were calculated by the CellChat package. Monocle package was used to visualize the cell trajectory of macrophages. The prognostic model was constructed by six macrophage-related genes after a series of selections. The expression of six genes were validated in normal and TNBC tissues. And several potential agents for high-risk TNBC patients were analyzed by Connectivity Map analysis.

**Results:** Nine cell types were identified, and the macrophages were highly enriched in TNBC samples. five distinct subgroups of macrophage were identified. Notably, SPP1+ tumor-associated macrophages exhibited a poor prognosis. The prognostic model was constructed by *HSPA6*, *LPL*, *IDO1*, *ALDH2*, *TK1*, and *QPCT* with good predictive accuracy at 3-, 5- years overall survival for TNBC patients in both training and external test cohorts. Finally, several drugs were identified for the high-risk TNBC patients decided by model.

**Conclusion:** Our study provides a valuable source for clarifying macrophage heterogeneity in TNBC, and a promising tool for prognostic risk stratification of TNBC.

## Introduction

Breast cancer (BRCA) has remained to be one of the most common malignancies in females worldwide in recent years^1^-^3^. Triple-negative breast cancer (TNBC) is considered to be the molecular subtype with the poorest prognosis in BRCA due to tumor heterogeneity and limited treatment^4^. The lack of effective treatments for TNBC other than chemotherapy results in shorter median survival times for TNBC patients compared to patients with other molecular subtypes^5^, ^6^. Therefore, it is imperative to accurately stratify the prognosis of TNBC patients and find novel therapeutic targets.

Tumor-associated macrophages (TAMs), as heterogeneous immune cells, have been confirmed to be critical for tumor progression^7^, ^8^. Tumor-associated macrophages (TAMs) generally include M2 and M1 macrophages, which play pro-tumor and anti-tumor roles during tumor progression, respectively^9^, ^10^. With the further exploration of TAM in recent years, the pro-tumor effect of TAM in the tumor microenvironment has been further revealed. TAMs are able to interact with stromal and stromal cells to form an microenvironment for tumor growth and metastasis^11^. Also, a large number of studies showed that TAMs could play a suppressive role on natural killer cells and cytotoxic T cells^7^, leading to survival of tumor cells. Therefore, macrophage-related markers identification may help predict the prognosis and immune state for tumor patients. The maturation of single-cell RNA sequencing (scRNA-seq) technology has led to a better understanding of the subgroups of TAMs and the discovery of novel prognostic markers and therapeutic targets. Li *et al.*^12^ found *SPP1* (+) and *C1QC* (+) TAMs gene signatures could classify cervical patients into subgroups with different immune states, tumor stages, and prognoses. Yang *et al.*^13^ revealed that two distinct immune suppressive TAMs (*CCL18*+ macrophages and *SPP1*+ macrophages) existed in non-small lung cancer and high expression of *SPP1*, *FN1*, *C1QC* in immune cell was associated with poor prognosis in early-stage non-small lung cancer. However, few studies have focused on the TAM subgroup in TNBC to predict patient outcomes through scRNA-seq technology. Although a previous study has analyzed the tumor-associated macrophages in TNBC, it only focused on the prognostic value of M2-macrophages^14^. More single-cell samples and bulk samples should be included to elucidate the functional and prognostic roles of TAMs in TNBC.

Herein, we comprehensively analyzed the role of TAMs in TNBC by integrating single cell and bulk RNA datasets. Five distinct subgroups of TAMs in TNBC tissues were identified and macrophages-related genes were obtained. Subsequently, the prognostic risk model we constructed which showed a great predictive effect for 3-, 5- years overall survival in TNBC patients. Finally, we identified several agents for the individualized treatment of high-risk patients. Our findings describe the heterogenicity of TAMs in TNBC, and provide a provide a theoretical basis for prognostic risk stratification and individualized treatment of TNBC.

## Materials and Methods

### Data collection

The TNBC single-cell RNA dataset was obtained from the GEO database (https://www.ncbi.nlm.nih.gov/geo/query/acc.cgi?acc=GSE199515). In detail, the 3 TNBC samples derived from human were included in our study. Additionally, the bulk RNA datasets were downloaded from cBioPortal database^15^ and TCGA database, including METABRIC and TCGA-BRCA datasets. The TCGA-BRCA dataset was downloaded by TCGAbiolinks package. The patients diagnosed with TNBC with prognostic information in both METABRIC dataset (319 samples) and TCGA-BRCA dataset (107 TNBC and 113 normal samples) were enrolled in this study.

### Data preprocessing

The high-quality cells were obtained by following procedures. (1) 200< nFeature_RNA; (2) 500 < nCount_RNA; (3) percentage_mt ≤15%; (4) log10FeaturePerUMI ≥ 0.8. DoubleFinder^16^ package was conducted to remove potential doublelet. Harmony package was utilized to remove the batch effect of different sample. glmGamPoi package was conducted to normalized the scRNA-seq expression data by SCTtransform method. The top 3000 highly variable genes were recognized for principal component analysis. The UMAP and t-SNE in Seurat were used to reduce the feature dimension and visualized the different cell clusters^17^. The cell markers used for cell identification were obtained from public database and research^18^, ^19^.

### CIBERSOFTx analysis

To infer the proportions of distinct cell types in each TNBC sample in TCGA dataset, we upload the single cell gene matrix and bulk RNA gene matrix on CIBERSOFTx website^20^. In detail, 100 cells of each type were randomly selected to make the single cell gene matrix. The Wilcoxon test was conducted to exam the statistical differences. P < 0.05 was considered as statistically significant.

### Cell-cell interaction analysis

CellChat package was used to infer the interaction number and strength among distinct cell types in scRNA-seq dataset^21^. And the ligand-receptor pairs between macrophage and other cell types were further analyzed. P-value < 0.05 was thought as statistically significant.

### Pseudotime analysis

Macrophages were extracted for re-clustered in five subgroups using SCTtransform method. Monocle was utilized to infer the pseudotime trajectory of macrophage. The DDRTree method was performed for dimensionality reduction. The max component was set as 2. The markers of tumor-associated macrophages were collected from the public research to annotate the macrophage types^22^. GSEA analysis was performed to analyze the biological function for macrophage subgroups. The group were divided into high and low group by the best cutoff of cell proportion using survminer package. Also, the Kaplan-Meier curve was performed to assess the prognostic outcome. Values with P < 0.05 were considered statistically significant.

### Differential expression and functional enrichment analyses

The differential-expression analysis was performed by edegR package^23^ to identified the significant genes between normal and TNBC samples. The 1482 differential expression genes (DEG) were obtained. The KEGG pathway and disease ontology analyses were used to annotate the upregulated genes (FDR < 0.05 and logFC>2) by clusterProfiler package^24^.

### Construction of a macrophage-related prognostic model

Macrophage differential related genes (MDRGs) were obtained from previous analysis by monocle package. A total of 105 genes were obtained by intersection of MDRG and DEG. Then, METABRIC and TCGA cohorts were used as training cohort and external test cohort, respectively. Least absolute shrinkage and selection operator (LASSO)-Cox regression analysis was performed to screen valuable genes for prognostic model construction. In detail, nlambda was set as 100, alpha was set as 1. By selecting the optimal penalty parameter λ associated with minimum 10-fold cross-validation, the prognostic model was constructed by six genes. Then, the coefficient of each gene was calculated by multivariate COX regression for the prognostic model construction. The risk score of each sample was defined as 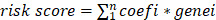. In detail, n represents number of genes, and coef_i_ and gene_i_ represent the corresponding coefficient and expression of each gene. The receiver operating characteristic (ROC) curves were conducted to estimate the predictive accuracy of 3-, 5-, 7- years overall survival (OS) for TNBC patients by survivalROC package. The Kaplan-Meier curves were utilized to estimate the OS of low- and high- risk groups. The rms package was utilized to construct the nomogram based on risk score, and calibration curves were performed to assess the predictive effectiveness of the model. Subsequently, decision curve analysis was performed by dcurves package to estimate the clinical benefit of the prognostic model.

### Clinical characteristics analyses

The correlations between risk score and clinical characteristics including recurrence-free survival, claudin subtype, Nottingham prognostic index, tumor laterality, and age were further analyzed in METABRIC dataset. For multiple categorical variables (intrinsic molecular subtype, Nottingham prognostic index), the Kruskal-Wallis test was performed to examine the differences of risk scores in these clinical characteristics. For binary categorical variables (tumor laterality, age), the Wilcoxon test was applied to examine the differences of risk scores in these clinical characteristics. A value with p< 0.05 was considered a statistical difference.

To explore the relationship between risk scores and more immune cells, the CIBERSOFTx was used to infer the immune cell enrichment score in METABRIC dataset. The spearman correlation analysis was conducted to evaluate the correlations. Values with P < 0.05 were considered statically significant.

### Connectivity map analysis

To screen potential drug for high-risk score group in TNBC, differential expression analysis was performed by limma package^25^. Gene ontology (GO) and KEGG pathways enrichment analysis were applied to upregulated genes in high-risk group. Then, a total of 300genes including the top 150 up-regulated and the top 150 down-regulated genes of high-risk group were uploaded on Connectivity Map database. L1000 platform^26^ was chosen to analyzed the potential drugs and mechanisms.

#### TNBC specimen

The TNBC (N=20) and paired adjacent normal tissues were collected at Tianjin Medical University Cancer Institute and Hospital, and were histologically confirmed as TNBC. This study was approved by the Ethical Committee of Tianjin Medical University Cancer Institute and Hospital and was consistent with the ethical guidelines of the Helsinki Declaration. Written informed consent was acquired from all involved patients.

### RT-qPCR

The total RNA from tissue was isolated using TRIzol Reagent (Invitrogen, USA) according to the manufacturer's protocols. The concentration and quality of RNA were detected by NanoDrop 2000 spectrophotometer (Thermo Scientific, USA). RT-qPCR (Real-time polymerase chain reaction reactions) assay was conducted using SYBR Green PCR Master Mix (TransGen Biotech, Beijing, China) on the 7500 Real-Time PCR System (Applied Biosystems, Waltham, MA, USA). The details of primers were established in supplementary Table S1.

### Statistical analyses

The results of RT-qPCR were analyzed using Prism 8 (Graph pad Software, CA) and showed as mean ± SD (standard deviation). Each experiment was included at least three individuals. The statistical significance was set as p < 0.05, which was determined with the unpaired, two-tailed Student t-test. The rest statistical analyses in the current study were performed by R Studio (version 4.2.3). All details about statistical analyses for each assay had been fully described in the corresponding section.

## Results

### Cell identification and clustering in TNBC scRNA-seq dataset

**Figure 1:**
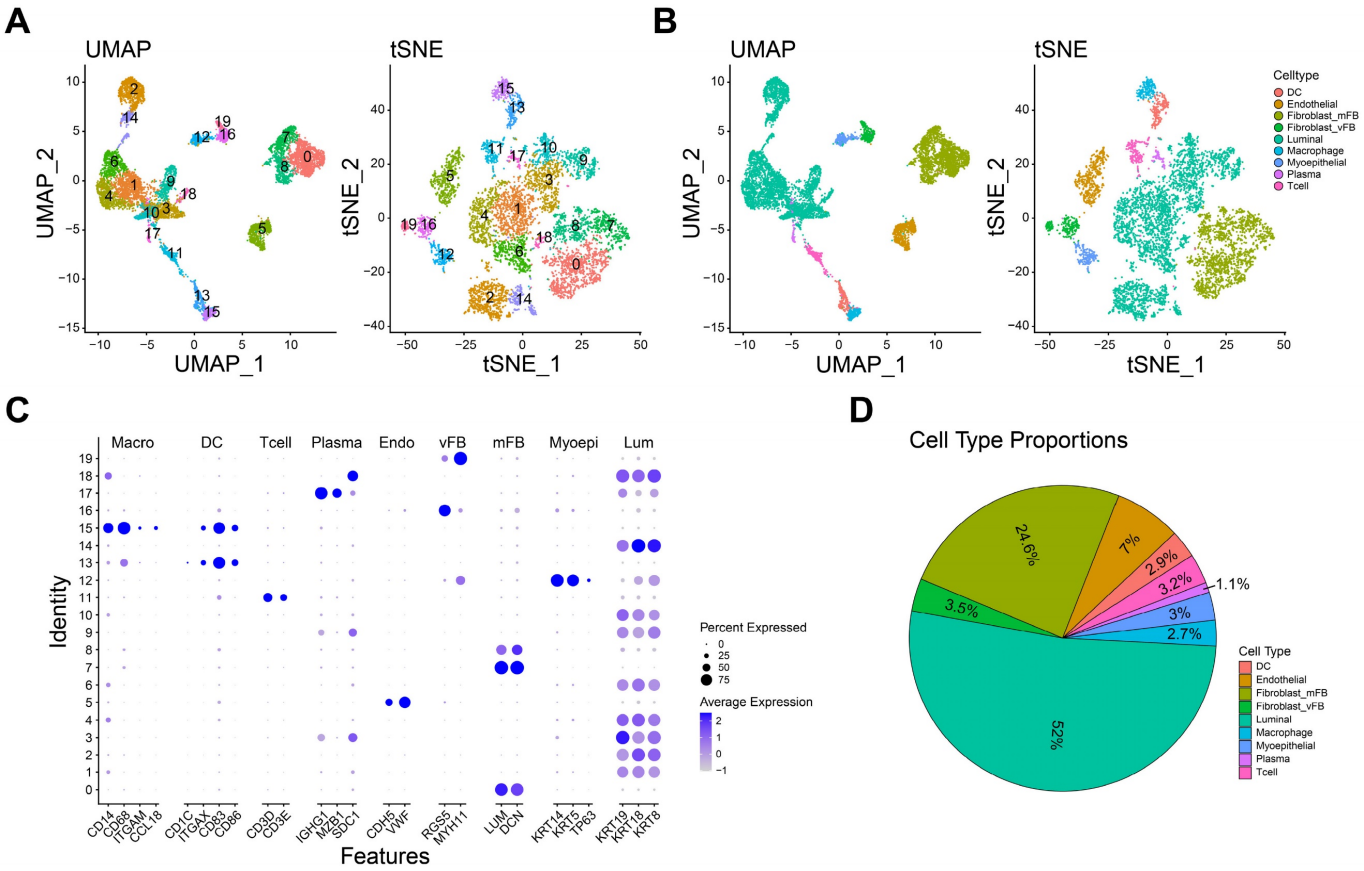
Distinct cell types in TNBC were identified through single-cell sequencing. **A-B** The cell clusters (A) and cell types (B) in TNBC tissue demonstrated using the Uniform Manifold Approximation and Projection (UMAP) and t-Distributed Stochastic Neighbor Embedding (TSNE) plots according to their featured gene expression profiles.** C** Dot plot displaying the expression level of marker genes for annotating the cell types.** D** The cell type portions in scRNA-seq dataset.

The expression profilers of scRNA-seq dataset were initially preprocessed to obtain the high-quality cells as the method mentioned. A total of 7993 single cells derived from 3 TNBC samples were included in the further analyses. Subsequently, we removed the batch effect by harmony package. Then, the components of cells were calculated by PCA and visualized by t-SNE and UMAP. As shown in Figure 1A, 20 cell clusters were identified when the resolution was set as 0.9. And nine main cell types were further defined by the specific cell marker genes: dendritic cell (*ITGAX, CD1C, CD83, CD86*), endothelial cell (*CDH5, VWF*), myofibroblast (*LUM, DCN*), vascular fibroblast (*RGS5, MYH11*), luminal cell (*KRT8, KRT19, KRT18*), macrophage (*CD14, CD68, ITGAM, CCL18*), myoepithelial cell (*KRT5, KRT14, TP63*), plasma (*IGHG1, MZB1, SDC1*) and T cell (*CD3D, CD3E*, Figure 1B-C). Furthermore, the marker genes used for cell identification clearly distinguish different cell types. The proportion of each cell in this dataset is shown in the figure 1D, and luminal cells had the highest proportion. Taken together, nine main cell types were identified for further analyses.

### Macrophage characteristics in TNBC

**Figure 2:**
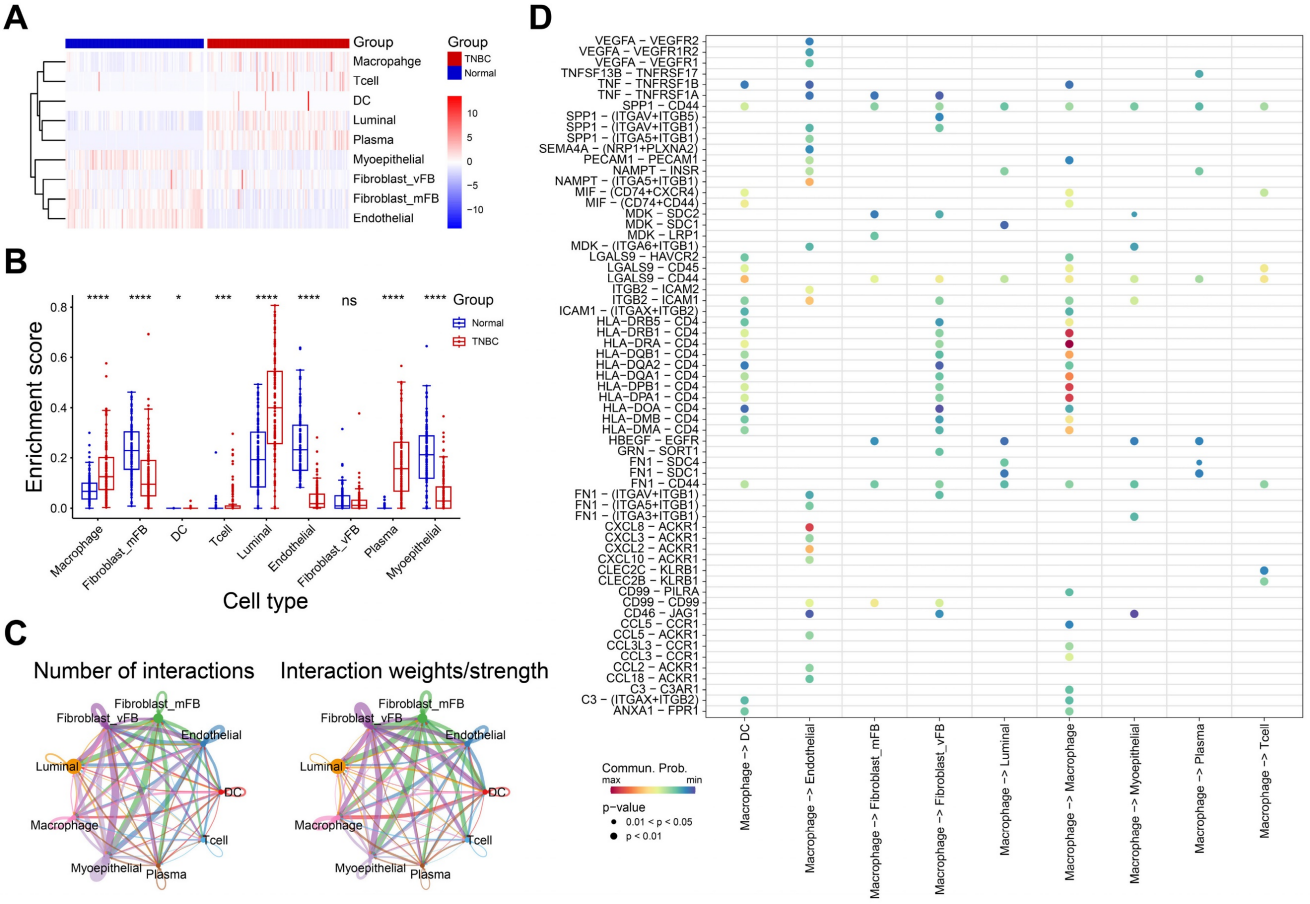
The role of macrophage in TNBC microenvironment. **A-B** The heatmap (A) and boxplot (B) showing the enrichment scores of each type of immune cells in normal and TNBC samples. **C** The interaction network diagrams showing the number and strength of the interactions among cell types.** D** The ligand-receptor pairs in macrophage and other cell types. *P< 0.05, **P < 0.01, ***P <0.001, ****P< 0.0001.

To investigate the immune cell alteration in TNBC microenvironment, we used the single cell RNA matrix to infer the cell proportions in bulk RNA data (TCGA-BRCA normal and TNBC samples). As shown in Figure 2A-B, the enrichment score of macrophage, luminal cell and plasma in TNBC samples were strongly higher than normal samples, while myoepithelial cell, endothelial cell and fibroblast were opposite. Luminal cell has been confirmed as an important source of breast cancer tumor cells^27^. These results implied the macrophage and luminal cell may play an essential role in TNBC progression.

Subsequently, we calculated the cell-cell interactions among nine cell types. As shown in Figure 2C, the number and strength of cell communication between macrophages and vascular fibroblasts, endothelial cells and dendritic cells were significantly higher than other cells. In detail, macrophages might interact with endothelial cells through *VEGF*-, and *CXCL*- related ligand-receptor pairs (Figure 2D). Also, we observed that macrophages interacted with vascular fibroblast via *SPP1*-, and *CD44*- related ligand-receptor pairs. Endothelial cells and fibroblasts play an important role in tumor metastasis and angiogenesis^28^, ^29^. These finding suggested macrophages may promote tumor progression and metastasis via interacting with endothelial cells and vascular fibroblasts. Of note, extensive *HLA-CD4* receptor-ligand interactions between macrophages was observed, suggesting that macrophages are not only interacting with other immune cells but are also actively engaging in autocrine and paracrine signaling to modulate the immune microenvironment.

### Re-clustering macrophages and inferring the pseudotime trajectory of TNBC macrophages

**Figure 3:**
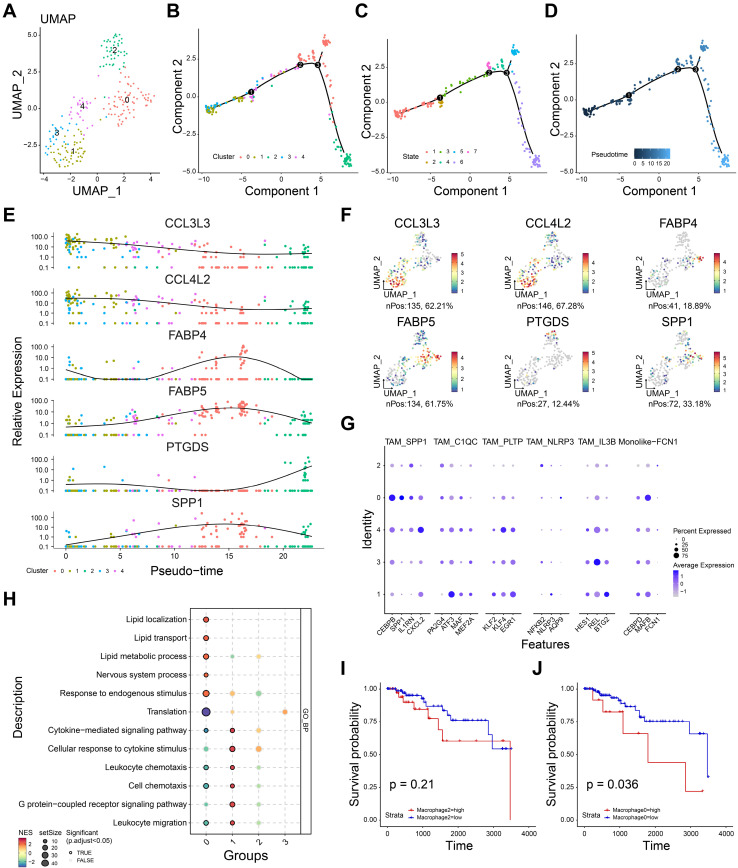
The heterogeneity of macrophage in TNBC microenvironment. **A** The UMAP dimensionality reduction graph of the distinct cell clusters.** B-D** The cell trajectory of cluster (B), state (C), pseudotime (D) for macrophages. **E** The relative gene expression of six macrophage-related genes in five distinct clusters. **F** The feature plot showing the gene expression of six macrophage-related genes. **G** The expression of tumor-associated macrophage markers in five clusters. **H** The gene set enrichment analysis showing the biologic function of each macrophage clusters. **I** The K-M curves showing the survival rate of high- and low- subgroup 2 groups. **J** The K-M curves showing the survival rate of high- and low- subgroup 0 groups. *P< 0.05, **P < 0.01, ***P <0.001, ****P< 0.0001.

To deeply understand the role of macrophages in TNBC tumor microenvironment, we re-clustered the macrophages, and five cell subgroups were identified (Figure 3A). Monocle package was applied to infer the potential cell trajectory of macrophages during the tumor progression. As shown in Figure 3B, five subgroups of macrophages were clearly separated on the cell trajectory. And the macrophage subgroups could be defined as seven different states (Figure 3C). Importantly, pseudotime trajectory of macrophages was mapped (Figure 3D). We observed that subgroups 0 and 2 were at the end of the trajectory, while subgroups 1 and 3 were at the beginning of the trajectory. These findings indicated the subgroups 1 and 3 may develop into subgroups 0 and 2 with the progression of TNBC. Six most important genes associated with pseudotime trajectory were identified (Figure 3E-F). For instance, *CCL3L3*, *CCL4L2* were highly expressed in subgroups 1 and 3. *SPP1*, *FABP4*, *FABP5* were highly expressed in subgroup 0 and *PTGDS* were highly expressed in subgroup 2.

Then, we used the reported markers of tumor-associated macrophage (TAM) to further annotate the five subgroups. As shown in Figure 3G, subgroup 0 showed a high expression of *SPP1+* TAM, suggesting subgroup 0 might be *SPP1+* macrophages. Consistently, six biomarkers of M2 macrophage were used estimate the function of five cell subgroups (Figure S1). M2-related markers were expressed in all subgroups except subgroup 3, suggesting that these subgroups (0, 1, 2, 4) may tend to express the characteristics of M2-like cells. Enrichment analysis showed subgroups 0 and 2 were negative with leukocyte chemotaxis, cell chemotaxis, leukocyte migration, indicating the immunosuppressive role of these subgroups (Figure 3H). Interestingly, subgroup 0 showed high enrichment scores in lipid-related processes including lipid transport, lipid metabolic process and lipid localization. Subgroup 1 showed high enrichment scores in leukocyte chemotaxis and migration, suggesting it may be correlated with inflammatory activation. Furthermore, we performed the survival analyses to evaluate the prognostic effect of subgroups 0 and 2. As shown in Figure 3I, high infiltration of subgroup 2 showed no significant difference in prognosis (P =0.21). And high infiltration of subgroup 0 was associated with poor prognosis (P= 0.036, Figure 3J). Taken together, subgroup 0 was related to immunosuppression and could be a potential prognostic predictor for TNBC patients.

### Construction of macrophage-related prognostic model

**Figure 4:**
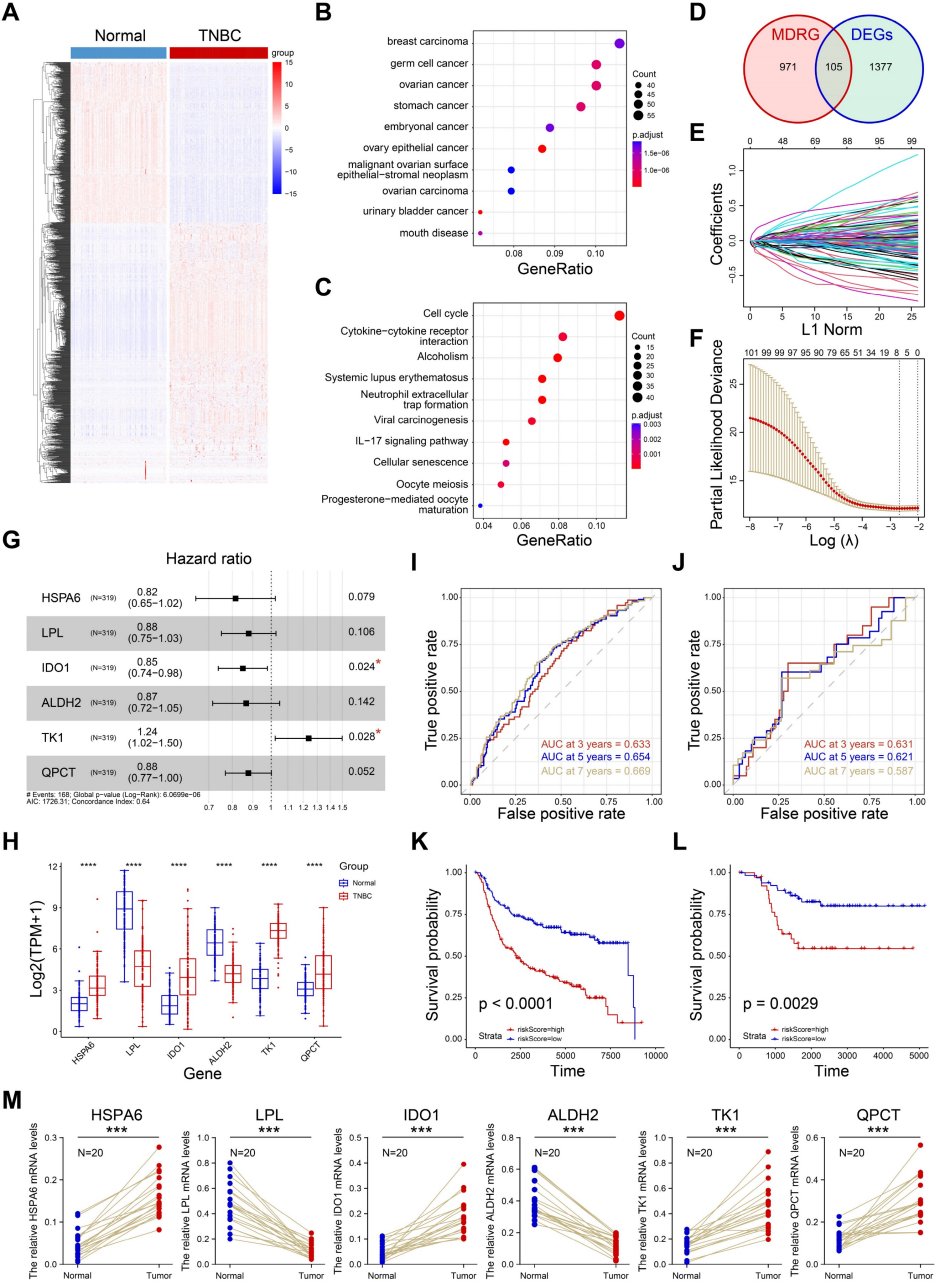
Construction of macrophage-related prognostic model. **A** Heatmap of differential expression genes in TNBC and normal samples. **B-C** Disease ontology and KEGG pathway analyses of up-regulated genes. **D** The venn diagram showing the intersection of MDRG and DEGs. **E** Lasso Cox regression analysis. **F** Partial likelihood deviance for the Lasso regression. **G** Multivariate Cox analysis of six candidate genes. **H** Expression of six genes in normal and tumor tissues. **I-J** The ROC curves of 3-, 5-, 7- years OS in both training (I) and external datasets (J). **K-J** The K-M curves showing the overall survival rate of high- and low- risk groups. **M** The RT-qPCR results showing the relative expression of six genes in normal and tumor groups.

The prognostic value of macrophage has been demonstrated in a large number of studies. To construct a macrophage-related prognostic model for TNBC prognostic stratification, we firstly performed the differential expression analysis between normal samples and TNBC samples (Supplementary Table S2). A total of 1382 DEGs were obtained and visualized in Figure 4A. Subsequently, 846 up-regulated genes were utilized to performed enrichment analyses. As shown in Figure 4B, the up-regulated genes were enriched in breast carcinoma, indicating the reliability of the differential analysis we completed. Next, we the KEGG pathway analysis showed the up-regulated genes were closely associated with cell cycle, cytokine-cytokine receptor interaction and cellular senescence (Figure 4C). Macrophage differential-related genes (MDRG) was shown in supplementary Table S3. After interaction of MDRG and DEGs, 105 common genes were collected (Figure 4D). Then, we performed LASSO regression to further screen candidate prognostic genes (Figure E-F). The multivariate Cox regression was performed and six prognostic genes (*HSPA6, LPL, IDO1, ALDH2, TK1, QPCT*) were identified for prognostic model construction (Figure 4G). Finally, the risk score of each sample was defined as: risk score= 0.214092*TK1_exp_ - 0.204042*HSPA6_ exp_ - 0.128893*LPL_ exp_ - 0.162297*IDO1_ exp_ - 0.143447* *ALDH2*_ exp_ - 0.13061*QPCT_ exp_ (Supplementary Table S4). As shown in Figure 4H, the expression of six genes were significantly different in normal and TNBC samples in TCGA cohort. The 3-, 5-, 7- years of AUCs in training dataset (N=319, METABRIC cohort) were 0.633, 0.654, and 0.669, respectively (Figure 4I). And 3-, 5-, 7- years of AUCs in external test dataset (N=107, TGCA cohort) were 0.633, 0.654, and 0.669, respectively (Figure 4J). These results indicated the prognostic model constructed by six genes was a good predictor for the overall survival of TNBC patients. In addition, we divided the training set and the external test set into high-risk and low-risk groups based on the best cutoff values of the risk scores. The high-risk group was closely associated with a poor prognosis in both training set (Figure 4K) and external test set (Figure 4L). And the RT-qPCR confirmed that six genes were differentially expressed in TNBC and normal tissues (Figure 4M). In a word, the prognostic model showed a good predictive effect on prognosis of TNBC patients and it could be a potential tool for prognosis stratification for TNBC.

### Evaluation of the prognostic model

**Figure 5:**
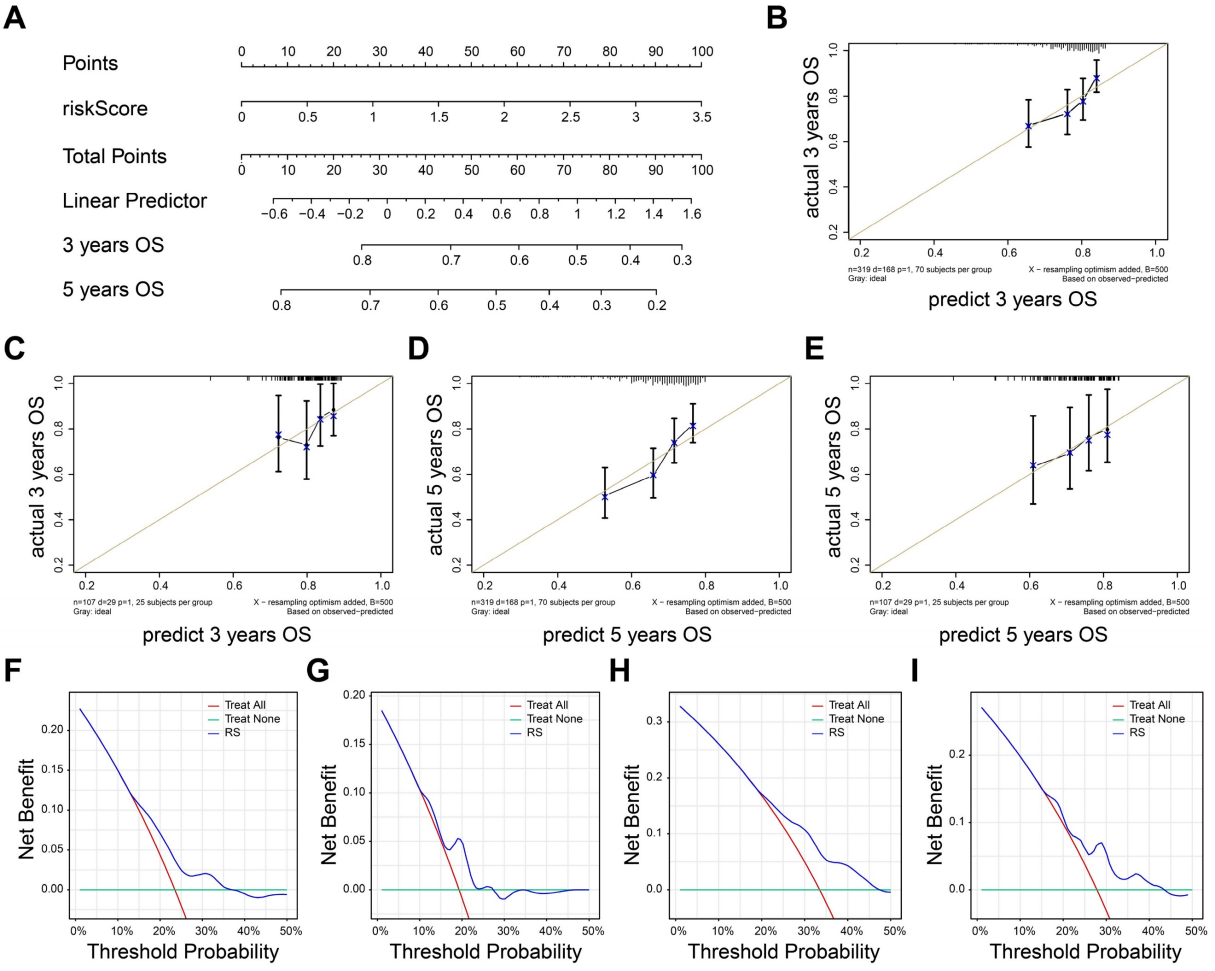
Evaluation of the prognostic model. **A** The nomogram based on risk score. **B-C** The calibrate curves to evaluate the consistency of predicted and actual 3 years OS in training (B) and external cohorts (C). **D-E** The calibrate curves to evaluate the consistency of predicted and actual 5 years OS in training (D) and external cohorts (E). **F-G** 3-year survival benefit in training (F) and external cohorts (G). **H-I** 5-year survival benefit in training (H) and external (I) cohorts.

Nomogram was established by rms package to visualize predictive possibility of risk score for 3-, 5- years OS (Figure 5A). The risk score of patients can be calculated by formula we constructed and corresponding to the total points, so as to evaluate the 3-5-year survival rate of patients. The risk score could be Calibrate curves were used to evaluate the predictive effect of model. The result showed that the prognostic model in training set (Figure 5B) and external test set (Figure 5C) had a good fit at 3-years OS. Also, the prognostic model also had a good fit in predicting 5-years overall survival of TNBC patients in both training set (Figure 5D) and external test set (Figure 5E).

We next perform decision curve analysis to investigate the clinical benefit of the nomogram. As shown in Figure 5F-G, the nomogram demonstrated a better clinical benefit for predicting 3-year OS in both training (Figure 5F) and external test set (Figure 5G). Consistently, the positive clinical benefits were also observed in 5-year OS in same datasets (Figure 5H-I). Together, the nomogram exhibited a great stability and accuracy in 3-, 5- years OS.

### Clinical application of the prognostic model

**Figure 6:**
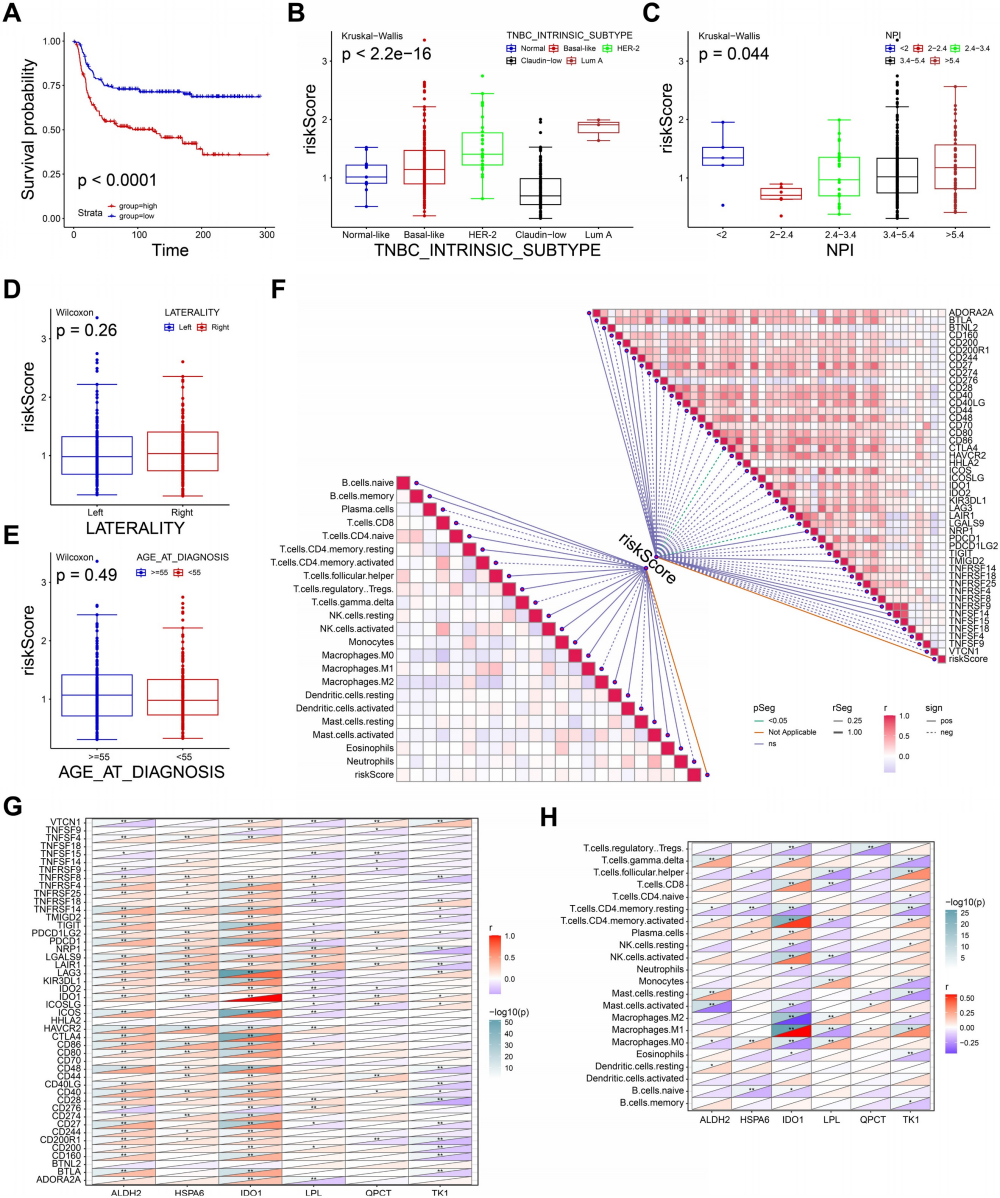
Clinical application of the prognostic model. **A** The K-M curve of recurrence-free survival in METABRIC dataset. **B-E** The boxplots showing the risk scores in intrinsic molecular subtype (B), NPI (C), tumor laterality (D), and age (E). **F** The diagram exhibiting the correlations between risk score and the expression of immune checkpoints, enrichment score of immune cells. **G** The correlations between the expression of six genes and immune checkpoints. **H T**he correlations between the expression of six genes and enrichment score of immune cells.

We next performed the survival analysis to evaluate the recurrence-free survival (RFS) for high-, low- risk groups in METABRIC dataset. As shown in Figure 6A, patients in high-risk group were associated with short RFS (P <0.0001). The definition of TNBC intrinsic molecular subtypes including Normal-like, Basal-like, Luminal A, Luminal B, HER2-enriched, and Claudin-low provides a theoretical basis for TNBC treatment^30^. Thus, we explored the differences in risk score across subtypes. The risk score of intrinsic molecular subtype of TNBC was significantly different, indicating the risk score may be an effective tool to assist the diagnosis of intrinsic typing of TNBC (P< 2.2e-16, Figure 6B). Nottingham prognostic index (NPI) is a practical tool to predict the prognosis of BRCA patients, and its parameters include tumor size, number of lymph nodes involved, and tumor grade^31^. As shown in Figure 6C, risk score was significantly different in distinct NPI groups (P=0.044). However, the risk score showed no significant differences in both tumor laterality (Figure 6D) and age (Figure 6E). These findings suggested the prognostic model had a good discrimination ability to distinguish between different subtypes and prognosis of TNBC.

Then, we performed spearman correlation analysis to estimate the correlation between immune infiltration, immune checkpoint (ICP) molecules and risk score. The risk score showed a strongly association with the expressions of *CTLA4* and *LGALS9*, however, it showed no significant correlation with immune cells infiltration (Figure 6F). Therefore, high-risk group may benefit from ICP treatment of *CTLA4* and *LGALS9*. Also, we further calculated the correlation between six genes of prognostic model. The expression of *ALDH2*, *HSPA6* was positively correlated the expression of most ICP (Figure 6G). Conversely, the expression of *TK1* and *LPL* was negatively correlated with the expression of most ICP. Interestingly, *IDO1* was a member of the immune checkpoint molecule and an important variable in our prognostic model. Since the genes in our prognostic model were derived from macrophage-related genes, we further explored the correlation between these genes and macrophage infiltration. As shown in Figure 6H, the expression of these genes was significantly associated with different macrophage infiltrations, indicating an important role in the polarization of macrophages. In summary, the prognostic model and the expression levels of its 6 genes may be an effective predictor of TNBC prognosis, immune infiltration and treatment.

### Identification of the potential drugs

**Figure 7:**
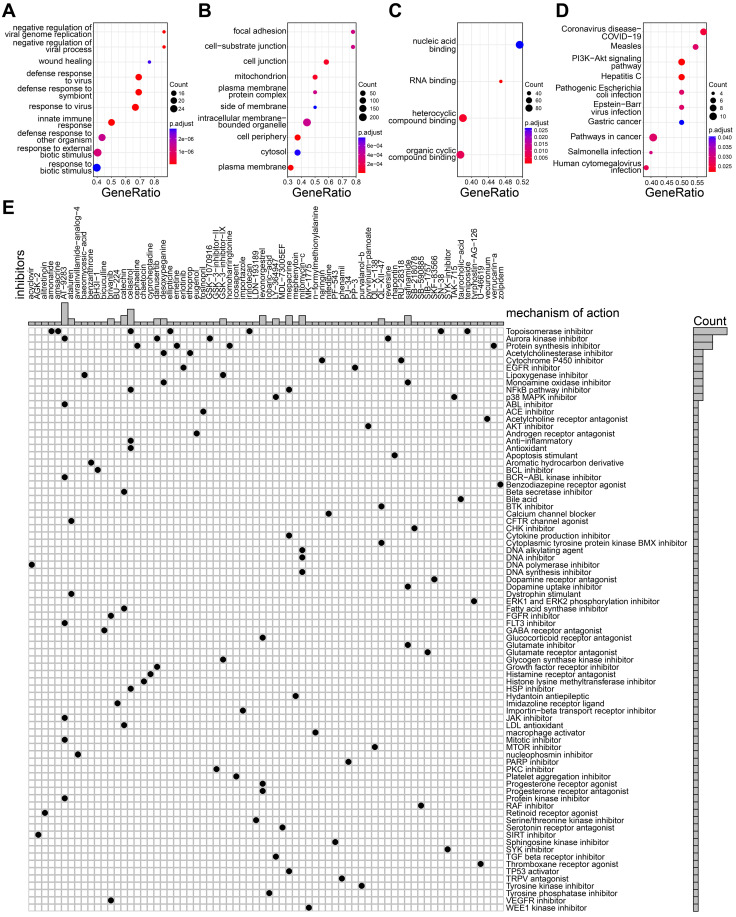
Exploration of candidate drugs. **A-C** The biologic process (A), molecular function (B), and cell component (C) of up-regulated genes in high-risk group. **D** The KEGG pathways of up-regulated genes in high-risk group. **E** The heatmap showing the potential drugs and their mechanisms of action for high-risk group.

We further performed the differential expression analysis to identify DEG between high-, and low- risk groups using limma package. The result of differential analysis was presented in supplementary Table S5. The up-regulated genes were used to perform GO and KEGG enrichment analyses. As shown in Figure 7A-C, the up-regulated genes in high-risk group were involved in wounding healing, focal adhesion, cell junction, cell-substrate junction, RNA-binding, suggesting high-risk group may be associated with tumor metastasis. In addition, the pathways including pathways in cancer, *PI3K*-*AKT* signaling pathway were highly enriched in high-risk group (Figure 7D). The findings implied the patients with high-risk group probably promote tumor progression via *PI3K*-*AKT* pathways. Targeting *PI3K*-*AKT* pathways can be a potential way for TNBC treatment. The high-risk TNBC patients showed a poor prognosis, therefore, more potential drugs need to be developed for improving their prognosis. Connectivity Map (Cmap) analysis was applied to filter candidate drugs by calculating up- and down- regulated genes in high- risk TNBC patients. All drugs are analyzed by Cmap and get a value between -100 and 100. A smaller value indicates a stronger potential inhibitory effect. As shown in Figure 6E, the candidate drugs (values < -70) with their mechanism of action were selected for high-risk TNBC patients. For example, acyclovir, as a DNA polymerase inhibitor, was identified as one of the candidate drugs. Taken together, the drugs we screened may serve as potential treatments for patients at high-risk of TNBC.

## Discussion

The absence of Her-2 amplification and lack of expression of hormone-related proteins have made chemotherapy the only systemic standard treatment for TNBC patients^6^, ^32^. Additionally, TNBC is a heterogeneous disease on clinical, pathologic, and molecular levels^33^. Exploring the heterogeneity of TNBC and developing patient risk stratification and individualized intervention therapy may be potential strategies to improve the survival rate of TNBC patients in the future. The emergence of single-cell sequencing makes it possible to understand tumor heterogeneity at the cellular level and develop new therapeutic strategies through this technology.

In this study, we comprehensively analyzed the role of tumor-associated macrophages in TNBC by integrating single cell and bulk RNA datasets. We annotated the cell types in single cell RNA dataset to inferred the cell proportions in normal and TNBC bulk RNA dataset. We observed the macrophages were significantly higher enriched in TNBC than normal samples. This finding indicated the status and number of macrophages in TNBC changed compared to normal tissues and played a role in the tumor microenvironment. By further analyses of cell-cell interactions, we observed the strong interactions between macrophages and endothelial cells through *VEGF* and *CXCL/ACKR1* signaling pathways. *VEFG* signaling pathway plays an important role in tumor angiogenesis, and tumor growth^34^. Atypical chemokine receptor 1 (*ACKR1*), is known as a core regulator which binds chemokines involved in inflammatory responses and tumor proliferation, angiogenesis, and metastasis^35^. Macrophages may promote tumor progression and formation of suppressive microenvironment by interacting with endothelial cells. Meanwhile, *SPP1* signaling pathway which involved in tumor growth and metastasis was activated in vascular fibroblast by macrophage, indicating macrophage may help mediate tumor proliferation^36^. This discovery provides a novel insight into immunotherapy for TNBC.

After further clustering of macrophages, five distinct types of macrophages were identified. It is worth noting that we identified *SPP1*+ TAMs as one of the end-state macrophages during the TNBC progression. Lipid metabolism related markers including *FABP4*, *FABP5* were also highly expressed in *SPP1*+ TAMs. A previous study has reported that a special subgroup of macrophages which exhibited a canonical signature of M2-like TAMs distributed in tumor-adipose junctional regions in BRCA patients^37^. Consistent with our findings, the macrophages reported also showed the same characteristics including high expression of metabolism-related marker genes and poor prognostic effect with macrophages we identified. Our results highlighted that SPP1+ cells also exhibited a signature of inhibiting immune cell infiltration, suggesting an immunosuppressive and tumor escape role in the TNBC tumor microenvironment. In addition, a large number of studies also identified the *SPP1*+ TAMs in other tumors. For example, Wei *et al.*^38^ found *SPP1*+ TAMs potentially enhance epithelial-mesenchymal transition by interaction with cancer cells through paracrine pattern in multiple cancers. Qi *et al.*^39^ demonstrated the interactions between *SPP1*+ TAMs and *FAP*+ fibroblasts stimulate the formation of immune-excluded desmoplasic structure and limit the T cell infiltration. Taken together, our findings preliminarily describe the function of TAMs in TNBC and increase understanding of the TNBC tumor microenvironment.

Up-regulated genes in TNBC and MRDG were obtained to constructing a prognostic model. Up-regulated genes were mainly involved in breast carcinoma and cell cycle, indicating the accuracy of the analyses. Meanwhile, it indicated that cell cycle pathway was essential for TNBC progression. The prognostic model was constructed after a series of selections. We observed that the model had good differentiation and accuracy in predicting 3-, 5-, 7- years overall survival in TNBC patients. Furthermore, high- and low- risk group showed extremely different prognosis (OS and RFS) in both training and external test group. Nottingham prognostic index is a common evaluation for BRCA patients^31^. Our results showed risk scores was increased with NPI except the group with NPI < 2.0. This may be caused by the limited sample size. Consistent with clinical significance of NPI, high-risk scores associated with poor prognosis. Together, the prognostic model we constructed is a potential tool for prognostic stratification of TNBC. Meanwhile, high expression of *CTLA4* and *LGALS9* was associated with risk scores, suggesting high-risk group may benefit from the immunotherapy derived from these two immune checkpoints. These findings provide an idea for personalized immunotherapy for TNBC patients. To further find therapeutic targets for high-risk patients, we analyzed differences in the transcriptomes of high- and low- risk patients.

*PI3K*-*AKT* signaling pathway was high enriched in high-risk group. As a star pathway, it is reported to be a critical role in tumor growth and survival in cancers^40^. Finally, we used Connectivity Map database by uploading the DEGs based high- and low- risk group. And the inhibitors and their mechanisms were identified for high-risk group patients. Although the effects of these drugs still need to be confirmed in further experiments and clinical trials, their efficacy in TNBC treatment has been consolidated in previous reports. For example, celastrol with several mechanisms of act, has been confirmed to suppress TNBC progression^41^-^43^. Alitretinoin (9-cis-retinoic acid), was already being test in breast cancer clinical trials^44^. These discoveries provide new insights into the individualized treatment of TNBC.

Bao *et al.*^14^ reported the macrophages in TNBC by integrating single cell and bulk RNA sequencing. They established a TAM-related gene signature for predicting prognosis and response to immunotherapy. In contrast to this study, we collected more single cell expression profiles and highlighted the heterogeneity of macrophages and their interactions with other cells, which makes the understanding of macrophages in the tumor microenvironment more comprehensive and general. More importantly, we provide potential therapeutic drug options for patients at high-risk based on our prognostic model, which extend the value for the clinical transformation of TNBC individualized drug therapy in the future. However, this study also had some limitations. First, the sample sizes including single cell RNA data and external test datasets should be further expanded to establish the robustness of the prognostic model. In addition, no experiments were conducted to validate the potential role of *SPP1*+ macrophage. We will collect more clinical samples with complete information to validate our model. And *in vivo* and *in vitro* experiments will be conducted to explore the therapeutic effect of drugs.

## Conclusion

In this study, we identified the five distinct types of tumor-associated macrophages during the TNBC progression. And we established a macrophage-related prognostic model for prognostic risk stratification. Ultimately, several drugs were identified as potential choices for high-risk TNBC patients. Our findings potentially provide value in not only the understanding of tumor-associated macrophages in TNBC but also the translational application of TNBC risk stratification.

## Supplementary Material

Supplementary figure and tables.

## Abbreviations

BRCA: Breast cancer
LASSO: Least Absolute Shrinkage and Selection Operator
TCGA: The Cancer Genome Atlas Program
GEO: Gene Expression Omnibus
OS: overall survival
RFS: Recurrence free survival
TNBC: Triple-negative breast cancer
TAM: Tumor-associated macrophage
GSEA: Gene set enrichment analysis
GO: Gene ontology
KEGG: Kyoto Encyclopedia of Genes and Genomes
DEG: differential expression genes
ROC: Receiver operating characteristic
NPI: Nottingham prognostic index
