# Supplementary material for: Integrating Bulk and Single-cell RNA-seq to Construct a Macrophage-related Prognostic Model for Prognostic Stratification in Triple-negative Breast Cancer
Source: J Cancer. 2024 Sep 23;15(18):6002–15. doi: 10.7150/jca.101042 (PMC11493015; doi:10.7150/jca.101042)
Supplement: Supplementary file 1 — Supplementary figure and tables. [file jcav15p6002s1.zip › Supplementary File/Supplementary Figure.docx]

**Supplementary Figure:**


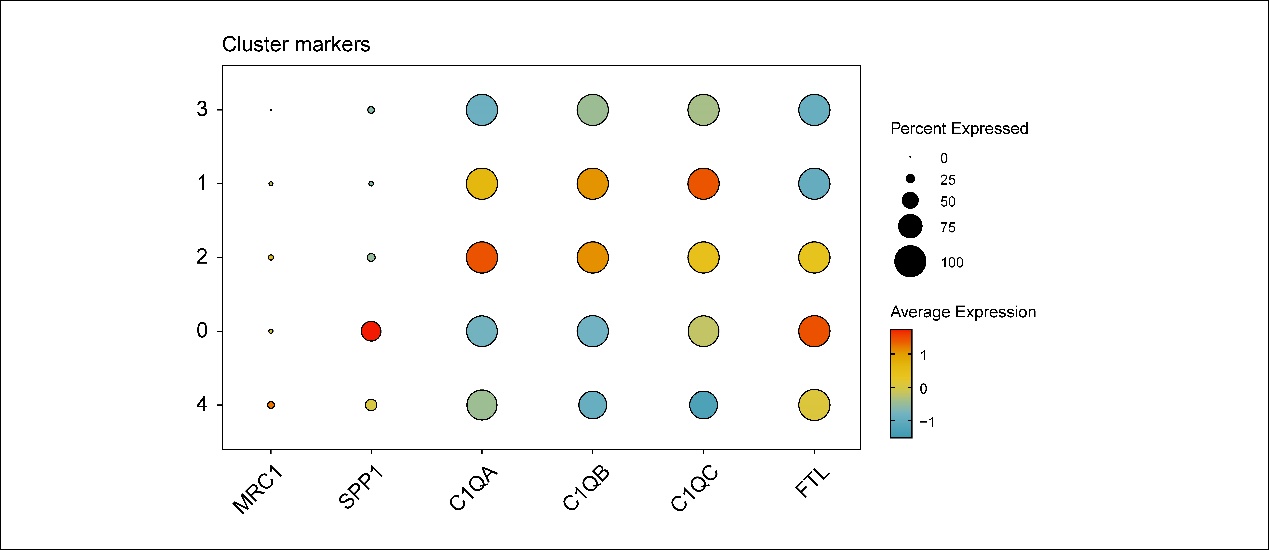


**Figure S1:** The markers used to annotate M2 macrophages. The average expression of M2-macrophages marker in five subgroups.
