# Supplementary material for: Integrating Bulk and Single-cell RNA-seq to Construct a Macrophage-related Prognostic Model for Prognostic Stratification in Triple-negative Breast Cancer
Source: J Cancer. 2024 Sep 23;15(18):6002–15. doi: 10.7150/jca.101042 (PMC11493015; doi:10.7150/jca.101042)
Supplement: Supplementary file 1 — Supplementary figure and tables. [file jcav15p6002s1.zip › Supplementary File/Table S1 Primers of candidate genes.docx]

| **Table S1 Primers of candidate genes** | |
| --- | --- |
| **HSPA6** |  |
| **Forward Primer** | GATGTGTCGGTTCTCTCCATTG |
| **Reverse Primer** | CTTCCATGAAGTGGTTCACGA |
| **LPL** |  |
| **Forward Primer** | TCATTCCCGGAGTAGCAGAGT |
| **Reverse Primer** | GGCCACAAGTTTTGGCACC |
| **IDO1** |  |
| **Forward Primer** | GCCAGCTTCGAGAAAGAGTTG |
| **Reverse Primer** | ATCCCAGAACTAGACGTGCAA |
| **ALDH2** |  |
| **Forward Primer** | ATGGCAAGCCCTATGTCATCT |
| **Reverse Primer** | CCGTGGTACTTATCAGCCCA |
| **TK1** |  |
| **Forward Primer** | GGGCAGATCCAGGTGATTCTC |
| **Reverse Primer** | TGTAGCGAGTGTCTTTGGCATA |
| **QPCT** |  |
| **Forward Primer** | GGAACAACAGAGTGTTTGTAGGA |
| **Reverse Primer** | TGTCGAGACCCATAGAGAGAATC |
