# Supplementary material for: Integrating Bulk and Single-cell RNA-seq to Construct a Macrophage-related Prognostic Model for Prognostic Stratification in Triple-negative Breast Cancer
Source: J Cancer. 2024 Sep 23;15(18):6002–15. doi: 10.7150/jca.101042 (PMC11493015; doi:10.7150/jca.101042)
Supplement: Supplementary file 1 — Supplementary figure and tables. [file jcav15p6002s1.zip › Supplementary File/Table S2 Up-regulated and down-regulated genes in TGCA-TNBC dataset.docx]

| **Table S2 Up-regulated and down-regulated genes in TGCA-TNBC dataset** | | | | |
| --- | --- | --- | --- | --- |
|  | logFC | logCPM | PValue | FDR |
| **CASP14** | 7.864624554 | 6.138096962 | 2.16E-39 | 1.62E-38 |
| **ACTL8** | 7.853976725 | 4.522572693 | 3.2E-55 | 4.52E-54 |
| **MMP1** | 7.776968395 | 6.442294603 | 2.74E-98 | 1.64E-96 |
| **MAGEA3** | 7.30481899 | 4.027798188 | 8.87E-35 | 5.55E-34 |
| **PAGE2** | 7.018508533 | 3.780460603 | 7.77E-25 | 3.19E-24 |
| **SMR3B** | 7.01816371 | 8.466967393 | 2.55E-20 | 8.56E-20 |
| **MMP13** | 6.895238157 | 5.558233671 | 1.33E-60 | 2.23E-59 |
| **MAGEA4** | 6.860099595 | 3.973091453 | 8.37E-28 | 3.89E-27 |
| **CSAG1** | 6.845778011 | 3.341660894 | 1.16E-32 | 6.66E-32 |
| **MAGEA6** | 6.819552144 | 3.759554943 | 4.97E-30 | 2.56E-29 |
| **CT83** | 6.658296964 | 4.837423907 | 3.01E-63 | 5.66E-62 |
| **PAGE1** | 6.646936491 | 3.172061246 | 2.9E-21 | 1.02E-20 |
| **ORM1** | 6.531496721 | 5.296709363 | 8.65E-37 | 5.89E-36 |
| **FTHL17** | 6.478310245 | 2.98862131 | 2.48E-23 | 9.51E-23 |
| **SMR3A** | 6.472630948 | 3.959486659 | 1.25E-20 | 4.26E-20 |
| **PRR27** | 6.368401291 | 3.144845444 | 4.97E-26 | 2.14E-25 |
| **TLX1** | 6.181908172 | 3.18391778 | 7.56E-59 | 1.21E-57 |
| **CA9** | 6.162889821 | 4.808396963 | 5.84E-80 | 1.88E-78 |
| **MAGEA12** | 6.077591422 | 2.78807014 | 7.31E-23 | 2.76E-22 |
| **NCAN** | 6.070944797 | 3.46727215 | 8.94E-25 | 3.66E-24 |
| **XAGE2** | 6.068306545 | 3.988024735 | 1.35E-31 | 7.42E-31 |
| **CLDN6** | 6.06766342 | 3.758664306 | 4.71E-37 | 3.23E-36 |
| **HORMAD1** | 5.957768583 | 4.1616002 | 1.9E-65 | 3.86E-64 |
| **ACAN** | 5.934598329 | 4.004922669 | 4.59E-46 | 4.46E-45 |
| **VCX3A** | 5.862769995 | 2.565829251 | 2.52E-20 | 8.45E-20 |
| **CST1** | 5.835642288 | 4.373700987 | 8.46E-49 | 9.28E-48 |
| **ORM2** | 5.816790663 | 5.076572687 | 2.19E-42 | 1.85E-41 |
| **IBSP** | 5.816700714 | 2.945353764 | 2.32E-71 | 5.76E-70 |
| **MMP11** | 5.780873573 | 7.025173868 | 2.35E-122 | 2.34E-120 |
| **COL10A1** | 5.779448157 | 5.313899941 | 1.86E-117 | 1.7E-115 |
| **PRAME** | 5.744090212 | 5.126787479 | 6.2E-63 | 1.15E-61 |
| **PRSS33** | 5.713054424 | 3.577763276 | 1.47E-38 | 1.07E-37 |
| **MSLN** | 5.683818247 | 6.365009277 | 2.74E-44 | 2.5E-43 |
| **HAPLN1** | 5.602066325 | 2.984578311 | 8.93E-45 | 8.27E-44 |
| **KIF1A** | 5.557545473 | 3.388056481 | 9.54E-57 | 1.42E-55 |
| **COL11A1** | 5.547572744 | 5.257799495 | 6.11E-107 | 4.54E-105 |
| **MAGEC2** | 5.542753277 | 2.514616363 | 9.75E-21 | 3.35E-20 |
| **ZFP42** | 5.50057113 | 2.692619877 | 1.99E-23 | 7.65E-23 |
| **KRT75** | 5.468566751 | 3.126377271 | 3.08E-35 | 1.97E-34 |
| **SBSN** | 5.439771362 | 3.950131313 | 1.58E-40 | 1.24E-39 |
| **H2BC14** | 5.386753045 | 2.352650883 | 3.48E-27 | 1.58E-26 |
| **C1orf105** | 5.373651761 | 2.454108237 | 6.33E-25 | 2.61E-24 |
| **COL9A1** | 5.276257789 | 2.983733708 | 2.26E-32 | 1.28E-31 |
| **SPDYC** | 5.253738005 | 2.917047674 | 1.53E-38 | 1.11E-37 |
| **MMP9** | 5.237710946 | 8.742333132 | 1.32E-52 | 1.69E-51 |
| **SERPINB4** | 5.22350709 | 3.029611979 | 8.12E-27 | 3.63E-26 |
| **UBE2C** | 5.21498757 | 7.114602751 | 5.92E-213 | 6.19E-210 |
| **CCKBR** | 5.149614773 | 2.712357959 | 9.83E-43 | 8.42E-42 |
| **CXCL10** | 5.073723105 | 8.117389034 | 4.74E-92 | 2.27E-90 |
| **CRCT1** | 5.063444101 | 2.084350785 | 6.93E-19 | 2.16E-18 |
| **SERPINB7** | 5.053956258 | 3.21372587 | 1.19E-41 | 9.78E-41 |
| **SPRR1B** | 5.018901908 | 4.658839815 | 5.11E-17 | 1.47E-16 |
| **RTP3** | 4.985615846 | 2.284643405 | 1.16E-22 | 4.33E-22 |
| **REG1A** | 4.97407824 | 2.160221965 | 1.02E-17 | 3.03E-17 |
| **H3C2** | 4.967246864 | 2.56585996 | 2.29E-42 | 1.93E-41 |
| **GAGE2A** | 4.956224881 | 1.924763478 | 5.1E-16 | 1.41E-15 |
| **MYBL2** | 4.953751897 | 6.671594576 | 1.09E-169 | 3.13E-167 |
| **MAEL** | 4.942529313 | 2.558864729 | 8.14E-27 | 3.63E-26 |
| **HOXB13** | 4.865852487 | 2.666276268 | 1.63E-40 | 1.28E-39 |
| **CDC20** | 4.840258723 | 6.784156759 | 1.63E-219 | 2.17E-216 |
| **DMBT1** | 4.83738098 | 3.751311417 | 8.33E-21 | 2.87E-20 |
| **EZHIP** | 4.836915013 | 2.122646178 | 1.07E-21 | 3.83E-21 |
| **SSX1** | 4.821805661 | 2.066731236 | 3.19E-18 | 9.69E-18 |
| **COL2A1** | 4.816599542 | 6.155726475 | 3.65E-30 | 1.89E-29 |
| **KRT6C** | 4.781347501 | 3.92381052 | 1.69E-29 | 8.48E-29 |
| **CSAG3** | 4.769735942 | 2.059389099 | 1.83E-28 | 8.75E-28 |
| **COL9A3** | 4.766701062 | 5.261460968 | 4.53E-58 | 7.02E-57 |
| **SERPINB3** | 4.744952927 | 2.713489682 | 1.11E-21 | 3.97E-21 |
| **SIX3** | 4.733697964 | 3.50105009 | 7.87E-51 | 9.41E-50 |
| **COX7B2** | 4.730279923 | 1.970260224 | 1.24E-18 | 3.83E-18 |
| **NEK2** | 4.729644608 | 4.906288279 | 4.38E-225 | 7.11E-222 |
| **S100A8** | 4.721722024 | 8.683303676 | 2.9E-47 | 2.96E-46 |
| **MAGEA10** | 4.716312157 | 1.998895853 | 4.11E-19 | 1.3E-18 |
| **NMU** | 4.702980773 | 3.723771344 | 6.43E-69 | 1.48E-67 |
| **COL11A2** | 4.695776445 | 4.253968416 | 3.74E-41 | 3.01E-40 |
| **KRT6A** | 4.686337021 | 7.233206604 | 3.63E-26 | 1.57E-25 |
| **INA** | 4.650962726 | 2.440460006 | 1.07E-37 | 7.51E-37 |
| **FOXM1** | 4.631413438 | 5.414223546 | 1.26E-184 | 5.44E-182 |
| **DPYSL5** | 4.621737628 | 2.211627188 | 1.94E-25 | 8.11E-25 |
| **NUF2** | 4.618049782 | 4.617608036 | 3.97E-203 | 2.9E-200 |
| **GABRA5** | 4.617124722 | 1.950733006 | 3.07E-27 | 1.39E-26 |
| **EPYC** | 4.607365661 | 2.470090656 | 5.51E-53 | 7.21E-52 |
| **MELK** | 4.589695541 | 5.022448989 | 2.08E-206 | 1.6E-203 |
| **MLC1** | 4.57079062 | 3.474555942 | 2.87E-30 | 1.5E-29 |
| **TPX2** | 4.555169804 | 6.595836623 | 1.57E-245 | 1.15E-241 |
| **KIF4A** | 4.55092305 | 4.656449769 | 6.91E-232 | 2.53E-228 |
| **CXCL11** | 4.545717843 | 5.115857697 | 3.72E-83 | 1.33E-81 |
| **POU3F2** | 4.545657317 | 2.061759773 | 4.21E-20 | 1.4E-19 |
| **BARX1** | 4.541621574 | 3.698653844 | 1.98E-36 | 1.32E-35 |
| **GJB2** | 4.536190196 | 5.591262316 | 1.1E-86 | 4.4E-85 |
| **AURKB** | 4.529312191 | 5.056472468 | 5.68E-209 | 4.62E-206 |
| **IQGAP3** | 4.524457733 | 4.743865032 | 5.92E-176 | 2.06E-173 |
| **KRT79** | 4.523228359 | 4.39774065 | 1.11E-20 | 3.79E-20 |
| **AMELX** | 4.518071389 | 1.824022738 | 3.04E-14 | 7.71E-14 |
| **KRT20** | 4.516129722 | 2.316508792 | 1.42E-15 | 3.84E-15 |
| **S100P** | 4.507919662 | 7.74141006 | 8.8E-44 | 7.84E-43 |
| **MAGEA1** | 4.505579158 | 1.94033345 | 1.1E-18 | 3.41E-18 |
| **FABP6** | 4.487929223 | 2.472089896 | 5.14E-32 | 2.87E-31 |
| **BIRC5** | 4.482263148 | 5.791400478 | 2.06E-152 | 4.01E-150 |
| **CENPA** | 4.442719719 | 4.501918495 | 1.06E-214 | 1.3E-211 |
| **ART3** | 4.438263198 | 4.841866684 | 1.02E-31 | 5.64E-31 |
| **SIM2** | 4.436996583 | 2.298897909 | 2.35E-58 | 3.7E-57 |
| **MMP12** | 4.435433636 | 5.164707948 | 6.75E-51 | 8.09E-50 |
| **HJURP** | 4.430902103 | 4.352318623 | 3.52E-251 | 5.16E-247 |
| **A2ML1** | 4.412591148 | 3.556323577 | 4.32E-63 | 8.07E-62 |
| **EN1** | 4.412228168 | 5.725427095 | 6.72E-77 | 1.94E-75 |
| **ANLN** | 4.410739219 | 5.208433816 | 1.9E-187 | 8.44E-185 |
| **S100A2** | 4.40606313 | 7.587896433 | 1.8E-39 | 1.36E-38 |
| **KIF2C** | 4.400693346 | 5.171664275 | 2.51E-222 | 3.68E-219 |
| **IGFL3** | 4.392926446 | 2.195595328 | 1.39E-29 | 6.99E-29 |
| **H2AC14** | 4.388020287 | 1.925179101 | 4.04E-27 | 1.82E-26 |
| **AMTN** | 4.382166152 | 2.877108899 | 2.33E-28 | 1.11E-27 |
| **H2BC17** | 4.375943739 | 2.565320154 | 4.21E-50 | 4.9E-49 |
| **KIF18B** | 4.371963486 | 3.782518593 | 3.15E-188 | 1.54E-185 |
| **CEP55** | 4.371174095 | 5.084747616 | 4.01E-213 | 4.51E-210 |
| **FAM83D** | 4.362079993 | 5.106252517 | 7.67E-210 | 7.02E-207 |
| **NKX2-5** | 4.34944239 | 2.627913107 | 5.27E-36 | 3.48E-35 |
| **PLK1** | 4.343548324 | 4.646981784 | 1.95E-228 | 4.08E-225 |
| **CCL7** | 4.322078458 | 2.45474566 | 4.78E-46 | 4.64E-45 |
| **KRT81** | 4.317497634 | 8.463933138 | 6.47E-35 | 4.07E-34 |
| **SPRR2D** | 4.316416654 | 2.377752331 | 1.07E-16 | 3.05E-16 |
| **IVL** | 4.314380806 | 2.272490684 | 1.99E-28 | 9.52E-28 |
| **ASPM** | 4.310221575 | 3.924730666 | 5.73E-163 | 1.37E-160 |
| **KLHDC7B** | 4.308664783 | 5.033982494 | 4.03E-52 | 5.1E-51 |
| **PAGE2B** | 4.307933597 | 2.144200687 | 8.32E-19 | 2.59E-18 |
| **PKMYT1** | 4.303140838 | 3.635314602 | 1.24E-162 | 2.93E-160 |
| **GABRQ** | 4.284656285 | 1.948255875 | 1.31E-28 | 6.31E-28 |
| **COL22A1** | 4.283093058 | 3.213277356 | 1.39E-54 | 1.92E-53 |
| **TDO2** | 4.276046374 | 2.570715176 | 5.99E-56 | 8.62E-55 |
| **SLC15A1** | 4.272704383 | 3.239743092 | 1.09E-36 | 7.36E-36 |
| **GAL** | 4.265731404 | 4.44274879 | 8.53E-75 | 2.36E-73 |
| **EXO1** | 4.262087403 | 4.117208702 | 1.97E-173 | 6.4E-171 |
| **NDC80** | 4.261390588 | 4.603154271 | 5.66E-189 | 2.96E-186 |
| **SOX11** | 4.256329885 | 3.680683102 | 9.42E-71 | 2.3E-69 |
| **C11orf86** | 4.250360129 | 2.201032871 | 1.44E-34 | 8.94E-34 |
| **TLX3** | 4.23872934 | 1.771257002 | 2.89E-34 | 1.77E-33 |
| **DLX5** | 4.2383045 | 3.734423808 | 1.47E-56 | 2.17E-55 |
| **BUB1** | 4.235322492 | 4.464688392 | 3.06E-188 | 1.54E-185 |
| **RLBP1** | 4.234565159 | 2.321991974 | 1.11E-31 | 6.14E-31 |
| **SLC1A6** | 4.234017349 | 1.92757912 | 8.44E-26 | 3.6E-25 |
| **SKA1** | 4.230162831 | 3.904392043 | 5.9E-172 | 1.8E-169 |
| **CDCA2** | 4.215352773 | 4.154736144 | 7.86E-142 | 1.24E-139 |
| **HOXC12** | 4.209562759 | 2.927993612 | 1.48E-39 | 1.12E-38 |
| **HTR3A** | 4.194938043 | 2.233564967 | 2.66E-25 | 1.11E-24 |
| **KIF20A** | 4.18103579 | 5.052997254 | 1.02E-193 | 5.99E-191 |
| **CDKN2A** | 4.18089178 | 4.928375676 | 5.85E-77 | 1.71E-75 |
| **CDCA8** | 4.176672021 | 5.660131449 | 1.97E-231 | 5.75E-228 |
| **PCSK1N** | 4.165548055 | 4.37721517 | 1.55E-48 | 1.68E-47 |
| **MCM10** | 4.154577835 | 3.87749853 | 7.32E-154 | 1.45E-151 |
| **CENPF** | 4.154133785 | 5.463495505 | 7.32E-190 | 4.05E-187 |
| **KIFC1** | 4.152700661 | 5.773766474 | 1.19E-241 | 5.8E-238 |
| **ZIC1** | 4.149298239 | 2.771063066 | 1.21E-66 | 2.58E-65 |
| **LHX2** | 4.143140898 | 1.939794818 | 1.89E-35 | 1.21E-34 |
| **KIF14** | 4.135378552 | 3.22556309 | 6.52E-144 | 1.05E-141 |
| **CDCA3** | 4.129022724 | 4.01998114 | 1.03E-181 | 3.97E-179 |
| **TROAP** | 4.121589152 | 3.887343472 | 1.03E-187 | 4.88E-185 |
| **DLGAP5** | 4.121298641 | 4.533964484 | 4.32E-183 | 1.71E-180 |
| **PIMREG** | 4.118611565 | 3.994804724 | 7.04E-152 | 1.34E-149 |
| **PTTG1** | 4.114756942 | 5.72042484 | 7.48E-190 | 4.05E-187 |
| **PSORS1C2** | 4.111875747 | 2.681467721 | 4.17E-35 | 2.65E-34 |
| **TRPM8** | 4.103594715 | 2.16802068 | 5.78E-29 | 2.83E-28 |
| **GABBR2** | 4.098254866 | 2.094578017 | 3.55E-43 | 3.09E-42 |
| **CNIH2** | 4.095115051 | 3.55976908 | 4.34E-87 | 1.75E-85 |
| **IDO1** | 4.092958512 | 5.442502648 | 6.26E-49 | 6.91E-48 |
| **DEPDC1** | 4.074640446 | 3.501439293 | 5.61E-152 | 1.08E-149 |
| **BPIFB3** | 4.069882641 | 1.74696024 | 1.74E-14 | 4.46E-14 |
| **VGF** | 4.065901951 | 1.995515381 | 3.81E-44 | 3.45E-43 |
| **CCNB2** | 4.062610279 | 5.449182903 | 4.56E-209 | 3.93E-206 |
| **KCNG1** | 4.056883058 | 3.204047762 | 4.49E-57 | 6.76E-56 |
| **CCNE1** | 4.051255454 | 4.422488309 | 1.8E-146 | 3.06E-144 |
| **CCL20** | 4.047116216 | 3.235554597 | 7.16E-49 | 7.89E-48 |
| **ULBP1** | 4.041290447 | 2.434705016 | 8.05E-68 | 1.78E-66 |
| **NPW** | 4.038799235 | 2.665985736 | 1.92E-41 | 1.57E-40 |
| **RRM2** | 4.028445511 | 4.610313014 | 1.35E-161 | 3.13E-159 |
| **CTAG2** | 4.028111827 | 2.660594875 | 1.2E-10 | 2.55E-10 |
| **SCUBE3** | 4.022420852 | 4.087915663 | 1.86E-49 | 2.1E-48 |
| **PADI3** | 4.021634294 | 2.835511133 | 5.81E-24 | 2.3E-23 |
| **CCL11** | 4.000623285 | 2.529553599 | 1.42E-60 | 2.38E-59 |
| **TTK** | 3.99243174 | 4.377185874 | 1.51E-175 | 5.14E-173 |
| **GTSE1** | 3.991808654 | 4.043164458 | 2.62E-183 | 1.07E-180 |
| **PBK** | 3.987034844 | 4.653823094 | 6.87E-156 | 1.38E-153 |
| **CAMP** | 3.98459451 | 2.773145923 | 5.97E-40 | 4.58E-39 |
| **CDCA5** | 3.98429251 | 5.063150607 | 2.74E-210 | 2.67E-207 |
| **PLPP4** | 3.980146122 | 2.514955209 | 4.47E-66 | 9.38E-65 |
| **KRT16** | 3.979101737 | 7.842882184 | 2.01E-44 | 1.84E-43 |
| **FBN2** | 3.978616474 | 2.884428842 | 2.02E-45 | 1.92E-44 |
| **CKAP2L** | 3.973612611 | 3.351804504 | 3.24E-158 | 7.09E-156 |
| **SLC7A5** | 3.961744556 | 7.165529685 | 4.45E-138 | 6.57E-136 |
| **MKI67** | 3.961349561 | 5.183126438 | 4.48E-141 | 6.97E-139 |
| **PRSS41** | 3.936379481 | 1.859543575 | 7.89E-21 | 2.72E-20 |
| **GNG4** | 3.929693678 | 3.731816187 | 1E-44 | 9.25E-44 |
| **UBE2T** | 3.929219267 | 5.750584681 | 2.77E-198 | 1.93E-195 |
| **CXCL9** | 3.912990576 | 7.160836287 | 6.57E-63 | 1.21E-61 |
| **SPIB** | 3.906215736 | 3.395693787 | 3E-34 | 1.83E-33 |
| **SKA3** | 3.890253735 | 4.075789064 | 2.45E-174 | 8.16E-172 |
| **APOBEC3B** | 3.879525467 | 4.560620394 | 1.17E-71 | 2.92E-70 |
| **TOP2A** | 3.870580367 | 6.271125238 | 1.05E-123 | 1.09E-121 |
| **CCNA2** | 3.859320671 | 5.208279821 | 3.17E-179 | 1.19E-176 |
| **GABRA3** | 3.85688862 | 1.780057568 | 1.02E-24 | 4.17E-24 |
| **PGLYRP4** | 3.848600009 | 1.835468538 | 1.81E-32 | 1.03E-31 |
| **OTX1** | 3.835305976 | 2.962706885 | 6.76E-73 | 1.75E-71 |
| **FSD1** | 3.83240682 | 2.068161465 | 1.26E-34 | 7.82E-34 |
| **SPC24** | 3.830573336 | 4.087731647 | 1.82E-167 | 4.75E-165 |
| **KRTDAP** | 3.825904164 | 3.969133635 | 9.87E-22 | 3.54E-21 |
| **H1-5** | 3.812521566 | 2.026312038 | 2.01E-28 | 9.59E-28 |
| **SLURP1** | 3.811789517 | 2.964935043 | 5.28E-36 | 3.48E-35 |
| **S100A9** | 3.809976156 | 10.39484922 | 2.7E-43 | 2.36E-42 |
| **CDC45** | 3.809240369 | 4.183977637 | 1.3E-187 | 5.94E-185 |
| **ZNF695** | 3.803196318 | 2.216461751 | 8.42E-72 | 2.11E-70 |
| **NCAPG** | 3.79204204 | 4.031838553 | 1.33E-158 | 3E-156 |
| **STRA8** | 3.785377443 | 1.781493773 | 2.26E-31 | 1.23E-30 |
| **MT1H** | 3.776390614 | 2.551119317 | 2.32E-38 | 1.67E-37 |
| **CDK1** | 3.773912177 | 5.422728208 | 4.4E-197 | 2.93E-194 |
| **GBP5** | 3.773908367 | 4.318182882 | 1.53E-64 | 3.03E-63 |
| **UHRF1** | 3.772845988 | 4.003589757 | 5.61E-137 | 7.98E-135 |
| **AURKA** | 3.772226917 | 5.066049354 | 7.97E-231 | 1.94E-227 |
| **NPTX2** | 3.770115174 | 3.539787749 | 5.27E-32 | 2.94E-31 |
| **HOXB9** | 3.765576096 | 3.008041225 | 2.93E-36 | 1.95E-35 |
| **PITX1** | 3.759765574 | 4.465244705 | 7.34E-72 | 1.85E-70 |
| **NEIL3** | 3.746242466 | 2.709638681 | 1.38E-114 | 1.21E-112 |
| **SGO1** | 3.729989576 | 2.765889149 | 7.19E-123 | 7.31E-121 |
| **NPY** | 3.715998372 | 2.166665007 | 7.22E-19 | 2.25E-18 |
| **HOXD13** | 3.710504278 | 2.152784774 | 5.68E-24 | 2.25E-23 |
| **OR2B6** | 3.710224249 | 1.887588405 | 4.28E-43 | 3.71E-42 |
| **CBX2** | 3.70167887 | 4.512467633 | 2.43E-151 | 4.5E-149 |
| **RCOR2** | 3.696125414 | 3.865628635 | 2.01E-83 | 7.25E-82 |
| **CLSPN** | 3.694709253 | 2.906932489 | 9.14E-115 | 8.06E-113 |
| **SLC6A15** | 3.694389246 | 2.623539743 | 3.16E-31 | 1.71E-30 |
| **ADAMDEC1** | 3.693350616 | 4.289148384 | 4.52E-51 | 5.47E-50 |
| **TMSB15A** | 3.691851437 | 5.35982561 | 1.11E-67 | 2.44E-66 |
| **ODAM** | 3.68610515 | 3.513767617 | 3.94E-23 | 1.5E-22 |
| **MTFR2** | 3.68049663 | 3.262538652 | 3.86E-134 | 5.14E-132 |
| **PDIA2** | 3.679102317 | 2.793890081 | 8.02E-24 | 3.15E-23 |
| **H4C5** | 3.674730912 | 2.665191684 | 1.67E-40 | 1.31E-39 |
| **LIX1** | 3.674627029 | 1.685532859 | 4.62E-19 | 1.46E-18 |
| **C4orf51** | 3.673546394 | 1.829169368 | 2.87E-23 | 1.1E-22 |
| **H1-4** | 3.670451283 | 2.945715861 | 3.2E-29 | 1.59E-28 |
| **ALKAL1** | 3.663723997 | 1.788906447 | 1.36E-17 | 4.03E-17 |
| **KREMEN2** | 3.663153238 | 2.40983928 | 1.82E-56 | 2.67E-55 |
| **LY6D** | 3.662460665 | 4.962451522 | 2.69E-26 | 1.17E-25 |
| **SOX2** | 3.643978742 | 2.388159862 | 5.26E-19 | 1.66E-18 |
| **H4C4** | 3.643088055 | 2.320785433 | 1.63E-30 | 8.58E-30 |
| **CD1A** | 3.639393591 | 2.709949244 | 1.21E-35 | 7.82E-35 |
| **IGF2BP3** | 3.625332386 | 1.796331767 | 2.28E-29 | 1.14E-28 |
| **NCCRP1** | 3.62117179 | 5.607190121 | 1.3E-38 | 9.52E-38 |
| **TK1** | 3.618662345 | 6.923459821 | 1.09E-148 | 1.95E-146 |
| **IL4I1** | 3.613857166 | 4.439437324 | 1.94E-91 | 9.18E-90 |
| **IGFBPL1** | 3.612637537 | 2.680945048 | 7.85E-42 | 6.49E-41 |
| **IL12RB2** | 3.607209818 | 3.851559544 | 2.11E-54 | 2.9E-53 |
| **DLX6** | 3.601730547 | 1.973860748 | 2.65E-34 | 1.62E-33 |
| **ASF1B** | 3.594398968 | 5.564644446 | 2.45E-184 | 1.02E-181 |
| **CALML5** | 3.593264961 | 8.604695408 | 6.88E-26 | 2.95E-25 |
| **DEPDC1B** | 3.587556187 | 3.137993521 | 3.72E-128 | 4.19E-126 |
| **CDC25C** | 3.58136472 | 2.939015018 | 3.22E-131 | 3.89E-129 |
| **CDKN3** | 3.566568828 | 4.476020865 | 8.39E-169 | 2.27E-166 |
| **E2F8** | 3.555036471 | 2.997661256 | 2.24E-96 | 1.24E-94 |
| **GTSF1** | 3.554403278 | 2.587371899 | 3.36E-25 | 1.4E-24 |
| **ELAVL2** | 3.5528212 | 2.534576447 | 1.71E-39 | 1.29E-38 |
| **PRR11** | 3.547480488 | 4.157462096 | 9.78E-173 | 3.05E-170 |
| **NCAPH** | 3.543116895 | 3.727810591 | 8.11E-169 | 2.24E-166 |
| **CTXN3** | 3.528911464 | 2.221751587 | 2.88E-10 | 5.98E-10 |
| **BUB1B** | 3.527729292 | 3.964589869 | 2.89E-156 | 5.87E-154 |
| **H4C3** | 3.526065554 | 2.169505309 | 4.35E-27 | 1.96E-26 |
| **RAD54L** | 3.50722727 | 3.05263443 | 6.66E-122 | 6.54E-120 |
| **TRIP13** | 3.498703921 | 4.88959244 | 2.63E-158 | 5.83E-156 |
| **DLL3** | 3.494712739 | 1.949653896 | 1.16E-34 | 7.23E-34 |
| **MKRN3** | 3.469660123 | 1.892595355 | 2.79E-46 | 2.72E-45 |
| **HMMR** | 3.459985321 | 3.771129029 | 2.61E-148 | 4.59E-146 |
| **CDT1** | 3.45774117 | 4.94412706 | 1.94E-132 | 2.46E-130 |
| **E2F7** | 3.449262735 | 2.572793064 | 8.7E-69 | 2E-67 |
| **LEMD1** | 3.448165639 | 3.517053519 | 5.46E-44 | 4.89E-43 |
| **H2AC13** | 3.445236154 | 2.7596023 | 3.07E-39 | 2.29E-38 |
| **HPDL** | 3.444153731 | 3.585881171 | 1.19E-73 | 3.21E-72 |
| **COMP** | 3.442704402 | 5.603353218 | 1.31E-47 | 1.37E-46 |
| **CARD18** | 3.442303784 | 1.80060292 | 7.7E-15 | 2.01E-14 |
| **PCLAF** | 3.441173011 | 2.913233745 | 9.93E-104 | 6.82E-102 |
| **CXCL17** | 3.43840484 | 6.616377868 | 1.03E-24 | 4.2E-24 |
| **KISS1** | 3.432517947 | 2.125761106 | 6.51E-29 | 3.18E-28 |
| **NOTUM** | 3.428592776 | 2.101381415 | 1.2E-29 | 6.09E-29 |
| **KIF23** | 3.424334159 | 4.284373835 | 1.92E-167 | 4.92E-165 |
| **CLPSL1** | 3.42341311 | 3.621790733 | 5.92E-40 | 4.55E-39 |
| **H2AC12** | 3.422897351 | 1.984585117 | 3.99E-34 | 2.43E-33 |
| **FAM83A** | 3.415670713 | 2.758092976 | 2.47E-24 | 9.88E-24 |
| **KRT86** | 3.412971917 | 3.929340669 | 2.39E-40 | 1.87E-39 |
| **PIF1** | 3.405494318 | 2.607389323 | 3.49E-79 | 1.1E-77 |
| **ORC6** | 3.401096397 | 3.394316003 | 1.19E-147 | 2.05E-145 |
| **VGLL1** | 3.400615104 | 6.598979173 | 5.11E-37 | 3.51E-36 |
| **HTR1D** | 3.396766043 | 1.864356239 | 5.41E-30 | 2.78E-29 |
| **LCN2** | 3.394329878 | 7.343038533 | 1.64E-30 | 8.66E-30 |
| **CCN4** | 3.387837457 | 3.909909325 | 9.23E-63 | 1.69E-61 |
| **CKS2** | 3.385715384 | 7.671145154 | 7.31E-172 | 2.18E-169 |
| **NLRP7** | 3.373713631 | 1.80586315 | 1.39E-22 | 5.17E-22 |
| **CLEC5A** | 3.371957138 | 2.64178295 | 9.13E-70 | 2.16E-68 |
| **FAM111B** | 3.368589097 | 4.655070597 | 3.75E-100 | 2.35E-98 |
| **GPRIN1** | 3.363565412 | 3.299560483 | 1.74E-97 | 9.95E-96 |
| **FBN3** | 3.363019887 | 2.45247284 | 8.5E-38 | 5.99E-37 |
| **FOXD1** | 3.360787093 | 1.852323632 | 5.58E-35 | 3.52E-34 |
| **DTL** | 3.35760103 | 4.352961253 | 1.64E-151 | 3.08E-149 |
| **OLIG1** | 3.353939764 | 1.872330985 | 5.67E-18 | 1.7E-17 |
| **E2F2** | 3.352380102 | 3.405443427 | 1.95E-111 | 1.6E-109 |
| **H2BC11** | 3.351029753 | 3.977660319 | 1.02E-65 | 2.1E-64 |
| **ZBED2** | 3.347150572 | 2.250733462 | 1.11E-39 | 8.44E-39 |
| **ARTN** | 3.347090146 | 2.836616103 | 1.18E-49 | 1.35E-48 |
| **PAX1** | 3.336928838 | 1.730122088 | 1.71E-20 | 5.78E-20 |
| **UBE2S** | 3.336477542 | 5.159097465 | 8.6E-158 | 1.82E-155 |
| **LTB** | 3.336012529 | 5.209494737 | 1.24E-43 | 1.1E-42 |
| **BPIFB1** | 3.333910476 | 4.611252132 | 6.14E-16 | 1.69E-15 |
| **MEX3A** | 3.319415624 | 5.50440875 | 1.21E-91 | 5.74E-90 |
| **CENPM** | 3.319048103 | 4.272584903 | 3.76E-137 | 5.45E-135 |
| **ERCC6L** | 3.312064954 | 3.004981073 | 1.46E-130 | 1.69E-128 |
| **RAET1L** | 3.311146145 | 2.426631364 | 1.82E-39 | 1.37E-38 |
| **DKK 1.00** | 3.310890771 | 3.987325328 | 3.95E-31 | 2.13E-30 |
| **FDCSP** | 3.310246176 | 10.28807654 | 2.42E-19 | 7.75E-19 |
| **CDH19** | 3.309015212 | 2.935752003 | 1.55E-24 | 6.26E-24 |
| **E2F1** | 3.305005304 | 5.052706002 | 7.46E-165 | 1.85E-162 |
| **TMEM171** | 3.303830099 | 2.265448446 | 8.98E-44 | 7.99E-43 |
| **SPC25** | 3.297029262 | 3.9754065 | 5.73E-169 | 1.61E-166 |
| **KIF15** | 3.295263429 | 3.564327294 | 7.52E-135 | 1.03E-132 |
| **GFRA3** | 3.289120758 | 3.627788306 | 5.31E-33 | 3.11E-32 |
| **TMEM179** | 3.288732042 | 1.774178465 | 6.3E-19 | 1.97E-18 |
| **CHST6** | 3.286865745 | 2.225979205 | 5.42E-49 | 6E-48 |
| **GJB3** | 3.28659909 | 3.965987649 | 8.18E-47 | 8.21E-46 |
| **NXPH4** | 3.28495678 | 3.975701474 | 2.16E-47 | 2.22E-46 |
| **TUBB4A** | 3.284848979 | 2.013288333 | 9.64E-25 | 3.94E-24 |
| **ORC1** | 3.280699112 | 3.693464582 | 8.8E-131 | 1.03E-128 |
| **H3C10** | 3.274480689 | 3.70028733 | 1.87E-54 | 2.58E-53 |
| **CEMIP** | 3.269678916 | 3.262308175 | 1.2E-63 | 2.29E-62 |
| **TMEM132E** | 3.267850281 | 2.010681896 | 7.12E-24 | 2.8E-23 |
| **RAD51AP1** | 3.267602845 | 4.648655338 | 1.05E-135 | 1.46E-133 |
| **H2BC13** | 3.267400893 | 1.992073236 | 8.61E-30 | 4.39E-29 |
| **PYDC1** | 3.263642718 | 3.455781463 | 9.7E-17 | 2.76E-16 |
| **CENPE** | 3.261813799 | 3.34139712 | 8.74E-123 | 8.82E-121 |
| **TFR2** | 3.256263454 | 2.148314987 | 2.11E-51 | 2.59E-50 |
| **AARD** | 3.255636466 | 6.921828592 | 1.96E-40 | 1.53E-39 |
| **SCRG1** | 3.255160815 | 3.058711948 | 1.75E-29 | 8.78E-29 |
| **SLC5A8** | 3.248875319 | 2.682615018 | 3.62E-18 | 1.1E-17 |
| **ISG15** | 3.238911599 | 8.04722734 | 2.87E-66 | 6.07E-65 |
| **RHEX** | 3.235557867 | 4.265394179 | 9.98E-23 | 3.74E-22 |
| **EDN2** | 3.235126196 | 4.265975731 | 1.02E-42 | 8.73E-42 |
| **RECQL4** | 3.230356995 | 4.407522194 | 5.48E-142 | 8.71E-140 |
| **IGSF23** | 3.227406516 | 1.726962141 | 9.42E-28 | 4.36E-27 |
| **FAM72D** | 3.222977786 | 1.802187463 | 1.69E-46 | 1.67E-45 |
| **HOXC11** | 3.222229132 | 2.95889729 | 5.56E-54 | 7.51E-53 |
| **TINCR** | 3.221945726 | 4.130609307 | 1.96E-46 | 1.93E-45 |
| **UMODL1** | 3.22098883 | 1.772268301 | 6.44E-29 | 3.14E-28 |
| **SMC1B** | 3.210871394 | 2.30435685 | 5.96E-50 | 6.87E-49 |
| **SHCBP1** | 3.198266852 | 3.285523124 | 6.18E-128 | 6.8E-126 |
| **CLDN9** | 3.193627216 | 2.490810122 | 5.39E-42 | 4.49E-41 |
| **KNL1** | 3.193047982 | 2.829279199 | 2.49E-106 | 1.79E-104 |
| **AIM2** | 3.192126294 | 3.245334634 | 9.25E-48 | 9.71E-47 |
| **S100A7A** | 3.190855133 | 2.14392572 | 2.76E-10 | 5.74E-10 |
| **MUC5B** | 3.178931306 | 4.083534604 | 3.12E-44 | 2.83E-43 |
| **BCL2A1** | 3.17803076 | 5.293856599 | 4.88E-45 | 4.57E-44 |
| **RAD51** | 3.171030028 | 3.787457001 | 2.85E-149 | 5.14E-147 |
| **SPAG5** | 3.167113597 | 4.334800106 | 4.21E-146 | 7.09E-144 |
| **CDC25A** | 3.161052949 | 3.603447103 | 2.29E-134 | 3.07E-132 |
| **KIF18A** | 3.155930534 | 3.367325563 | 5.98E-131 | 7.12E-129 |
| **H2BC7** | 3.155852124 | 2.344882741 | 1.33E-29 | 6.73E-29 |
| **SPRR2E** | 3.15350312 | 2.497460361 | 2E-10 | 4.18E-10 |
| **PGBD5** | 3.149255141 | 2.94722262 | 1.59E-47 | 1.64E-46 |
| **TMEM158** | 3.147475273 | 5.296426934 | 4.15E-61 | 7.13E-60 |
| **CCNB1** | 3.14203137 | 6.226587177 | 9.86E-227 | 1.8E-223 |
| **CAPS** | 3.1413554 | 5.478494428 | 3.66E-57 | 5.52E-56 |
| **GPR19** | 3.139946955 | 2.43280362 | 9.99E-66 | 2.06E-64 |
| **CENPW** | 3.137620015 | 6.410307279 | 1.1E-127 | 1.2E-125 |
| **ESPL1** | 3.12949326 | 3.225338559 | 2.02E-123 | 2.08E-121 |
| **GSDMC** | 3.123060442 | 3.333579591 | 1.02E-47 | 1.07E-46 |
| **PI3** | 3.120736562 | 7.758551607 | 1.57E-19 | 5.08E-19 |
| **FAM72B** | 3.118180071 | 2.088072001 | 8.89E-69 | 2.05E-67 |
| **SEZ6** | 3.118150895 | 1.883766785 | 2.75E-12 | 6.34E-12 |
| **EZH2** | 3.117846057 | 4.733399662 | 1.46E-176 | 5.21E-174 |
| **AUNIP** | 3.117658489 | 3.35953251 | 8.15E-122 | 7.95E-120 |
| **KIF11** | 3.116240907 | 4.860557481 | 5.43E-164 | 1.32E-161 |
| **TEDC2** | 3.113526752 | 3.343827449 | 2.28E-107 | 1.74E-105 |
| **EEF1AKMT4** | 3.111989022 | 4.834464534 | 1.56E-134 | 2.11E-132 |
| **GRIN2D** | 3.109842224 | 2.65367514 | 2.39E-42 | 2.01E-41 |
| **NIPAL4** | 3.107426584 | 1.918457203 | 3.53E-28 | 1.67E-27 |
| **BPI** | 3.094245725 | 2.832125449 | 2.76E-29 | 1.37E-28 |
| **SERPINB2** | 3.092759355 | 2.543708056 | 3.7E-21 | 1.29E-20 |
| **LAMP3** | 3.091996253 | 4.505922381 | 1.85E-68 | 4.22E-67 |
| **CDC6** | 3.08629735 | 4.077049846 | 1.23E-130 | 1.43E-128 |
| **FAM72A** | 3.084584875 | 1.91981495 | 4.61E-56 | 6.65E-55 |
| **MYBL1** | 3.084505154 | 3.844478687 | 1.2E-58 | 1.9E-57 |
| **GINS1** | 3.081636188 | 4.265697902 | 2.56E-165 | 6.45E-163 |
| **DIAPH3** | 3.067756634 | 3.109812168 | 9.23E-91 | 4.17E-89 |
| **INHBA** | 3.061603966 | 4.296238637 | 1.01E-95 | 5.49E-94 |
| **LMNB1** | 3.061202021 | 5.974507803 | 6.09E-158 | 1.31E-155 |
| **TNFRSF9** | 3.060328352 | 2.087217677 | 1.66E-54 | 2.3E-53 |
| **SIX2** | 3.050263656 | 4.108226574 | 2.75E-40 | 2.13E-39 |
| **FATE1** | 3.049766467 | 2.253298124 | 1.28E-17 | 3.78E-17 |
| **RMI2** | 3.048994861 | 4.104246999 | 9.35E-112 | 7.77E-110 |
| **TICRR** | 3.04352483 | 2.949169381 | 4.57E-100 | 2.86E-98 |
| **INSM1** | 3.041704618 | 2.007504217 | 4.45E-16 | 1.23E-15 |
| **NKX1-2** | 3.039258017 | 2.576408408 | 6.03E-34 | 3.65E-33 |
| **SYT13** | 3.031254838 | 3.299030867 | 1.23E-29 | 6.22E-29 |
| **IL21R** | 3.023116193 | 2.345884218 | 5.08E-49 | 5.64E-48 |
| **GNAT3** | 3.021261481 | 1.662638905 | 2.81E-10 | 5.85E-10 |
| **HES6** | 3.020868896 | 3.873901891 | 7.3E-68 | 1.63E-66 |
| **MFAP2** | 3.018814077 | 5.318280445 | 3.96E-96 | 2.18E-94 |
| **PRSS2** | 3.015987065 | 2.197819015 | 7.12E-17 | 2.04E-16 |
| **ULBP2** | 3.013795866 | 2.994204271 | 5.37E-52 | 6.77E-51 |
| **POLQ** | 3.013131184 | 2.467592444 | 4.57E-89 | 1.94E-87 |
| **ARHGAP11A** | 3.012127453 | 3.609241723 | 4.27E-137 | 6.13E-135 |
| **TACC3** | 3.012045267 | 4.601969737 | 5.69E-179 | 2.08E-176 |
| **GPC2** | 3.010603159 | 2.6750281 | 1.99E-48 | 2.15E-47 |
| **COL26A1** | 3.003029586 | 2.489077913 | 1.68E-23 | 6.51E-23 |
| **CDCA7** | 3.001673893 | 4.476689471 | 1.12E-106 | 8.17E-105 |
| **HMGB3** | 2.987950933 | 6.186729648 | 2.55E-194 | 1.56E-191 |
| **H2BC9** | 2.984387776 | 2.91208368 | 1.16E-51 | 1.44E-50 |
| **ZWINT** | 2.98382749 | 5.821334021 | 2.1E-144 | 3.41E-142 |
| **MAB21L4** | 2.978311264 | 3.6581862 | 1.9E-27 | 8.71E-27 |
| **CLPS** | 2.975532869 | 1.897518606 | 1.97E-26 | 8.65E-26 |
| **MTRNR2L8** | 2.974728848 | 3.730045728 | 2.97E-20 | 9.94E-20 |
| **CCR8** | 2.974526903 | 1.918846641 | 2.4E-43 | 2.1E-42 |
| **ZP2** | 2.973072154 | 2.029471449 | 3.88E-10 | 7.99E-10 |
| **OR2I1P** | 2.972548749 | 4.124455647 | 4.28E-36 | 2.83E-35 |
| **LAG3** | 2.96353022 | 3.498841807 | 3.64E-51 | 4.43E-50 |
| **STIL** | 2.961618087 | 3.728417192 | 2.25E-125 | 2.38E-123 |
| **LGR5** | 2.957164036 | 3.050094361 | 5.23E-19 | 1.65E-18 |
| **CRABP1** | 2.95610164 | 7.068893151 | 1.36E-23 | 5.29E-23 |
| **TAFA3** | 2.954973393 | 3.448328209 | 4.01E-36 | 2.66E-35 |
| **SPINK6** | 2.953647652 | 1.801126058 | 6.79E-14 | 1.69E-13 |
| **FZD9** | 2.951594436 | 3.234141957 | 1.63E-37 | 1.14E-36 |
| **WDR62** | 2.948274742 | 2.89899013 | 1.24E-58 | 1.96E-57 |
| **OPRK1** | 2.948216001 | 1.760705338 | 3.26E-23 | 1.25E-22 |
| **NKX3-2** | 2.942220537 | 1.888376746 | 1.03E-29 | 5.22E-29 |
| **S100A7** | 2.94002759 | 8.059894143 | 1.69E-09 | 3.38E-09 |
| **C5orf46** | 2.931999362 | 4.324164424 | 8.58E-45 | 7.95E-44 |
| **LRRC15** | 2.930710132 | 5.001501142 | 5.74E-59 | 9.18E-58 |
| **ARL 9.00** | 2.927334168 | 3.320138037 | 8.17E-57 | 1.22E-55 |
| **CD177** | 2.924910423 | 2.095627536 | 1.47E-17 | 4.33E-17 |
| **CHRDL2** | 2.909131552 | 3.280512126 | 4.47E-23 | 1.7E-22 |
| **CEL** | 2.909012515 | 4.170821919 | 3.27E-14 | 8.28E-14 |
| **LBP** | 2.907386875 | 6.069810107 | 3.38E-16 | 9.41E-16 |
| **GLDC** | 2.905544059 | 2.412581558 | 1.96E-24 | 7.88E-24 |
| **ZIC2** | 2.903846416 | 2.073420787 | 1.76E-20 | 5.95E-20 |
| **TNIP3** | 2.902229734 | 1.785939663 | 1.26E-26 | 5.58E-26 |
| **PCSK9** | 2.900886687 | 2.029277103 | 4.09E-21 | 1.43E-20 |
| **FANCA** | 2.894847937 | 2.980063494 | 5.19E-93 | 2.58E-91 |
| **SCT** | 2.893134008 | 2.031186139 | 2.22E-37 | 1.53E-36 |
| **ADAMTS14** | 2.88297384 | 2.334409579 | 3.81E-42 | 3.19E-41 |
| **JPT1** | 2.87877436 | 7.012572949 | 2.35E-131 | 2.86E-129 |
| **STMN1** | 2.877997014 | 7.312324258 | 2.81E-168 | 7.47E-166 |
| **SLC26A9** | 2.870471493 | 1.894646567 | 2.29E-17 | 6.69E-17 |
| **CENPI** | 2.870217768 | 2.968399572 | 2.3E-105 | 1.62E-103 |
| **TFF2** | 2.866805903 | 2.765505675 | 6.27E-14 | 1.57E-13 |
| **CRYBG2** | 2.864436173 | 1.790056982 | 1.93E-35 | 1.24E-34 |
| **TUNAR** | 2.861092766 | 2.447710232 | 6.64E-14 | 1.65E-13 |
| **F12** | 2.860156058 | 2.941813251 | 9.3E-53 | 1.2E-51 |
| **CRISP3** | 2.856710126 | 5.10183738 | 3.5E-09 | 6.88E-09 |
| **MAD2L1** | 2.853264315 | 4.276396146 | 3.89E-130 | 4.45E-128 |
| **CTXN1** | 2.849089211 | 5.626837669 | 1.19E-67 | 2.6E-66 |
| **CAMK2N2** | 2.848572767 | 2.447416313 | 3.7E-42 | 3.1E-41 |
| **KIF26B** | 2.846547025 | 3.131083658 | 1.01E-54 | 1.4E-53 |
| **MUC13** | 2.843729544 | 1.725576033 | 1.61E-16 | 4.55E-16 |
| **ESM1** | 2.842472327 | 3.482125357 | 1.16E-69 | 2.72E-68 |
| **KLRG2** | 2.841209004 | 4.065197722 | 1.05E-47 | 1.1E-46 |
| **C6orf15** | 2.832469889 | 4.115923807 | 1.68E-21 | 5.97E-21 |
| **SLC16A3** | 2.829167818 | 4.00559901 | 3.86E-63 | 7.22E-62 |
| **ESCO2** | 2.828309345 | 2.356276374 | 1.54E-73 | 4.13E-72 |
| **SPP1** | 2.827446807 | 8.393642751 | 1.03E-44 | 9.48E-44 |
| **CST6** | 2.827274229 | 4.349161608 | 6.29E-34 | 3.81E-33 |
| **FOXJ1** | 2.822533151 | 2.178103848 | 5.09E-19 | 1.61E-18 |
| **SHISAL1** | 2.819601561 | 2.597398755 | 1.38E-26 | 6.09E-26 |
| **RUFY4** | 2.817293618 | 1.816740462 | 1.43E-26 | 6.31E-26 |
| **HASPIN** | 2.814887934 | 2.71178387 | 1.4E-95 | 7.55E-94 |
| **C1QL4** | 2.810903073 | 2.426954461 | 3.68E-32 | 2.07E-31 |
| **CHEK1** | 2.8091561 | 4.135345695 | 4.63E-138 | 6.77E-136 |
| **MMP3** | 2.802737791 | 4.8639548 | 2.63E-30 | 1.37E-29 |
| **INAVA** | 2.801296938 | 3.812165282 | 8.96E-70 | 2.12E-68 |
| **BRSK2** | 2.796864461 | 1.787384552 | 3.45E-22 | 1.26E-21 |
| **KPNA2** | 2.792665395 | 6.321572018 | 9.63E-196 | 6.13E-193 |
| **ETV7** | 2.790899951 | 3.859373507 | 5.28E-69 | 1.22E-67 |
| **FOXI3** | 2.784489228 | 2.174495789 | 3.76E-25 | 1.56E-24 |
| **CCL17** | 2.779249476 | 3.300345587 | 5.66E-33 | 3.31E-32 |
| **SALL4** | 2.778862042 | 1.948326897 | 1.11E-37 | 7.79E-37 |
| **ADM2** | 2.777029441 | 3.964947171 | 4.8E-83 | 1.71E-81 |
| **KCNK5** | 2.775434689 | 5.130783666 | 4.78E-62 | 8.55E-61 |
| **PSAT1** | 2.77431736 | 6.183622078 | 7.2E-79 | 2.23E-77 |
| **GINS4** | 2.770868703 | 3.106044701 | 3.99E-77 | 1.17E-75 |
| **GPR158** | 2.760083406 | 1.794556226 | 2.18E-30 | 1.14E-29 |
| **KCNH2** | 2.754402533 | 2.504716196 | 8.91E-30 | 4.54E-29 |
| **PANX2** | 2.750223469 | 3.485110582 | 4.2E-50 | 4.88E-49 |
| **CENPU** | 2.7456868 | 4.637505712 | 7.14E-118 | 6.61E-116 |
| **PPP2R2C** | 2.743866819 | 2.579770682 | 6.94E-26 | 2.98E-25 |
| **GPR84** | 2.724898017 | 2.732382435 | 4.16E-55 | 5.85E-54 |
| **PSRC1** | 2.721089705 | 4.318672991 | 2.21E-126 | 2.36E-124 |
| **VSTM2L** | 2.710312909 | 3.249995483 | 1.35E-28 | 6.52E-28 |
| **FABP7** | 2.710185462 | 6.511135461 | 1.32E-17 | 3.91E-17 |
| **BLM** | 2.709924774 | 2.990715703 | 1.38E-97 | 7.96E-96 |
| **FAM133A** | 2.70924756 | 1.908923146 | 8.3E-16 | 2.27E-15 |
| **GZMB** | 2.708390056 | 4.173106733 | 6.09E-44 | 5.45E-43 |
| **CDC7** | 2.703278442 | 4.238576012 | 1.88E-106 | 1.36E-104 |
| **XAGE3** | 2.70184428 | 2.706649468 | 2.5E-08 | 4.68E-08 |
| **SPRR1A** | 2.69811596 | 3.685387354 | 5.28E-08 | 9.74E-08 |
| **EPHX3** | 2.696517454 | 3.087982385 | 3.04E-23 | 1.16E-22 |
| **CILP2** | 2.693469161 | 2.965383747 | 1.37E-50 | 1.63E-49 |
| **IFNG** | 2.69316202 | 1.996245699 | 7.49E-25 | 3.07E-24 |
| **HSD17B6** | 2.68987311 | 2.774568277 | 1.74E-72 | 4.45E-71 |
| **TMEM92** | 2.687660473 | 1.847262154 | 4.68E-27 | 2.11E-26 |
| **CCL25** | 2.687594102 | 1.877422295 | 5.2E-14 | 1.3E-13 |
| **OIP5** | 2.683504167 | 2.420423308 | 8.1E-79 | 2.51E-77 |
| **MAST1** | 2.678962318 | 1.884144988 | 2.51E-30 | 1.31E-29 |
| **CCNE2** | 2.677776213 | 2.817966077 | 3.92E-57 | 5.92E-56 |
| **HOXC13** | 2.677277589 | 3.477544365 | 2.22E-64 | 4.36E-63 |
| **TCL1A** | 2.674836227 | 2.456509693 | 2.82E-16 | 7.86E-16 |
| **HMGA1** | 2.669380746 | 8.252369315 | 3.08E-133 | 4.03E-131 |
| **SAPCD2** | 2.657989348 | 3.802884564 | 6.08E-79 | 1.9E-77 |
| **SLC22A31** | 2.655666838 | 2.012635082 | 1.79E-15 | 4.81E-15 |
| **HOXA11** | 2.65275085 | 2.315582534 | 1.28E-19 | 4.15E-19 |
| **CALHM6** | 2.651660538 | 2.482748442 | 2.53E-30 | 1.32E-29 |
| **FUT3** | 2.647697766 | 3.299009524 | 1.24E-33 | 7.45E-33 |
| **SOX8** | 2.642795442 | 4.186873999 | 2.33E-24 | 9.33E-24 |
| **MPZ** | 2.638128061 | 5.032564332 | 1.28E-16 | 3.63E-16 |
| **COCH** | 2.63038297 | 2.672424074 | 3.46E-27 | 1.57E-26 |
| **CWH43** | 2.628272948 | 2.141934818 | 2.11E-20 | 7.11E-20 |
| **MCM4** | 2.627766156 | 6.490641201 | 1.19E-131 | 1.48E-129 |
| **RSPO4** | 2.626468567 | 2.444081142 | 4.09E-29 | 2.02E-28 |
| **H2AC20** | 2.62359317 | 2.966258072 | 1.16E-34 | 7.27E-34 |
| **APLP1** | 2.621319184 | 3.671088422 | 8.15E-44 | 7.27E-43 |
| **SCX** | 2.621274828 | 2.556930099 | 5.27E-35 | 3.33E-34 |
| **MMP10** | 2.621082463 | 2.949326413 | 6.05E-16 | 1.66E-15 |
| **PRR7** | 2.620030378 | 3.424882374 | 1.47E-56 | 2.17E-55 |
| **RASD2** | 2.612371609 | 5.372619181 | 3.71E-50 | 4.32E-49 |
| **SBK1** | 2.610556054 | 4.672555985 | 3.73E-60 | 6.13E-59 |
| **PPM1E** | 2.608331447 | 1.944975645 | 3.63E-23 | 1.38E-22 |
| **TMEM132A** | 2.60531451 | 5.53252848 | 1.77E-80 | 5.77E-79 |
| **FOXP3** | 2.604753834 | 3.022982028 | 4.32E-72 | 1.09E-70 |
| **SQLE** | 2.599939656 | 6.201987499 | 5.37E-84 | 1.97E-82 |
| **PYCR1** | 2.59904588 | 6.163033485 | 5.97E-87 | 2.41E-85 |
| **CLDN10** | 2.595905806 | 3.273314406 | 3.73E-14 | 9.4E-14 |
| **NEFH** | 2.595301551 | 3.522708127 | 1.25E-21 | 4.46E-21 |
| **SH2D2A** | 2.594591627 | 3.416883839 | 9.11E-97 | 5.09E-95 |
| **TYMS** | 2.594082431 | 3.883424774 | 1.79E-79 | 5.71E-78 |
| **PAQR4** | 2.592970439 | 4.046319348 | 6.73E-121 | 6.44E-119 |
| **RNFT2** | 2.590520943 | 2.461411275 | 8.88E-56 | 1.27E-54 |
| **H2AX** | 2.590318558 | 6.617331497 | 1.22E-121 | 1.19E-119 |
| **TIGIT** | 2.589887205 | 2.721257588 | 3.69E-47 | 3.75E-46 |
| **H3C8** | 2.589667094 | 1.835581213 | 4.32E-23 | 1.64E-22 |
| **OGDHL** | 2.584766056 | 1.805053762 | 2.01E-20 | 6.77E-20 |
| **IFI6** | 2.579771956 | 9.743579975 | 9.87E-44 | 8.75E-43 |
| **TONSL** | 2.579127365 | 3.79796849 | 1.56E-112 | 1.31E-110 |
| **FN1** | 2.578584948 | 9.083002751 | 2.03E-57 | 3.1E-56 |
| **SLC6A11** | 2.569236867 | 2.544345233 | 1.65E-46 | 1.63E-45 |
| **RIPPLY3** | 2.568494889 | 2.333606739 | 3.99E-38 | 2.85E-37 |
| **CNMD** | 2.56813575 | 1.838814348 | 9.72E-10 | 1.97E-09 |
| **RDM1** | 2.567332927 | 1.783107734 | 5.07E-37 | 3.48E-36 |
| **XRCC2** | 2.56473549 | 3.188150094 | 9.73E-93 | 4.79E-91 |
| **CIP2A** | 2.557330796 | 3.630632236 | 7.29E-110 | 5.8E-108 |
| **LGALS7B** | 2.557066403 | 3.993991112 | 6.59E-15 | 1.72E-14 |
| **B3GNT7** | 2.548686915 | 4.304969486 | 1.85E-38 | 1.33E-37 |
| **PRSS27** | 2.546867396 | 2.613366096 | 1.96E-39 | 1.48E-38 |
| **PDCD1** | 2.546302563 | 2.69941238 | 3.17E-35 | 2.02E-34 |
| **GJB6** | 2.545431649 | 2.246530559 | 5.81E-16 | 1.6E-15 |
| **GSTA2** | 2.541596808 | 1.779123797 | 5.89E-17 | 1.7E-16 |
| **SLC2A6** | 2.539626423 | 3.468253338 | 2.71E-55 | 3.84E-54 |
| **MLLT11** | 2.538649307 | 4.094121054 | 2.14E-49 | 2.41E-48 |
| **NFE2L3** | 2.533617595 | 4.661008408 | 1E-92 | 4.92E-91 |
| **MYCN** | 2.533403293 | 2.744118851 | 1.27E-31 | 7E-31 |
| **EME1** | 2.530955981 | 2.359083075 | 1.46E-68 | 3.34E-67 |
| **SNX22** | 2.528278372 | 3.088203321 | 1.26E-32 | 7.23E-32 |
| **RPRM** | 2.52522781 | 2.072339355 | 2.65E-19 | 8.46E-19 |
| **HLA-G** | 2.522316681 | 4.043778681 | 6.61E-28 | 3.09E-27 |
| **SLC6A17** | 2.522126717 | 2.173321372 | 1.57E-28 | 7.55E-28 |
| **LIPG** | 2.520887903 | 2.732150925 | 3.35E-24 | 1.34E-23 |
| **ATAD2** | 2.519614575 | 5.527216895 | 3.75E-108 | 2.9E-106 |
| **SLC7A11** | 2.519521906 | 2.46497116 | 1.62E-40 | 1.28E-39 |
| **CA6** | 2.519437843 | 3.713670045 | 1.76E-11 | 3.89E-11 |
| **CARMIL2** | 2.518979176 | 2.41767296 | 4.86E-38 | 3.45E-37 |
| **PGC** | 2.518518619 | 3.256109301 | 1.34E-06 | 2.28E-06 |
| **LRP8** | 2.516505207 | 2.309581202 | 5.58E-59 | 8.93E-58 |
| **PACSIN1** | 2.512512383 | 2.18105824 | 1.22E-35 | 7.87E-35 |
| **OASL** | 2.507177005 | 4.156783903 | 2.17E-47 | 2.23E-46 |
| **CYP27C1** | 2.503567016 | 2.267283265 | 1.08E-26 | 4.81E-26 |
| **CTHRC1** | 2.50202186 | 7.095808831 | 5.79E-66 | 1.2E-64 |
| **C2CD4A** | 2.501420025 | 2.745279991 | 2.86E-12 | 6.57E-12 |
| **CSN3** | 2.500735626 | 7.72084882 | 6.51E-06 | 1.06E-05 |
| **HAGHL** | 2.494077869 | 2.378894351 | 1.08E-43 | 9.53E-43 |
| **BAIAP2L2** | 2.493986652 | 3.059988264 | 8.93E-39 | 6.57E-38 |
| **RTKN2** | 2.491448584 | 2.581011883 | 1.82E-67 | 3.97E-66 |
| **GGH** | 2.488887949 | 4.041903738 | 5.53E-92 | 2.65E-90 |
| **TLCD1** | 2.478690809 | 4.608270459 | 3.7E-69 | 8.64E-68 |
| **CTSV** | 2.474465471 | 4.090966035 | 2.38E-42 | 2.01E-41 |
| **DIPK1C** | 2.473069662 | 2.155127646 | 5.19E-16 | 1.43E-15 |
| **LIMD2** | 2.469965257 | 4.358571081 | 7.73E-71 | 1.89E-69 |
| **ICOS** | 2.461103357 | 2.483122829 | 6.41E-38 | 4.53E-37 |
| **ADAM8** | 2.455289723 | 3.986458743 | 1.86E-74 | 5.12E-73 |
| **AMH** | 2.448283013 | 2.024402037 | 1.28E-22 | 4.78E-22 |
| **BGN** | 2.446606051 | 9.671559092 | 3.59E-60 | 5.92E-59 |
| **BRIP1** | 2.445976595 | 2.590538852 | 6.89E-63 | 1.27E-61 |
| **SEPTIN3** | 2.445930753 | 4.167667018 | 7.51E-43 | 6.46E-42 |
| **CD19** | 2.442843592 | 2.506730013 | 2.18E-17 | 6.38E-17 |
| **FUT7** | 2.441280891 | 1.845594842 | 2.66E-27 | 1.21E-26 |
| **CHAC1** | 2.437447424 | 3.248067065 | 1.84E-59 | 2.98E-58 |
| **SULT4A1** | 2.435055804 | 2.292041431 | 1.08E-30 | 5.74E-30 |
| **TNFRSF18** | 2.434205102 | 3.905503207 | 2.34E-43 | 2.04E-42 |
| **PLAUR** | 2.433770017 | 5.190400238 | 1.5E-90 | 6.72E-89 |
| **MCM2** | 2.431191157 | 4.958207229 | 2.36E-81 | 7.91E-80 |
| **BOP 1.00** | 2.426795295 | 6.266507593 | 3.96E-98 | 2.37E-96 |
| **H2BC12** | 2.426348778 | 7.704511616 | 3.7E-71 | 9.14E-70 |
| **SHC4** | 2.423827347 | 3.985680686 | 3.35E-25 | 1.39E-24 |
| **DBNDD1** | 2.4187448 | 5.18759819 | 7.73E-61 | 1.32E-59 |
| **CPA4** | 2.416819168 | 3.435059945 | 3.89E-34 | 2.37E-33 |
| **MAP7D2** | 2.415033597 | 2.450805097 | 1.29E-25 | 5.43E-25 |
| **H4C8** | 2.407476531 | 4.750446908 | 4.54E-28 | 2.13E-27 |
| **DSCC1** | 2.406304804 | 4.476357422 | 9.42E-112 | 7.79E-110 |
| **CIT** | 2.406125173 | 2.339780652 | 1.98E-66 | 4.2E-65 |
| **PPP1R14B** | 2.403673722 | 7.568030993 | 1.21E-108 | 9.5E-107 |
| **CRLF1** | 2.400361509 | 4.533880897 | 1.12E-18 | 3.46E-18 |
| **CXCL5** | 2.395805675 | 3.413989439 | 1.89E-16 | 5.3E-16 |
| **DNER** | 2.395531517 | 3.026538058 | 1.7E-20 | 5.77E-20 |
| **LAD1** | 2.393363464 | 6.147087291 | 2.89E-54 | 3.95E-53 |
| **PCSK1** | 2.390405183 | 2.020279074 | 4.87E-18 | 1.47E-17 |
| **TMCC2** | 2.388321456 | 3.088143201 | 6.84E-40 | 5.23E-39 |
| **KLK6** | 2.388187806 | 5.643295343 | 5.88E-19 | 1.85E-18 |
| **SLC39A4** | 2.380802623 | 4.204503855 | 1.29E-65 | 2.64E-64 |
| **CLEC2L** | 2.378552289 | 1.975638874 | 1.3E-15 | 3.5E-15 |
| **POC1A** | 2.377135172 | 4.227218332 | 4.56E-124 | 4.77E-122 |
| **BMP8A** | 2.374110035 | 2.653294692 | 6.89E-54 | 9.26E-53 |
| **BRINP3** | 2.372078577 | 2.503248717 | 1.47E-09 | 2.94E-09 |
| **KRT83** | 2.371463645 | 2.444575449 | 1.93E-21 | 6.84E-21 |
| **DRAXIN** | 2.370669757 | 1.941257139 | 2.15E-26 | 9.42E-26 |
| **MRPL12** | 2.370617456 | 4.364515626 | 3.63E-39 | 2.7E-38 |
| **SLC52A2** | 2.370359126 | 5.979977279 | 1.65E-117 | 1.52E-115 |
| **C2CD4D** | 2.369862275 | 3.004815881 | 4.34E-51 | 5.26E-50 |
| **TRIM59** | 2.363881384 | 2.93282988 | 6.58E-91 | 2.99E-89 |
| **TDRD12** | 2.360792638 | 2.586155051 | 1.86E-10 | 3.89E-10 |
| **KRT4** | 2.360410579 | 3.807513344 | 1.74E-07 | 3.13E-07 |
| **DUSP9** | 2.359018095 | 2.727406319 | 1.02E-28 | 4.93E-28 |
| **PLAAT1** | 2.358868417 | 3.982210001 | 1.58E-53 | 2.1E-52 |
| **LTA** | 2.358515212 | 2.204675655 | 2.56E-34 | 1.57E-33 |
| **ABRACL** | 2.357436892 | 7.468696477 | 1.14E-95 | 6.18E-94 |
| **SH3GL2** | 2.354578453 | 1.813855363 | 4.22E-16 | 1.17E-15 |
| **CCDC167** | 2.351868234 | 6.629910799 | 1.72E-140 | 2.63E-138 |
| **ECEL1** | 2.347174495 | 3.574183163 | 1.95E-14 | 4.99E-14 |
| **APBA2** | 2.341071589 | 3.211309365 | 1.39E-53 | 1.85E-52 |
| **OLAH** | 2.339224251 | 2.291654718 | 7.15E-14 | 1.78E-13 |
| **FANCI** | 2.337534438 | 3.774040943 | 8.65E-100 | 5.38E-98 |
| **EXTL1** | 2.336514422 | 3.844297851 | 4.76E-16 | 1.31E-15 |
| **GINS2** | 2.333900152 | 4.380842467 | 1.89E-88 | 7.89E-87 |
| **MESP2** | 2.332665624 | 2.980902955 | 3.09E-28 | 1.46E-27 |
| **LSR** | 2.329581705 | 7.735720885 | 7.81E-81 | 2.56E-79 |
| **CD80** | 2.327866171 | 1.798265014 | 3.01E-34 | 1.84E-33 |
| **ECT2** | 2.327316702 | 5.228264872 | 1.87E-115 | 1.67E-113 |
| **H4C9** | 2.326877264 | 5.211150162 | 4.26E-73 | 1.12E-71 |
| **CDH2** | 2.326767279 | 2.41391693 | 9.71E-23 | 3.64E-22 |
| **PLEK2** | 2.325637226 | 4.740612144 | 7.56E-33 | 4.39E-32 |
| **PLK4** | 2.320246812 | 3.572044296 | 1.17E-97 | 6.81E-96 |
| **UPK2** | 2.319935882 | 2.150297355 | 3.03E-26 | 1.32E-25 |
| **CELF4** | 2.319123763 | 1.775198179 | 1.01E-21 | 3.62E-21 |
| **RACGAP1** | 2.315754745 | 5.201360718 | 1.89E-157 | 3.96E-155 |
| **QPCT** | 2.314324006 | 4.910875704 | 5.91E-40 | 4.54E-39 |
| **IGFL2** | 2.314233076 | 1.790155728 | 6.08E-23 | 2.3E-22 |
| **IGFL1** | 2.313850147 | 2.418817664 | 3.1E-14 | 7.86E-14 |
| **BCL2L14** | 2.313497517 | 2.605356275 | 9.21E-24 | 3.61E-23 |
| **MUC16** | 2.313306906 | 2.858585466 | 3.48E-20 | 1.16E-19 |
| **ARHGEF39** | 2.312976408 | 2.410468711 | 7.35E-70 | 1.74E-68 |
| **EFNA3** | 2.312213874 | 4.486973559 | 1.72E-48 | 1.87E-47 |
| **CTPS1** | 2.309554706 | 4.22817166 | 1.09E-124 | 1.15E-122 |
| **LYPD5** | 2.307992512 | 3.37364267 | 8.65E-36 | 5.64E-35 |
| **CHRM3** | 2.306184691 | 2.459053043 | 8.36E-26 | 3.58E-25 |
| **DNA2** | 2.305176194 | 3.308306865 | 6.08E-77 | 1.77E-75 |
| **RASGRF1** | 2.303855407 | 2.069495119 | 1.73E-25 | 7.24E-25 |
| **P2RY6** | 2.301907267 | 3.210328589 | 4.23E-50 | 4.91E-49 |
| **SERPINE2** | 2.299155032 | 5.23785912 | 2.61E-52 | 3.32E-51 |
| **ADAM19** | 2.298167961 | 3.799535938 | 1E-63 | 1.92E-62 |
| **DDIAS** | 2.297532661 | 3.081892268 | 4.1E-82 | 1.41E-80 |
| **PPP1R14C** | 2.297432554 | 5.378827424 | 6.42E-31 | 3.44E-30 |
| **LRRC14B** | 2.295297134 | 2.532524657 | 6.11E-10 | 1.25E-09 |
| **MYO3B** | 2.29477114 | 1.753125065 | 1.58E-21 | 5.61E-21 |
| **DNMT3B** | 2.28954564 | 3.481005385 | 4E-69 | 9.32E-68 |
| **TAP1** | 2.28278816 | 6.607776576 | 7.06E-62 | 1.26E-60 |
| **SDC1** | 2.280338496 | 8.428558353 | 9.96E-43 | 8.53E-42 |
| **CHI3L2** | 2.27940491 | 6.590150926 | 5.38E-30 | 2.77E-29 |
| **RDH10** | 2.277877547 | 6.210515665 | 2.28E-36 | 1.52E-35 |
| **FEN1** | 2.277381518 | 6.144282254 | 6.31E-159 | 1.44E-156 |
| **PNMA3** | 2.276660149 | 2.258429792 | 1.2E-26 | 5.32E-26 |
| **HHIPL2** | 2.272706704 | 1.874330859 | 2.4E-18 | 7.34E-18 |
| **CLEC7A** | 2.268563778 | 4.093669249 | 2.03E-51 | 2.5E-50 |
| **B4GALNT1** | 2.267406397 | 2.304901186 | 5.77E-32 | 3.22E-31 |
| **IQANK1** | 2.263950734 | 5.026756095 | 5.32E-75 | 1.48E-73 |
| **KRT9** | 2.263061457 | 1.953514353 | 1.77E-18 | 5.45E-18 |
| **CHRNA5** | 2.260649 | 1.9924054 | 2.57E-39 | 1.92E-38 |
| **SLAMF8** | 2.258240836 | 2.873014113 | 3.57E-43 | 3.1E-42 |
| **L1CAM** | 2.256853241 | 3.365277719 | 1.5E-22 | 5.59E-22 |
| **SLC2A1** | 2.253378559 | 6.258702788 | 6.8E-60 | 1.11E-58 |
| **MMP20** | 2.25292921 | 2.038641573 | 1.95E-15 | 5.22E-15 |
| **ERMN** | 2.244699113 | 1.757043956 | 1.39E-20 | 4.75E-20 |
| **KRT6B** | 2.243847057 | 7.902677914 | 1.07E-16 | 3.05E-16 |
| **MAPK15** | 2.242103599 | 3.438568759 | 8.9E-32 | 4.93E-31 |
| **PAFAH1B3** | 2.24134572 | 6.119747134 | 2.85E-81 | 9.53E-80 |
| **DHDH** | 2.235241629 | 2.664550772 | 1.39E-33 | 8.29E-33 |
| **H2AC11** | 2.233098285 | 2.859535453 | 1.76E-25 | 7.35E-25 |
| **QRFPR** | 2.232985156 | 1.887681521 | 6.75E-14 | 1.68E-13 |
| **WNT6** | 2.23174069 | 3.647625136 | 3.75E-26 | 1.63E-25 |
| **SECTM1** | 2.229376316 | 5.695408662 | 1.15E-40 | 9.09E-40 |
| **CHI3L1** | 2.228337033 | 8.218833515 | 1.92E-23 | 7.41E-23 |
| **ATP6V1C2** | 2.228291383 | 3.303455133 | 8.42E-37 | 5.73E-36 |
| **CRACD** | 2.224370481 | 2.115072798 | 2.61E-27 | 1.19E-26 |
| **CD1B** | 2.224169436 | 2.028218182 | 3.64E-18 | 1.1E-17 |
| **KLK14** | 2.222872429 | 4.589506213 | 2.74E-13 | 6.63E-13 |
| **CENPN** | 2.219644707 | 3.482986834 | 3.97E-95 | 2.09E-93 |
| **BLACAT1** | 2.21565398 | 2.206647304 | 2.58E-29 | 1.28E-28 |
| **LMNB2** | 2.214391137 | 5.990398416 | 5.15E-118 | 4.8E-116 |
| **IGLON5** | 2.213269881 | 1.818768608 | 3.55E-14 | 8.97E-14 |
| **SLC38A5** | 2.212983616 | 3.318654121 | 3.76E-39 | 2.79E-38 |
| **SLCO5A1** | 2.211923457 | 1.759384572 | 3.56E-13 | 8.56E-13 |
| **NUP210** | 2.207837343 | 5.69625873 | 1.86E-61 | 3.25E-60 |
| **SPRR2A** | 2.205154087 | 2.597218016 | 7.3E-06 | 1.19E-05 |
| **HSPA6** | 2.202782092 | 4.025471703 | 1.13E-25 | 4.79E-25 |
| **C1QL1** | 2.201996259 | 2.936151975 | 5.48E-17 | 1.58E-16 |
| **S100A14** | 2.198041436 | 8.251632703 | 2.86E-24 | 1.14E-23 |
| **VNN 1.00** | 2.19718329 | 3.271389154 | 5.34E-17 | 1.54E-16 |
| **LAGE3** | 2.19574172 | 6.508491994 | 5.45E-103 | 3.68E-101 |
| **UCHL1** | 2.194393797 | 6.340925337 | 5.61E-21 | 1.95E-20 |
| **SYNGR3** | 2.192936728 | 1.832791795 | 9.73E-30 | 4.94E-29 |
| **SMC4** | 2.192483436 | 4.998943378 | 5.66E-91 | 2.58E-89 |
| **ASPHD1** | 2.191145386 | 3.293416557 | 1.88E-27 | 8.6E-27 |
| **C4orf48** | 2.188576384 | 4.314164263 | 2.76E-38 | 1.98E-37 |
| **SIRPG** | 2.187230862 | 2.732482846 | 6.36E-32 | 3.54E-31 |
| **RAD54B** | 2.186565238 | 1.998175141 | 5.39E-44 | 4.84E-43 |
| **GPSM2** | 2.180688352 | 2.846957107 | 4.71E-68 | 1.06E-66 |
| **RPL39L** | 2.179919329 | 6.048755827 | 3.18E-103 | 2.15E-101 |
| **LYZ** | 2.176767118 | 8.150239781 | 1.03E-25 | 4.39E-25 |
| **GRHL3** | 2.171536938 | 3.028932604 | 5.5E-34 | 3.33E-33 |
| **AQP5** | 2.16938906 | 6.268442042 | 2.36E-14 | 6.01E-14 |
| **GALNT14** | 2.168204984 | 3.636416679 | 1.26E-36 | 8.51E-36 |
| **LRFN4** | 2.16720044 | 4.334280481 | 4.29E-56 | 6.2E-55 |
| **LRFN1** | 2.165957254 | 3.364269223 | 2.29E-49 | 2.58E-48 |
| **FAM131C** | 2.165355362 | 1.938295736 | 9.29E-26 | 3.95E-25 |
| **TYMP** | 2.164961036 | 6.28793726 | 1.05E-54 | 1.45E-53 |
| **DLX4** | 2.164320085 | 1.925064651 | 3.17E-27 | 1.44E-26 |
| **FAM171A2** | 2.163852946 | 2.825610928 | 1.67E-34 | 1.03E-33 |
| **ANKRD22** | 2.163563857 | 3.171430734 | 1.59E-38 | 1.16E-37 |
| **FOXC2** | 2.162734059 | 3.270833607 | 2.96E-23 | 1.13E-22 |
| **DPEP1** | 2.162451182 | 1.815730636 | 1.25E-19 | 4.05E-19 |
| **PNLDC1** | 2.160494884 | 2.586380037 | 9.2E-12 | 2.06E-11 |
| **PCP4** | 2.159765571 | 2.851338983 | 1.34E-14 | 3.46E-14 |
| **CHAF1B** | 2.158536295 | 4.295738718 | 9.94E-100 | 6.14E-98 |
| **PRSS3** | 2.152347671 | 1.99077826 | 4.2E-18 | 1.27E-17 |
| **FSCN1** | 2.151068145 | 7.272787169 | 1.72E-55 | 2.44E-54 |
| **ACBD7** | 2.150451453 | 3.345725531 | 1.15E-30 | 6.09E-30 |
| **ZNF367** | 2.150407692 | 3.649131818 | 1.22E-73 | 3.28E-72 |
| **MORN3** | 2.148981211 | 2.614180982 | 3.08E-30 | 1.6E-29 |
| **LINGO1** | 2.143440889 | 3.47094079 | 7.95E-30 | 4.06E-29 |
| **GAS2L3** | 2.142146866 | 3.020714133 | 4.38E-70 | 1.05E-68 |
| **DONSON** | 2.140677581 | 4.328649242 | 4.13E-113 | 3.54E-111 |
| **FOXL1** | 2.140284691 | 2.429555434 | 1.57E-32 | 8.97E-32 |
| **HELLS** | 2.137622972 | 3.057715267 | 6.66E-68 | 1.49E-66 |
| **EIF4EBP1** | 2.136777244 | 8.06247203 | 2.2E-57 | 3.35E-56 |
| **F5** | 2.136008731 | 2.18494731 | 2.77E-18 | 8.44E-18 |
| **CDC25B** | 2.135318579 | 6.209815268 | 2.94E-89 | 1.27E-87 |
| **ZNF469** | 2.134769436 | 3.479656781 | 1.67E-45 | 1.59E-44 |
| **PODXL2** | 2.133792378 | 6.302293937 | 1.12E-60 | 1.89E-59 |
| **CXCL8** | 2.132250053 | 4.307905429 | 9.19E-21 | 3.16E-20 |
| **VPREB3** | 2.131570784 | 3.189332323 | 2.89E-21 | 1.02E-20 |
| **ASCL2** | 2.128303962 | 3.149681915 | 6.07E-22 | 2.2E-21 |
| **SFN** | 2.127985954 | 8.11783879 | 1.51E-21 | 5.36E-21 |
| **IGSF9** | 2.125682858 | 4.675467552 | 6.41E-54 | 8.63E-53 |
| **RFC4** | 2.125015221 | 5.261651172 | 9.77E-123 | 9.79E-121 |
| **SLC35F2** | 2.123833888 | 4.933862162 | 2.96E-75 | 8.3E-74 |
| **CR2** | 2.12152 | 2.804907053 | 3.04E-13 | 7.33E-13 |
| **DDX39A** | 2.118036772 | 6.052027218 | 7.55E-135 | 1.03E-132 |
| **SEMA7A** | 2.110781392 | 3.758622705 | 4.66E-57 | 6.99E-56 |
| **FAM166C** | 2.110537647 | 2.254172291 | 1.28E-26 | 5.66E-26 |
| **H2AW** | 2.109566483 | 5.766368127 | 1.3E-43 | 1.15E-42 |
| **ZBP1** | 2.109510432 | 2.326857158 | 1.58E-29 | 7.9E-29 |
| **CD207** | 2.107694084 | 3.337073922 | 8.83E-23 | 3.32E-22 |
| **TMPRSS4** | 2.10531813 | 1.851936057 | 3.68E-11 | 8.01E-11 |
| **MGAT5B** | 2.103811345 | 1.821025485 | 7.35E-17 | 2.11E-16 |
| **RETN** | 2.101150936 | 1.883313522 | 2.14E-15 | 5.74E-15 |
| **SEMA5B** | 2.100912596 | 2.056221662 | 7.31E-22 | 2.64E-21 |
| **EPCAM** | 2.097972639 | 8.633656255 | 2.24E-38 | 1.62E-37 |
| **RHCG** | 2.095724871 | 2.918927416 | 9.22E-20 | 3.01E-19 |
| **PAEP** | 2.093917279 | 2.264928269 | 6.65E-08 | 1.22E-07 |
| **PACC1** | 2.091918132 | 4.435661073 | 5.03E-123 | 5.15E-121 |
| **TMEM45A** | 2.091115637 | 4.850780425 | 2.57E-42 | 2.16E-41 |
| **SCEL** | 2.088783982 | 2.13360625 | 7.35E-12 | 1.65E-11 |
| **NME1** | 2.088291159 | 4.522950094 | 1.61E-71 | 4.01E-70 |
| **PHLDA2** | 2.087418067 | 5.308062175 | 2.54E-34 | 1.56E-33 |
| **UPK1A** | 2.082189945 | 2.118164957 | 5.25E-13 | 1.25E-12 |
| **NKX6-1** | 2.079922558 | 1.818146302 | 6.36E-20 | 2.09E-19 |
| **ANP32E** | 2.07919735 | 7.055803233 | 5.14E-90 | 2.25E-88 |
| **LY6K** | 2.078571731 | 2.639153958 | 2.07E-20 | 6.96E-20 |
| **SDS** | 2.072966015 | 3.473522862 | 2.17E-33 | 1.29E-32 |
| **PFDN2** | 2.072519933 | 8.483783261 | 1.18E-82 | 4.15E-81 |
| **FNDC10** | 2.070924431 | 4.891189732 | 2.86E-41 | 2.32E-40 |
| **DEPDC7** | 2.070091161 | 2.300976624 | 1.72E-36 | 1.15E-35 |
| **CENPO** | 2.068756236 | 3.291424767 | 7.1E-97 | 3.98E-95 |
| **MAPK8IP2** | 2.068364909 | 3.554389447 | 1.5E-28 | 7.18E-28 |
| **UNC13D** | 2.066401089 | 4.326610247 | 7.43E-48 | 7.84E-47 |
| **CPNE7** | 2.062505898 | 3.022374965 | 1.11E-25 | 4.69E-25 |
| **H2BU1** | 2.060915822 | 1.94120976 | 6.1E-30 | 3.13E-29 |
| **CXCR3** | 2.056930256 | 3.45588346 | 3.32E-32 | 1.87E-31 |
| **OLR1** | 2.056372295 | 4.14571056 | 1.94E-34 | 1.19E-33 |
| **TCF19** | 2.056162898 | 5.453122732 | 2.5E-95 | 1.33E-93 |
| **TMEM79** | 2.053982871 | 4.996856537 | 4.9E-58 | 7.58E-57 |
| **TTYH3** | 2.052663998 | 6.295856442 | 2.58E-84 | 9.52E-83 |
| **PLA2G2D** | 2.051581236 | 3.402515835 | 1.63E-13 | 3.98E-13 |
| **P2RX5** | 2.051025223 | 1.8656802 | 1.74E-18 | 5.33E-18 |
| **OBP2B** | 2.050343987 | 4.505951887 | 7.87E-13 | 1.86E-12 |
| **CCNF** | 2.05022145 | 3.894501017 | 4.18E-98 | 2.49E-96 |
| **IL1A** | 2.047975862 | 1.799111761 | 1E-13 | 2.48E-13 |
| **P2RX2** | 2.046870088 | 1.724425434 | 4.77E-11 | 1.03E-10 |
| **FOXQ1** | 2.046009421 | 2.903980123 | 4.84E-20 | 1.6E-19 |
| **FCRL3** | 2.045886278 | 1.909960267 | 3.73E-16 | 1.04E-15 |
| **DLGAP3** | 2.044857461 | 1.711089113 | 6.99E-18 | 2.09E-17 |
| **FBXL6** | 2.04281605 | 4.273762686 | 1.9E-77 | 5.64E-76 |
| **SLC6A9** | 2.041803545 | 4.433671471 | 6.83E-61 | 1.17E-59 |
| **CNTNAP2** | 2.037261795 | 2.005141861 | 3.07E-12 | 7.05E-12 |
| **RTN4R** | 2.034559939 | 3.266082184 | 4.19E-52 | 5.29E-51 |
| **TNFSF13B** | 2.03268207 | 4.686815422 | 4.9E-34 | 2.98E-33 |
| **H2AZ1** | 2.031104391 | 7.878995696 | 5.22E-132 | 6.52E-130 |
| **MROH6** | 2.030956747 | 3.370403312 | 1.58E-39 | 1.2E-38 |
| **CELSR3** | 2.030774986 | 2.16092961 | 5.35E-29 | 2.63E-28 |
| **AOC1** | 2.027996165 | 1.736433482 | 6.73E-21 | 2.33E-20 |
| **CENPL** | 2.027348841 | 3.524184217 | 6.31E-97 | 3.55E-95 |
| **CCNA1** | 2.02712872 | 2.63315415 | 8.43E-20 | 2.76E-19 |
| **ERP27** | 2.027104107 | 5.332177534 | 2.01E-27 | 9.2E-27 |
| **CENPK** | 2.026965037 | 2.614287156 | 4.62E-60 | 7.58E-59 |
| **H2BC18** | 2.026863206 | 2.047342889 | 7.67E-20 | 2.51E-19 |
| **VAX2** | 2.025819593 | 2.582154267 | 9.5E-31 | 5.05E-30 |
| **IL11** | 2.025135599 | 1.91359813 | 7.21E-21 | 2.49E-20 |
| **CLPSL2** | 2.024521344 | 2.264785383 | 2.32E-17 | 6.78E-17 |
| **OSM** | 2.022017375 | 2.855138877 | 1.17E-29 | 5.91E-29 |
| **CD300E** | 2.021947844 | 2.495636581 | 3.96E-27 | 1.78E-26 |
| **NUAK2** | 2.021844748 | 3.254979767 | 1.11E-45 | 1.06E-44 |
| **NRTN** | 2.018642733 | 3.639634779 | 8.46E-27 | 3.78E-26 |
| **SYNDIG1** | 2.018363671 | 2.710464649 | 3.39E-27 | 1.54E-26 |
| **ALYREF** | 2.018021041 | 7.245300044 | 8.84E-103 | 5.94E-101 |
| **CHODL** | 2.01632291 | 3.229924892 | 1.49E-28 | 7.14E-28 |
| **CHST4** | 2.015105697 | 2.175949869 | 3.32E-19 | 1.06E-18 |
| **BATF2** | 2.014439773 | 3.923217851 | 1.56E-29 | 7.84E-29 |
| **CHTF18** | 2.012679047 | 3.707093967 | 2.59E-57 | 3.93E-56 |
| **KLK10** | 2.011438268 | 4.376703475 | 1.08E-15 | 2.93E-15 |
| **MTHFD2** | 2.0107062 | 4.770009695 | 2.51E-106 | 1.79E-104 |
| **MELTF** | 2.008935738 | 4.964076608 | 6.4E-34 | 3.87E-33 |
| **IL1R2** | 2.007658081 | 2.455945149 | 6.36E-21 | 2.2E-20 |
| **IL32** | 2.006220547 | 6.15381138 | 1.27E-38 | 9.33E-38 |
| **HAPLN3** | 2.006203183 | 6.191958116 | 2.42E-37 | 1.67E-36 |
| **PRR19** | 2.006111609 | 2.413805865 | 5.91E-48 | 6.27E-47 |
| **H2BC8** | 2.004680517 | 4.262436418 | 2.33E-21 | 8.21E-21 |
| **RELT** | 2.004492785 | 2.451296045 | 4.06E-58 | 6.32E-57 |
| **C21orf58** | 2.003221176 | 3.030580474 | 1.38E-69 | 3.24E-68 |
| **PARPBP** | 2.002310045 | 2.612745209 | 8.08E-62 | 1.44E-60 |
| **MMP7** | 2.002103689 | 8.667132972 | 6.68E-17 | 1.92E-16 |
| **KANK3** | -2.006797079 | 3.123742057 | 1.87E-40 | 1.47E-39 |
| **GREM2** | -2.009420363 | 2.636179916 | 1.26E-21 | 4.5E-21 |
| **MSS51** | -2.010166123 | 2.816660943 | 6.77E-18 | 2.02E-17 |
| **CCBE1** | -2.014228529 | 2.113452363 | 8.56E-34 | 5.16E-33 |
| **CD302** | -2.01485348 | 3.324118505 | 1.75E-60 | 2.92E-59 |
| **ADCY4** | -2.015343821 | 3.591158432 | 1.17E-67 | 2.56E-66 |
| **NR4A1** | -2.01651576 | 6.293946171 | 1.67E-27 | 7.66E-27 |
| **ACER2** | -2.022296489 | 3.522313652 | 1.14E-67 | 2.52E-66 |
| **GFAP** | -2.02711679 | 1.795693034 | 1.57E-23 | 6.07E-23 |
| **NEGR1** | -2.030174683 | 2.934084948 | 4.56E-33 | 2.68E-32 |
| **AOC2** | -2.031733169 | 2.65984638 | 9.98E-28 | 4.61E-27 |
| **BTG2** | -2.034645766 | 8.47582527 | 5.22E-50 | 6.04E-49 |
| **ATF3** | -2.035505395 | 6.770845171 | 7.69E-42 | 6.36E-41 |
| **ROBO4** | -2.037943413 | 4.588142229 | 8.27E-68 | 1.83E-66 |
| **DTX1** | -2.039872256 | 4.202767328 | 7.44E-41 | 5.92E-40 |
| **C7** | -2.041081549 | 4.96191497 | 5.42E-17 | 1.56E-16 |
| **GPRASP1** | -2.041128397 | 3.581253508 | 2.88E-73 | 7.6E-72 |
| **NXNL2** | -2.041185768 | 2.127623671 | 5.41E-22 | 1.96E-21 |
| **KLHL4** | -2.042034991 | 1.755770565 | 6.44E-25 | 2.65E-24 |
| **TACC1** | -2.043382811 | 5.923106173 | 2.67E-61 | 4.64E-60 |
| **KLHDC1** | -2.045473021 | 2.927057854 | 1.71E-97 | 9.8E-96 |
| **FAM149A** | -2.0454974 | 3.001481617 | 1.35E-46 | 1.34E-45 |
| **PDE11A** | -2.048120545 | 1.849368493 | 2.01E-20 | 6.8E-20 |
| **SELP** | -2.051004214 | 4.435548719 | 1.84E-36 | 1.24E-35 |
| **SPRY1** | -2.053091104 | 6.378034474 | 2.35E-64 | 4.61E-63 |
| **TGFBR2** | -2.054152883 | 7.399461097 | 5.81E-51 | 6.98E-50 |
| **AMIGO2** | -2.055361762 | 6.019181645 | 7.95E-37 | 5.42E-36 |
| **F13A1** | -2.058168151 | 6.204749789 | 1.9E-27 | 8.69E-27 |
| **FAM189A2** | -2.062928977 | 4.151755481 | 3.47E-43 | 3.02E-42 |
| **MSRB3** | -2.063183856 | 5.496970479 | 4.54E-70 | 1.09E-68 |
| **ADAMTS8** | -2.065133534 | 2.323843188 | 6.98E-26 | 2.99E-25 |
| **SCN3B** | -2.066017641 | 1.841519607 | 5.31E-32 | 2.96E-31 |
| **FGB** | -2.066307273 | 1.772078661 | 3.66E-08 | 6.81E-08 |
| **CCL28** | -2.066543597 | 6.403439337 | 1.19E-19 | 3.88E-19 |
| **TBC1D9** | -2.069197205 | 6.555606879 | 3.76E-61 | 6.48E-60 |
| **ANKRD30B** | -2.069901406 | 1.712029182 | 1.11E-18 | 3.45E-18 |
| **PDZK1** | -2.070266791 | 3.98037186 | 5.11E-14 | 1.28E-13 |
| **IHO1** | -2.073228915 | 1.817029789 | 1.22E-29 | 6.15E-29 |
| **KLHDC7A** | -2.077587442 | 3.106359922 | 5.88E-16 | 1.62E-15 |
| **SYTL5** | -2.07782783 | 2.431880434 | 3.66E-20 | 1.22E-19 |
| **SRPX** | -2.080032103 | 6.79602411 | 8.64E-42 | 7.14E-41 |
| **TAL1** | -2.08229448 | 2.643958853 | 4.85E-57 | 7.27E-56 |
| **C10orf82** | -2.083438497 | 1.70183312 | 1.41E-20 | 4.81E-20 |
| **DMRT2** | -2.084153027 | 2.458039794 | 4.36E-23 | 1.66E-22 |
| **SLC1A7** | -2.086341931 | 1.868566816 | 1.08E-26 | 4.79E-26 |
| **TWIST2** | -2.089218967 | 4.716703988 | 1.08E-35 | 7.03E-35 |
| **THSD7B** | -2.093041285 | 2.155832565 | 2.48E-39 | 1.86E-38 |
| **FAM47E** | -2.093144042 | 1.771927266 | 6.77E-29 | 3.3E-28 |
| **HCAR3** | -2.095992221 | 1.861946784 | 1.24E-31 | 6.81E-31 |
| **ANGPTL2** | -2.096156079 | 7.374344223 | 1.04E-57 | 1.6E-56 |
| **LGI4** | -2.100847602 | 3.243278483 | 1.96E-46 | 1.93E-45 |
| **CCDC3** | -2.101581244 | 6.324624746 | 1.32E-28 | 6.34E-28 |
| **LIN7A** | -2.101862601 | 3.102884917 | 2.09E-40 | 1.64E-39 |
| **FMO2** | -2.103284556 | 6.056446831 | 1.68E-29 | 8.4E-29 |
| **FAM13A** | -2.10388406 | 4.195489884 | 3.84E-81 | 1.27E-79 |
| **ADGRF5** | -2.104468146 | 5.642531959 | 1.07E-64 | 2.13E-63 |
| **KLF9** | -2.104964378 | 6.238636169 | 7.13E-86 | 2.77E-84 |
| **PCDH19** | -2.105405378 | 2.454254416 | 3.04E-45 | 2.88E-44 |
| **SIK2** | -2.105504825 | 5.891142887 | 4.7E-63 | 8.75E-62 |
| **HIF3A** | -2.107236193 | 2.640636779 | 6.96E-30 | 3.57E-29 |
| **AKR1C2** | -2.107391535 | 5.714240024 | 1.08E-13 | 2.66E-13 |
| **SNCG** | -2.107812977 | 7.186337865 | 1.94E-20 | 6.54E-20 |
| **TSHZ2** | -2.108651994 | 4.173157931 | 3.85E-40 | 2.98E-39 |
| **RUNDC3B** | -2.109639784 | 2.335145057 | 1.59E-57 | 2.43E-56 |
| **SEMA5A** | -2.110530204 | 4.507330773 | 1.52E-52 | 1.94E-51 |
| **MAPT** | -2.112238478 | 3.754639917 | 3.58E-36 | 2.38E-35 |
| **PHYHD1** | -2.113969824 | 4.5061429 | 9.25E-39 | 6.8E-38 |
| **TMEM88** | -2.115646604 | 4.288943272 | 2.61E-57 | 3.95E-56 |
| **NAV3** | -2.116103759 | 2.341642673 | 3.66E-57 | 5.52E-56 |
| **DSC1** | -2.117549989 | 1.854673009 | 1.85E-28 | 8.84E-28 |
| **CXCL14** | -2.118082397 | 8.794995884 | 5.67E-24 | 2.24E-23 |
| **KCND3** | -2.121239515 | 3.412953597 | 4.01E-38 | 2.86E-37 |
| **CACNA2D1** | -2.122445001 | 3.810612931 | 1.18E-39 | 8.92E-39 |
| **GPR34** | -2.123540139 | 5.077182521 | 6.9E-48 | 7.29E-47 |
| **SEMA3C** | -2.123798845 | 6.881726342 | 1.27E-47 | 1.33E-46 |
| **IL6ST** | -2.125009614 | 7.066428445 | 1.39E-88 | 5.84E-87 |
| **NDNF** | -2.129471781 | 3.141630465 | 9.89E-26 | 4.2E-25 |
| **IL20RA** | -2.131042561 | 4.4256824 | 1.05E-31 | 5.77E-31 |
| **EPAS1** | -2.131478134 | 7.57399201 | 5.92E-70 | 1.4E-68 |
| **GCNT4** | -2.131490815 | 2.480681415 | 6.9E-65 | 1.38E-63 |
| **EGR2** | -2.1331439 | 6.010753381 | 3.09E-40 | 2.39E-39 |
| **RERGL** | -2.134443432 | 4.230567379 | 1.17E-17 | 3.45E-17 |
| **ADCYAP1R1** | -2.13463087 | 2.801007799 | 1.26E-28 | 6.09E-28 |
| **TPRG1** | -2.134805748 | 2.852201793 | 1.53E-45 | 1.46E-44 |
| **SCD** | -2.14165376 | 9.920047962 | 1.55E-21 | 5.52E-21 |
| **USHBP1** | -2.14259288 | 2.525669212 | 9.55E-58 | 1.47E-56 |
| **FCN2** | -2.144927244 | 2.749118654 | 1.41E-14 | 3.63E-14 |
| **ARHGEF15** | -2.147088936 | 4.511459981 | 2.66E-65 | 5.38E-64 |
| **FNDC5** | -2.147092406 | 2.988297626 | 5.75E-18 | 1.73E-17 |
| **TPPP** | -2.1535649 | 3.458294111 | 8.83E-54 | 1.18E-52 |
| **RETSAT** | -2.164330539 | 7.545594175 | 6.76E-53 | 8.79E-52 |
| **RRAD** | -2.164944871 | 3.69921361 | 3.02E-21 | 1.06E-20 |
| **LAMA3** | -2.166960554 | 5.010422065 | 3.98E-33 | 2.34E-32 |
| **ITM2A** | -2.168287534 | 6.876835329 | 4.91E-47 | 4.96E-46 |
| **CHAD** | -2.170136136 | 3.780181414 | 5.84E-21 | 2.03E-20 |
| **CCDC85A** | -2.17216466 | 1.873386703 | 9.39E-31 | 5E-30 |
| **CCDC178** | -2.175880168 | 1.832818471 | 1.65E-26 | 7.28E-26 |
| **TSLP** | -2.177677576 | 2.168052433 | 4.95E-49 | 5.5E-48 |
| **CLMP** | -2.182201757 | 6.20297229 | 1.86E-53 | 2.46E-52 |
| **LILRB5** | -2.18392853 | 3.253866755 | 1.14E-26 | 5.05E-26 |
| **CEBPA** | -2.190009831 | 5.082471823 | 9.31E-31 | 4.96E-30 |
| **GSTM2** | -2.192547358 | 2.793099687 | 3.1E-77 | 9.15E-76 |
| **KLF2** | -2.194604578 | 6.271605428 | 6.42E-53 | 8.37E-52 |
| **ACADL** | -2.197514235 | 2.908498547 | 4.01E-19 | 1.27E-18 |
| **FYB2** | -2.203135042 | 1.912266449 | 2.68E-27 | 1.22E-26 |
| **NR3C2** | -2.205640938 | 3.799816558 | 1.42E-104 | 9.87E-103 |
| **SHE** | -2.207027722 | 3.391849572 | 2.3E-72 | 5.85E-71 |
| **TEK** | -2.207330776 | 4.597280589 | 9.59E-58 | 1.47E-56 |
| **PPP1R12B** | -2.21323279 | 4.652148547 | 1.05E-103 | 7.18E-102 |
| **TTC6** | -2.214811247 | 2.350207127 | 3.14E-25 | 1.31E-24 |
| **TNMD** | -2.218041702 | 4.654535377 | 6.29E-12 | 1.42E-11 |
| **FAM13C** | -2.219328503 | 2.41268005 | 4.92E-69 | 1.14E-67 |
| **S1PR1** | -2.219855147 | 5.4811134 | 5.51E-68 | 1.23E-66 |
| **PDZRN4** | -2.22166427 | 1.877350561 | 2E-27 | 9.13E-27 |
| **PNMA8C** | -2.22286701 | 1.806575416 | 1.17E-21 | 4.19E-21 |
| **ABI3BP** | -2.224493925 | 4.686893807 | 6.38E-35 | 4.02E-34 |
| **TNN** | -2.235101836 | 3.541107449 | 8.84E-41 | 7.02E-40 |
| **APOLD1** | -2.236731846 | 5.546411917 | 2.85E-44 | 2.59E-43 |
| **TMEM26** | -2.249938161 | 2.779701716 | 8.14E-36 | 5.32E-35 |
| **SLC22A3** | -2.255708222 | 3.501846364 | 8.25E-36 | 5.39E-35 |
| **LYPD6** | -2.256207227 | 3.688367215 | 4.08E-37 | 2.8E-36 |
| **PODN** | -2.26138391 | 5.741094997 | 6.03E-49 | 6.67E-48 |
| **ALX4** | -2.262703599 | 3.056118399 | 2.87E-29 | 1.43E-28 |
| **TFPI2** | -2.26274438 | 5.031651672 | 2.06E-19 | 6.61E-19 |
| **KLK3** | -2.262754777 | 1.877611985 | 1.35E-09 | 2.71E-09 |
| **TBX15** | -2.2631209 | 4.224405548 | 2.14E-38 | 1.55E-37 |
| **CAV2** | -2.264891162 | 6.265271643 | 3.41E-55 | 4.8E-54 |
| **CYP4Z1** | -2.265118236 | 5.061277389 | 1.09E-11 | 2.43E-11 |
| **ABCG2** | -2.267145784 | 2.835923791 | 1.5E-63 | 2.84E-62 |
| **PLSCR4** | -2.270260897 | 5.606026422 | 1.11E-81 | 3.76E-80 |
| **IQCN** | -2.276901519 | 2.757883459 | 1.97E-63 | 3.73E-62 |
| **PFKFB3** | -2.277618075 | 7.500854605 | 2.63E-52 | 3.34E-51 |
| **SLC13A2** | -2.278668734 | 4.04428877 | 1.51E-15 | 4.08E-15 |
| **EHD2** | -2.28248317 | 7.81358627 | 7.64E-64 | 1.47E-62 |
| **TRABD2B** | -2.284320917 | 2.88196193 | 5.53E-53 | 7.22E-52 |
| **PDLIM3** | -2.284724617 | 5.257461106 | 5E-36 | 3.3E-35 |
| **CYP4F22** | -2.285007719 | 4.047374105 | 1.52E-14 | 3.9E-14 |
| **PLAAT3** | -2.288732141 | 7.290402636 | 3.27E-41 | 2.64E-40 |
| **FGF16** | -2.290815611 | 1.748367036 | 1.03E-19 | 3.35E-19 |
| **THRB** | -2.290910356 | 4.138376305 | 9.91E-113 | 8.38E-111 |
| **MRGPRF** | -2.292477334 | 3.74863717 | 2.3E-69 | 5.38E-68 |
| **ZCCHC24** | -2.293281284 | 5.606593478 | 3.52E-81 | 1.16E-79 |
| **UGT2B11** | -2.295404913 | 5.018255763 | 1.15E-06 | 1.97E-06 |
| **AFF3** | -2.297179148 | 3.829458783 | 1.91E-37 | 1.33E-36 |
| **MMRN2** | -2.297385914 | 5.902294698 | 3.74E-80 | 1.21E-78 |
| **HSD11B1** | -2.297713488 | 4.857573902 | 8.23E-30 | 4.2E-29 |
| **ENPP2** | -2.302005653 | 6.188783233 | 6.31E-45 | 5.87E-44 |
| **CSRNP1** | -2.305934176 | 6.720797893 | 6.85E-73 | 1.77E-71 |
| **DIPK2B** | -2.306783151 | 4.901240655 | 1.24E-95 | 6.71E-94 |
| **FGF2** | -2.308483773 | 4.437518273 | 3.42E-50 | 3.99E-49 |
| **FAM162B** | -2.309706392 | 3.070804611 | 2.6E-58 | 4.07E-57 |
| **KY** | -2.310859165 | 2.006028191 | 1.02E-34 | 6.37E-34 |
| **GIPC2** | -2.310883712 | 3.104075121 | 1.09E-66 | 2.34E-65 |
| **TPSG1** | -2.311492635 | 1.727019037 | 4.51E-21 | 1.57E-20 |
| **CEP126** | -2.314767152 | 3.195772307 | 3.91E-87 | 1.58E-85 |
| **DDR2** | -2.315309672 | 6.07615566 | 1.59E-47 | 1.64E-46 |
| **PHYHIP** | -2.315644662 | 2.70791133 | 1.06E-46 | 1.05E-45 |
| **OMD** | -2.31565746 | 4.606468364 | 9.59E-40 | 7.29E-39 |
| **ITPR1** | -2.316117493 | 3.752319764 | 9.62E-77 | 2.76E-75 |
| **TAC1** | -2.316729139 | 2.183482635 | 1.12E-19 | 3.63E-19 |
| **NMUR1** | -2.320093199 | 2.746744575 | 5.46E-30 | 2.81E-29 |
| **MYCT1** | -2.321551382 | 4.849448992 | 3.52E-84 | 1.3E-82 |
| **CCDC80** | -2.326612562 | 7.116758925 | 1.74E-46 | 1.72E-45 |
| **RBM24** | -2.32691011 | 2.947846526 | 2.22E-19 | 7.12E-19 |
| **EMCN** | -2.32798687 | 4.902982255 | 4.18E-88 | 1.73E-86 |
| **NAALAD2** | -2.330707294 | 2.234366434 | 1.67E-34 | 1.03E-33 |
| **NIPSNAP3B** | -2.334167338 | 3.354356397 | 5.32E-55 | 7.42E-54 |
| **LRRC70** | -2.334814882 | 1.850459217 | 1.03E-38 | 7.55E-38 |
| **AGR3** | -2.335435394 | 5.57942759 | 1.57E-13 | 3.85E-13 |
| **PDZD2** | -2.336906324 | 4.536691013 | 9.21E-44 | 8.2E-43 |
| **FGF1** | -2.340019102 | 4.86175816 | 4.06E-84 | 1.49E-82 |
| **LHFPL6** | -2.340409488 | 6.23498016 | 4.09E-97 | 2.31E-95 |
| **SELENOP** | -2.344353596 | 5.863408212 | 1.83E-70 | 4.44E-69 |
| **PEAR1** | -2.345259268 | 3.863334162 | 8.25E-96 | 4.5E-94 |
| **STAB2** | -2.346699057 | 1.763912346 | 3.08E-28 | 1.46E-27 |
| **SEMA6D** | -2.349789231 | 3.4032612 | 7.61E-59 | 1.21E-57 |
| **IL33** | -2.352747823 | 5.858824254 | 1.18E-34 | 7.34E-34 |
| **TPO** | -2.352753195 | 2.022042132 | 6.19E-47 | 6.23E-46 |
| **DNASE1L3** | -2.354945981 | 3.0642496 | 1.65E-38 | 1.2E-37 |
| **SPARCL1** | -2.354994059 | 9.152505835 | 6.54E-72 | 1.65E-70 |
| **CBLN4** | -2.356906215 | 2.137159255 | 3.54E-24 | 1.41E-23 |
| **SH3D19** | -2.357045177 | 6.148832477 | 2.47E-94 | 1.27E-92 |
| **DCDC2** | -2.359389662 | 3.459413565 | 1.22E-25 | 5.17E-25 |
| **TNS2** | -2.360563854 | 6.097791145 | 5.19E-98 | 3.06E-96 |
| **MASP1** | -2.36063708 | 1.790045805 | 1.06E-26 | 4.72E-26 |
| **ELANE** | -2.362156138 | 1.731032717 | 5.44E-27 | 2.45E-26 |
| **CXCL12** | -2.362613394 | 7.402818727 | 3.36E-89 | 1.43E-87 |
| **GRIK1** | -2.364037336 | 1.886740981 | 5.27E-24 | 2.09E-23 |
| **GFRA1** | -2.36730156 | 5.35286779 | 3.12E-23 | 1.2E-22 |
| **IL6** | -2.368087904 | 4.246531584 | 7.05E-25 | 2.89E-24 |
| **ACTC1** | -2.369350251 | 1.98329673 | 3.55E-10 | 7.34E-10 |
| **TMTC1** | -2.371867113 | 4.447250396 | 3.31E-47 | 3.36E-46 |
| **PTH1R** | -2.372156232 | 3.269695023 | 4.64E-36 | 3.06E-35 |
| **NOSTRIN** | -2.38104352 | 4.156778116 | 2.77E-82 | 9.63E-81 |
| **RBMS3** | -2.381127083 | 3.299164528 | 1.62E-94 | 8.48E-93 |
| **TCEAL7** | -2.382669423 | 4.130096128 | 1.68E-62 | 3.04E-61 |
| **COL17A1** | -2.383011872 | 6.544830605 | 2.24E-16 | 6.28E-16 |
| **RNF186** | -2.387637623 | 1.947485698 | 1.33E-22 | 4.97E-22 |
| **FAM110D** | -2.390273525 | 3.515598311 | 2.79E-64 | 5.45E-63 |
| **ALDH2** | -2.391846288 | 6.467667464 | 4.29E-44 | 3.86E-43 |
| **HSPB8** | -2.393804853 | 5.755992866 | 2.08E-35 | 1.33E-34 |
| **ABCC9** | -2.395242304 | 3.694760781 | 6.74E-42 | 5.59E-41 |
| **MYL7** | -2.40109535 | 2.307345265 | 4.88E-19 | 1.54E-18 |
| **PTPRB** | -2.401967373 | 3.926465008 | 3.24E-73 | 8.55E-72 |
| **GSN** | -2.402767798 | 9.012827314 | 3.47E-83 | 1.25E-81 |
| **CLDN11** | -2.403790746 | 4.419279977 | 5.41E-49 | 6E-48 |
| **VWF** | -2.404735559 | 7.352608209 | 7.66E-67 | 1.65E-65 |
| **TMEM255A** | -2.406462351 | 3.269802172 | 7.2E-79 | 2.23E-77 |
| **TXNIP** | -2.407339402 | 10.50818429 | 3.93E-91 | 1.81E-89 |
| **DST** | -2.410371192 | 5.681854157 | 1.92E-86 | 7.63E-85 |
| **CORO6** | -2.416949333 | 2.574335975 | 7.02E-22 | 2.54E-21 |
| **COL14A1** | -2.422228875 | 6.337404915 | 3.29E-46 | 3.2E-45 |
| **MEOX1** | -2.423952436 | 4.920260917 | 1.78E-38 | 1.29E-37 |
| **AHNAK** | -2.424684914 | 8.903175156 | 1.25E-105 | 8.85E-104 |
| **LDHD** | -2.425158696 | 4.505129694 | 9.79E-31 | 5.2E-30 |
| **CORO2B** | -2.426978752 | 3.608025685 | 1.12E-73 | 3.01E-72 |
| **RAB6C** | -2.429625925 | 1.712287238 | 6.07E-29 | 2.97E-28 |
| **LRRN3** | -2.433701484 | 3.073675663 | 8.33E-31 | 4.44E-30 |
| **CD34** | -2.433943261 | 6.188693368 | 6.02E-98 | 3.54E-96 |
| **TRMT9B** | -2.435374118 | 2.489213493 | 4.74E-72 | 1.2E-70 |
| **B3GALT1** | -2.441869901 | 1.851390768 | 1.41E-33 | 8.44E-33 |
| **GNG11** | -2.442722399 | 6.376957832 | 6.33E-82 | 2.18E-80 |
| **PNPLA2** | -2.443256002 | 7.538101369 | 1.51E-67 | 3.3E-66 |
| **ZFP36** | -2.448158086 | 9.644142824 | 4.94E-51 | 5.96E-50 |
| **DMGDH** | -2.451035814 | 2.546337571 | 2.15E-39 | 1.61E-38 |
| **COL4A6** | -2.452405844 | 2.792083055 | 1.35E-37 | 9.43E-37 |
| **MYRIP** | -2.452803822 | 2.639899214 | 7.56E-71 | 1.85E-69 |
| **NTN4** | -2.45652108 | 6.950023207 | 1.48E-53 | 1.96E-52 |
| **ADH4** | -2.456610819 | 1.892598351 | 1.19E-23 | 4.62E-23 |
| **GPR146** | -2.457222189 | 2.401081955 | 1.82E-51 | 2.24E-50 |
| **HPSE2** | -2.459986228 | 2.841708214 | 1.39E-38 | 1.01E-37 |
| **LDB2** | -2.461593871 | 4.636854774 | 3.39E-107 | 2.56E-105 |
| **ABCB1** | -2.464827081 | 3.210898896 | 3.08E-90 | 1.36E-88 |
| **PER1** | -2.469842322 | 5.739409866 | 4.49E-85 | 1.7E-83 |
| **ANK2** | -2.471939751 | 3.267482332 | 2.58E-60 | 4.27E-59 |
| **TSPAN7** | -2.477933034 | 5.055240612 | 4.51E-58 | 6.99E-57 |
| **PALM** | -2.479086604 | 5.34401089 | 6.22E-50 | 7.17E-49 |
| **ALPK3** | -2.479278282 | 3.574556609 | 3.71E-31 | 2E-30 |
| **ADGRD1** | -2.479862358 | 2.277537975 | 3.08E-40 | 2.39E-39 |
| **ARHGAP40** | -2.482961131 | 4.070736628 | 1.31E-22 | 4.89E-22 |
| **JAM2** | -2.490753596 | 4.727662311 | 2.12E-128 | 2.41E-126 |
| **MMP27** | -2.491969534 | 1.846754042 | 1.27E-34 | 7.87E-34 |
| **STC2** | -2.493887944 | 7.015124623 | 4.16E-44 | 3.75E-43 |
| **RHOXF1** | -2.494361701 | 2.114501337 | 9.49E-35 | 5.94E-34 |
| **CCDC158** | -2.498418574 | 2.109263496 | 6.15E-60 | 1.01E-58 |
| **ACKR3** | -2.499949945 | 6.818334695 | 3.41E-56 | 4.96E-55 |
| **SOX17** | -2.501026495 | 4.075679801 | 2.04E-55 | 2.9E-54 |
| **MYOZ3** | -2.501438626 | 1.957633979 | 4.96E-20 | 1.64E-19 |
| **CLCA4** | -2.505656709 | 2.224707357 | 2.76E-34 | 1.69E-33 |
| **MOCS1** | -2.50720954 | 4.905077428 | 1.24E-72 | 3.2E-71 |
| **GPBAR1** | -2.507605654 | 2.630283381 | 2.79E-35 | 1.78E-34 |
| **CYBRD1** | -2.507968366 | 8.165825631 | 7.72E-85 | 2.9E-83 |
| **BHMT2** | -2.510152832 | 4.636321277 | 1.4E-29 | 7.03E-29 |
| **ADAMTS15** | -2.510374158 | 4.645365016 | 2.64E-47 | 2.7E-46 |
| **SAA1** | -2.514916461 | 10.62714537 | 2.38E-26 | 1.04E-25 |
| **MFAP5** | -2.517817618 | 6.807391762 | 8.36E-37 | 5.7E-36 |
| **THSD4** | -2.519741954 | 4.159406981 | 9.63E-50 | 1.1E-48 |
| **IGSF1** | -2.52234711 | 3.014294659 | 3.94E-26 | 1.71E-25 |
| **PTPRT** | -2.536344487 | 3.079941503 | 2.05E-37 | 1.42E-36 |
| **PALMD** | -2.545290447 | 5.756885154 | 4.62E-66 | 9.67E-65 |
| **PPP1R3C** | -2.547548535 | 4.961653405 | 1.61E-33 | 9.59E-33 |
| **NKAPL** | -2.547564983 | 2.307735807 | 7.19E-63 | 1.32E-61 |
| **SLC4A4** | -2.548740562 | 2.517745002 | 2.64E-30 | 1.38E-29 |
| **TCEAL5** | -2.557138531 | 3.01419161 | 1.17E-36 | 7.92E-36 |
| **NAT8L** | -2.559966687 | 5.137891684 | 4.87E-32 | 2.73E-31 |
| **SAMD5** | -2.563060595 | 4.045462524 | 8.56E-44 | 7.63E-43 |
| **EBF2** | -2.565313779 | 2.954172917 | 6.65E-48 | 7.03E-47 |
| **SLC17A7** | -2.569711935 | 2.141584389 | 4.28E-44 | 3.86E-43 |
| **LDLRAD2** | -2.573763113 | 1.92301242 | 2.54E-42 | 2.14E-41 |
| **HCAR1** | -2.576192705 | 4.879313429 | 1.83E-73 | 4.87E-72 |
| **TMEM178A** | -2.580365608 | 3.316465234 | 4.47E-40 | 3.44E-39 |
| **ADAMTS5** | -2.582722853 | 4.877759149 | 2.36E-94 | 1.22E-92 |
| **RAI2** | -2.594631242 | 5.344247854 | 2.73E-86 | 1.08E-84 |
| **ACSL1** | -2.602643342 | 8.136538563 | 3.22E-47 | 3.28E-46 |
| **CLIC5** | -2.607379605 | 3.174176719 | 2.6E-49 | 2.92E-48 |
| **RAPGEF3** | -2.609635414 | 3.383428142 | 9.35E-104 | 6.45E-102 |
| **ALDH1A1** | -2.613105957 | 6.763222732 | 5.51E-54 | 7.44E-53 |
| **ABCC6** | -2.614320051 | 2.674258519 | 4.44E-45 | 4.16E-44 |
| **NTS** | -2.615432916 | 3.352830215 | 1.16E-11 | 2.58E-11 |
| **ADRA2A** | -2.616496168 | 5.194736085 | 2.36E-60 | 3.92E-59 |
| **PREX2** | -2.617481161 | 3.365404378 | 1.04E-54 | 1.45E-53 |
| **IGF1** | -2.621762061 | 2.482603134 | 1.61E-46 | 1.59E-45 |
| **ANGPTL7** | -2.621791275 | 3.537804242 | 3.88E-19 | 1.23E-18 |
| **MATN2** | -2.622181346 | 6.021669483 | 2.35E-83 | 8.46E-82 |
| **SLC7A2** | -2.625526875 | 5.030019222 | 4.89E-44 | 4.4E-43 |
| **C16orf89** | -2.626627749 | 4.911349596 | 6.77E-36 | 4.44E-35 |
| **EBF3** | -2.627003146 | 3.529231515 | 5.07E-50 | 5.87E-49 |
| **TP53AIP1** | -2.630382973 | 2.228228212 | 2.94E-38 | 2.11E-37 |
| **MYOM2** | -2.638475753 | 3.822294028 | 4.39E-12 | 1E-11 |
| **AKR1C1** | -2.638854633 | 4.830849548 | 4.46E-29 | 2.2E-28 |
| **TP63** | -2.644202926 | 4.849673844 | 2.44E-30 | 1.28E-29 |
| **PAK5** | -2.663519647 | 2.019989764 | 4.94E-47 | 4.99E-46 |
| **LRRC39** | -2.664934868 | 2.570331025 | 5.03E-22 | 1.83E-21 |
| **CTSG** | -2.675275461 | 3.727410198 | 1.42E-36 | 9.59E-36 |
| **CLDN5** | -2.679575415 | 5.412296454 | 1.22E-40 | 9.61E-40 |
| **DUSP1** | -2.679659299 | 9.406479782 | 4.22E-69 | 9.83E-68 |
| **DCN** | -2.683716459 | 8.692163936 | 1.34E-77 | 4E-76 |
| **PRKAR2B** | -2.694390008 | 7.30492456 | 9.72E-45 | 8.99E-44 |
| **PHGR1** | -2.697090805 | 1.81824076 | 2.35E-23 | 9.03E-23 |
| **CSF3** | -2.712163345 | 2.284877455 | 1.94E-14 | 4.96E-14 |
| **ALDH1A2** | -2.713756023 | 2.853106504 | 2.66E-62 | 4.78E-61 |
| **SGCA** | -2.719389771 | 2.579097221 | 3.1E-21 | 1.08E-20 |
| **MTARC1** | -2.720119269 | 4.294065116 | 1.31E-47 | 1.37E-46 |
| **MTURN** | -2.720737447 | 6.285553526 | 2.88E-78 | 8.82E-77 |
| **AK5** | -2.723726135 | 4.7049419 | 3.58E-35 | 2.28E-34 |
| **LAMA2** | -2.728360142 | 4.57204957 | 1.38E-75 | 3.92E-74 |
| **PDGFD** | -2.733415487 | 5.50452066 | 8.46E-139 | 1.26E-136 |
| **MME** | -2.741675875 | 5.97287139 | 6.23E-50 | 7.17E-49 |
| **LIFR** | -2.752799955 | 5.303338462 | 2.47E-91 | 1.16E-89 |
| **NTRK2** | -2.752851323 | 5.54388376 | 1.48E-51 | 1.82E-50 |
| **P2RY12** | -2.771907078 | 3.21464809 | 2.08E-67 | 4.51E-66 |
| **DACH1** | -2.773785526 | 3.457231081 | 2.77E-78 | 8.51E-77 |
| **OVCH2** | -2.777921467 | 2.387308633 | 3.1E-41 | 2.52E-40 |
| **SYNE3** | -2.805188364 | 3.906349588 | 5.18E-64 | 1E-62 |
| **BMX** | -2.805410572 | 3.053676924 | 6.67E-77 | 1.93E-75 |
| **LUZP2** | -2.810566653 | 1.904712874 | 6.02E-33 | 3.51E-32 |
| **FAXDC2** | -2.811734145 | 5.050810863 | 6.54E-148 | 1.14E-145 |
| **ANKRD29** | -2.811924351 | 3.340521579 | 4.14E-128 | 4.62E-126 |
| **CCDC170** | -2.812956413 | 3.458912265 | 1.08E-104 | 7.55E-103 |
| **AOX1** | -2.814042031 | 4.656925605 | 1.86E-54 | 2.56E-53 |
| **CXCL2** | -2.818472007 | 5.504381988 | 1.96E-56 | 2.88E-55 |
| **FITM1** | -2.819845387 | 2.037579962 | 2.46E-19 | 7.87E-19 |
| **RXRG** | -2.821974347 | 2.062130024 | 7.06E-29 | 3.44E-28 |
| **FREM1** | -2.83559555 | 3.200731521 | 7.98E-58 | 1.23E-56 |
| **MMD** | -2.836057316 | 6.538424773 | 1.05E-62 | 1.92E-61 |
| **TGFBR3** | -2.853705633 | 6.223791343 | 5.16E-85 | 1.95E-83 |
| **MGLL** | -2.854357848 | 6.169844482 | 1.11E-77 | 3.3E-76 |
| **HLF** | -2.854551273 | 4.417001545 | 3.49E-83 | 1.25E-81 |
| **MYOM3** | -2.862231894 | 2.101238267 | 2.63E-24 | 1.05E-23 |
| **ITLN1** | -2.863605029 | 1.850414445 | 3.28E-13 | 7.9E-13 |
| **PLIN5** | -2.867136328 | 3.05176983 | 1.33E-39 | 1.01E-38 |
| **CCDC69** | -2.885159612 | 6.255590685 | 6.92E-65 | 1.38E-63 |
| **PGM5** | -2.889958179 | 3.816209121 | 2.46E-71 | 6.1E-70 |
| **PRG4** | -2.896699445 | 4.901838494 | 2.77E-17 | 8.08E-17 |
| **CST9** | -2.898481337 | 2.488498676 | 2.4E-13 | 5.82E-13 |
| **PLN** | -2.900246657 | 4.246511654 | 2.3E-31 | 1.25E-30 |
| **AADAC** | -2.908559534 | 3.149248632 | 3.15E-17 | 9.17E-17 |
| **AFP** | -2.908856856 | 2.632950284 | 4.62E-20 | 1.53E-19 |
| **KL** | -2.915849888 | 2.747693891 | 8.24E-108 | 6.35E-106 |
| **C1QTNF9** | -2.926800366 | 1.823650911 | 5.41E-47 | 5.45E-46 |
| **RGS22** | -2.932830891 | 2.041942346 | 1.63E-40 | 1.28E-39 |
| **SCUBE2** | -2.939912042 | 5.474811941 | 2.96E-42 | 2.48E-41 |
| **CAPN11** | -2.942786261 | 2.740836745 | 5.96E-109 | 4.69E-107 |
| **SCN4B** | -2.946324437 | 4.407764898 | 1.96E-102 | 1.31E-100 |
| **SMCO1** | -2.949978897 | 1.970015915 | 1.41E-06 | 2.4E-06 |
| **SRARP** | -2.958416757 | 2.356573316 | 2.63E-31 | 1.43E-30 |
| **FLNC** | -2.962355583 | 4.715675147 | 2.43E-31 | 1.33E-30 |
| **ADRB2** | -2.963556032 | 4.45485404 | 1.13E-82 | 3.98E-81 |
| **F10** | -2.96652464 | 3.571111631 | 3.88E-66 | 8.15E-65 |
| **EGR1** | -2.96748533 | 10.07842928 | 2.89E-67 | 6.24E-66 |
| **KLF15** | -2.974815507 | 4.195254734 | 9.93E-63 | 1.81E-61 |
| **INSYN2B** | -2.975456758 | 3.54210399 | 6.08E-40 | 4.66E-39 |
| **ACKR1** | -2.984313926 | 6.936433763 | 1.43E-47 | 1.48E-46 |
| **THRSP** | -3.008207768 | 7.484148821 | 2.02E-21 | 7.16E-21 |
| **EGR3** | -3.011837742 | 5.781301481 | 1.47E-50 | 1.74E-49 |
| **SLC16A7** | -3.013390444 | 3.67487405 | 5.03E-57 | 7.53E-56 |
| **ANKRD30A** | -3.017586678 | 3.90789806 | 8.52E-21 | 2.93E-20 |
| **EBF1** | -3.019400989 | 5.108863873 | 3.21E-62 | 5.77E-61 |
| **KLF4** | -3.020977979 | 6.673668166 | 4.01E-89 | 1.7E-87 |
| **SLC16A12** | -3.024175057 | 1.864368068 | 2.05E-42 | 1.73E-41 |
| **SGK2** | -3.027249645 | 2.704996342 | 4.1E-42 | 3.43E-41 |
| **TMEM252** | -3.029269189 | 1.794556985 | 2.2E-50 | 2.59E-49 |
| **GLDN** | -3.036973239 | 2.752708214 | 4.13E-49 | 4.61E-48 |
| **SLIT3** | -3.046161338 | 5.221122406 | 7.19E-99 | 4.38E-97 |
| **ERBB4** | -3.057106398 | 3.17188221 | 1.55E-58 | 2.45E-57 |
| **HMGCS2** | -3.057591531 | 6.620780445 | 3.12E-13 | 7.53E-13 |
| **HCAR2** | -3.06521389 | 4.32480979 | 7.94E-52 | 9.92E-51 |
| **CLSTN2** | -3.07177676 | 4.985451448 | 4.92E-42 | 4.1E-41 |
| **NEK10** | -3.074582243 | 2.644444502 | 1E-49 | 1.14E-48 |
| **TNNT1** | -3.083196385 | 6.446538771 | 1.05E-11 | 2.35E-11 |
| **SLC7A3** | -3.086981274 | 2.710973017 | 3.19E-41 | 2.58E-40 |
| **CNN1** | -3.088698528 | 7.195090406 | 1.99E-45 | 1.89E-44 |
| **LMOD1** | -3.091108148 | 5.980707894 | 1.83E-98 | 1.1E-96 |
| **INMT** | -3.092159669 | 3.951323755 | 7.58E-80 | 2.43E-78 |
| **CHL1** | -3.099775803 | 3.800452019 | 1.57E-92 | 7.67E-91 |
| **ABCA6** | -3.102638961 | 3.017915846 | 1.52E-90 | 6.79E-89 |
| **SMTNL1** | -3.108767829 | 2.939566662 | 2.41E-18 | 7.36E-18 |
| **PPP1R1A** | -3.111571131 | 6.508302702 | 1.28E-35 | 8.27E-35 |
| **ADCY5** | -3.111692168 | 3.701905054 | 2.2E-106 | 1.58E-104 |
| **MAB21L1** | -3.112247191 | 2.996309235 | 3.17E-94 | 1.61E-92 |
| **PGR** | -3.120322589 | 3.943112159 | 3.57E-32 | 2.01E-31 |
| **FHL5** | -3.141081673 | 3.62493123 | 1.5E-61 | 2.63E-60 |
| **TMOD4** | -3.144547737 | 2.854189054 | 4.33E-24 | 1.72E-23 |
| **ESR1** | -3.145360219 | 4.780796326 | 2.77E-46 | 2.71E-45 |
| **C6** | -3.153458795 | 3.273029854 | 2.21E-24 | 8.85E-24 |
| **PLXNA4** | -3.157300327 | 3.87901121 | 1.66E-48 | 1.8E-47 |
| **ADRA1A** | -3.159480685 | 2.059905216 | 1.36E-33 | 8.13E-33 |
| **TMEM37** | -3.178161339 | 6.233722279 | 9.67E-66 | 2E-64 |
| **MYZAP** | -3.193871849 | 4.025527732 | 1.19E-69 | 2.79E-68 |
| **AKAP12** | -3.209156057 | 5.776931247 | 4.26E-81 | 1.41E-79 |
| **PDE3B** | -3.229786306 | 4.239943015 | 2.16E-44 | 1.97E-43 |
| **SYPL2** | -3.231866048 | 2.583427535 | 1.35E-26 | 5.99E-26 |
| **MAMDC2** | -3.232062527 | 4.657141102 | 8.68E-100 | 5.38E-98 |
| **FZD4** | -3.232214463 | 6.320863291 | 1.36E-89 | 5.95E-88 |
| **COX7A1** | -3.234494093 | 7.188191187 | 9.19E-65 | 1.83E-63 |
| **MYBPC1** | -3.234925406 | 6.8399568 | 3.56E-15 | 9.42E-15 |
| **PRRT4** | -3.237254059 | 2.948609668 | 5.65E-36 | 3.72E-35 |
| **LEPR** | -3.242267752 | 4.227688622 | 1.18E-110 | 9.43E-109 |
| **IGFBP6** | -3.246167121 | 7.226897899 | 5.98E-63 | 1.11E-61 |
| **CAV1** | -3.258881296 | 8.775915079 | 1.31E-83 | 4.74E-82 |
| **PLAC9** | -3.268439506 | 6.188079255 | 3.81E-52 | 4.83E-51 |
| **FOS** | -3.271478168 | 9.88717424 | 1.6E-62 | 2.9E-61 |
| **PLAAT5** | -3.272259347 | 5.209404951 | 9.38E-47 | 9.39E-46 |
| **ADAM33** | -3.273413327 | 3.754714869 | 4.7E-107 | 3.53E-105 |
| **FRMD1** | -3.273961209 | 1.852539961 | 2.41E-32 | 1.37E-31 |
| **LRRC2** | -3.275083879 | 2.352538632 | 2.6E-41 | 2.11E-40 |
| **TRIM54** | -3.278254706 | 1.990268467 | 4.41E-15 | 1.16E-14 |
| **CKMT2** | -3.2950309 | 3.986135137 | 1.71E-35 | 1.1E-34 |
| **SLC14A2** | -3.298628678 | 1.981069717 | 1.8E-37 | 1.26E-36 |
| **MFAP4** | -3.303582932 | 8.150407634 | 3.62E-68 | 8.22E-67 |
| **SCGB1A1** | -3.319546313 | 2.434292784 | 4.85E-09 | 9.46E-09 |
| **IGFBP1** | -3.329206658 | 2.082634915 | 6.94E-28 | 3.24E-27 |
| **ARHGAP20** | -3.330215654 | 3.079707294 | 3.58E-119 | 3.35E-117 |
| **AVPR2** | -3.332439768 | 3.051653801 | 9.63E-88 | 3.97E-86 |
| **C1QTNF7** | -3.335443999 | 2.885226504 | 6.09E-43 | 5.26E-42 |
| **SCN7A** | -3.342962011 | 2.053783048 | 2.03E-62 | 3.67E-61 |
| **COL6A6** | -3.344000955 | 2.956540416 | 8.21E-83 | 2.92E-81 |
| **ATOH8** | -3.34504445 | 3.01689292 | 2.05E-82 | 7.15E-81 |
| **GPC3** | -3.347995574 | 6.859371189 | 8.99E-62 | 1.59E-60 |
| **ABLIM3** | -3.367328271 | 4.916686558 | 7.82E-116 | 7.02E-114 |
| **SULT1C3** | -3.369394412 | 5.782925706 | 8.13E-07 | 1.4E-06 |
| **BMP3** | -3.38045536 | 2.028058009 | 8.03E-23 | 3.02E-22 |
| **CPB1** | -3.381996154 | 4.690858673 | 7.32E-19 | 2.29E-18 |
| **ECM2** | -3.394328332 | 5.901457882 | 2.4E-94 | 1.24E-92 |
| **SYNPO2** | -3.420106112 | 5.036477971 | 5.26E-90 | 2.3E-88 |
| **TNS1** | -3.427811476 | 7.36835578 | 1.78E-113 | 1.53E-111 |
| **ANKRD2** | -3.428376387 | 2.408731318 | 8.88E-18 | 2.64E-17 |
| **ABCB5** | -3.42998781 | 1.780484804 | 3.11E-50 | 3.64E-49 |
| **CCL14** | -3.431598846 | 2.020074959 | 9.63E-66 | 1.99E-64 |
| **DUSP26** | -3.434645178 | 2.337720618 | 1.97E-19 | 6.35E-19 |
| **GPX3** | -3.436385108 | 9.212683393 | 1.99E-60 | 3.31E-59 |
| **KLHL31** | -3.438642831 | 3.607503262 | 1.02E-52 | 1.31E-51 |
| **ANGPTL5** | -3.441983664 | 1.920936323 | 4.88E-30 | 2.52E-29 |
| **ADH1C** | -3.455568037 | 4.449761868 | 1.4E-47 | 1.45E-46 |
| **ITGA7** | -3.461047978 | 5.886220241 | 4.28E-62 | 7.66E-61 |
| **PCOLCE2** | -3.461941147 | 6.298038657 | 2.67E-45 | 2.53E-44 |
| **CACNG1** | -3.46934778 | 2.350153583 | 3.33E-19 | 1.06E-18 |
| **CMA1** | -3.469744381 | 3.461165187 | 3.81E-58 | 5.93E-57 |
| **XIRP1** | -3.473295516 | 3.861592039 | 8.47E-12 | 1.9E-11 |
| **CPED1** | -3.478408396 | 4.654544313 | 1.81E-82 | 6.35E-81 |
| **ANGPT4** | -3.479682701 | 2.001707705 | 8.62E-53 | 1.12E-51 |
| **GRIA4** | -3.494134839 | 2.356636585 | 2.52E-60 | 4.17E-59 |
| **SEMA3G** | -3.518586937 | 6.186113892 | 2.14E-95 | 1.14E-93 |
| **CDO1** | -3.519238207 | 5.885243581 | 1.09E-56 | 1.62E-55 |
| **MB** | -3.538893783 | 8.026204694 | 2.94E-20 | 9.84E-20 |
| **TNNT3** | -3.547868589 | 6.093067681 | 9.27E-16 | 2.53E-15 |
| **PPARG** | -3.559207548 | 5.510245359 | 4.73E-78 | 1.44E-76 |
| **UGT2B15** | -3.563221793 | 2.653928209 | 2.03E-27 | 9.27E-27 |
| **PTGER3** | -3.564961154 | 4.55580022 | 2.92E-91 | 1.35E-89 |
| **PEBP4** | -3.597440246 | 3.264654008 | 4.38E-29 | 2.16E-28 |
| **ABCD2** | -3.597445731 | 3.303705805 | 8.93E-55 | 1.24E-53 |
| **ANGPTL1** | -3.602066408 | 4.178061094 | 8.75E-85 | 3.27E-83 |
| **TNNI2** | -3.606557881 | 6.822293385 | 1.32E-19 | 4.27E-19 |
| **CPA1** | -3.615436427 | 1.879797108 | 1.29E-50 | 1.53E-49 |
| **KCNC2** | -3.647969237 | 1.7144155 | 6.41E-32 | 3.57E-31 |
| **G0S2** | -3.651813549 | 9.008665474 | 6.84E-50 | 7.85E-49 |
| **PDE2A** | -3.655582038 | 4.414271911 | 1.52E-144 | 2.5E-142 |
| **NOVA1** | -3.657322766 | 3.413994054 | 3.4E-133 | 4.41E-131 |
| **TRIM63** | -3.67086421 | 2.160976293 | 2.39E-19 | 7.66E-19 |
| **HRC** | -3.6741844 | 2.900863081 | 4.54E-41 | 3.64E-40 |
| **NPR 1.00** | -3.679730065 | 5.65900064 | 1.93E-97 | 1.1E-95 |
| **SLC7A10** | -3.685353938 | 4.035277 | 4.29E-38 | 3.05E-37 |
| **CASQ2** | -3.702206183 | 3.92273591 | 2.98E-48 | 3.2E-47 |
| **MAP1LC3C** | -3.703942096 | 3.703087868 | 3.47E-81 | 1.15E-79 |
| **SORBS1** | -3.711132952 | 6.997218292 | 3.4E-90 | 1.5E-88 |
| **ABCA10** | -3.722086683 | 2.88309461 | 9.13E-140 | 1.38E-137 |
| **FGF10** | -3.729184199 | 3.693969891 | 5.33E-128 | 5.91E-126 |
| **SCN4A** | -3.753100219 | 2.927866103 | 2.01E-58 | 3.17E-57 |
| **FAM180B** | -3.758201506 | 2.834940239 | 3.52E-47 | 3.58E-46 |
| **RPL3L** | -3.770051585 | 2.939797742 | 2.44E-16 | 6.84E-16 |
| **GALNT15** | -3.776772622 | 5.116130401 | 3.54E-86 | 1.39E-84 |
| **SCN2B** | -3.781583486 | 2.307789714 | 2.38E-107 | 1.8E-105 |
| **PI16** | -3.823339555 | 6.692134684 | 3.23E-43 | 2.82E-42 |
| **SMPX** | -3.83211393 | 3.20549764 | 8.08E-13 | 1.91E-12 |
| **MYLK2** | -3.846704423 | 2.664940671 | 5.61E-17 | 1.62E-16 |
| **DPT** | -3.857034626 | 7.154680075 | 2.02E-72 | 5.17E-71 |
| **MYH11** | -3.859695472 | 6.066352232 | 6.88E-79 | 2.14E-77 |
| **ASPA** | -3.87776225 | 2.837464607 | 6.44E-82 | 2.21E-80 |
| **PAMR1** | -3.886296527 | 5.321095783 | 4.63E-132 | 5.84E-130 |
| **SPX** | -3.897459074 | 3.698305641 | 4.6E-35 | 2.92E-34 |
| **RPS4Y1** | -3.908440647 | 1.855685335 | 5.36E-14 | 1.34E-13 |
| **OGN** | -3.92605003 | 6.076246012 | 1.72E-64 | 3.38E-63 |
| **SVEP1** | -3.943183453 | 5.186899014 | 1.57E-107 | 1.2E-105 |
| **LPL** | -3.952834804 | 9.060699045 | 1.92E-55 | 2.73E-54 |
| **ITIH2** | -3.954394888 | 2.685870738 | 4.05E-45 | 3.8E-44 |
| **GHR** | -3.955874519 | 5.60008207 | 6.47E-89 | 2.74E-87 |
| **GYG2** | -3.959861504 | 7.200196098 | 1.39E-74 | 3.85E-73 |
| **GDF10** | -3.962964083 | 3.735654105 | 9.11E-86 | 3.51E-84 |
| **CLDN19** | -4.001348276 | 2.80402162 | 7.15E-82 | 2.45E-80 |
| **SRL** | -4.002533328 | 2.774236952 | 3.33E-31 | 1.81E-30 |
| **CRHBP** | -4.010925734 | 2.868525192 | 7.81E-61 | 1.33E-59 |
| **GLRA3** | -4.012290352 | 2.974861343 | 1.53E-26 | 6.76E-26 |
| **LRRN4CL** | -4.014710923 | 4.46836184 | 2.58E-116 | 2.33E-114 |
| **HBA1** | -4.021183744 | 2.127382614 | 1.31E-31 | 7.19E-31 |
| **AGTR1** | -4.035115332 | 4.03975166 | 1.28E-108 | 9.97E-107 |
| **MYOZ1** | -4.044534803 | 6.289730762 | 7.84E-24 | 3.08E-23 |
| **NNAT** | -4.046916289 | 4.980243808 | 5.03E-60 | 8.24E-59 |
| **MAOA** | -4.074633743 | 6.56454282 | 1.11E-64 | 2.19E-63 |
| **MLXIPL** | -4.076738947 | 3.943676047 | 4.47E-65 | 9E-64 |
| **AMPD1** | -4.080243886 | 3.010187328 | 8.43E-33 | 4.87E-32 |
| **CMYA5** | -4.080535868 | 4.973345457 | 2.27E-45 | 2.15E-44 |
| **ADH1A** | -4.101970017 | 1.990091524 | 4.47E-45 | 4.18E-44 |
| **CYP2A6** | -4.102976399 | 2.673275731 | 9.58E-58 | 1.47E-56 |
| **FOSB** | -4.105077772 | 7.90664201 | 1.17E-61 | 2.06E-60 |
| **ARHGAP36** | -4.109799127 | 3.553697964 | 3.28E-28 | 1.55E-27 |
| **GPIHBP1** | -4.111251881 | 5.21237634 | 2.3E-85 | 8.76E-84 |
| **MYMX** | -4.125224888 | 3.616368948 | 1.16E-60 | 1.96E-59 |
| **CYP1A1** | -4.130731398 | 2.235526585 | 1.19E-24 | 4.83E-24 |
| **FBP2** | -4.186626509 | 2.405831776 | 1.47E-17 | 4.33E-17 |
| **PPDPFL** | -4.195689505 | 2.121614666 | 4.21E-13 | 1.01E-12 |
| **SYNPO2L** | -4.212375596 | 2.201040135 | 1.7E-26 | 7.49E-26 |
| **MMRN1** | -4.223779332 | 4.610797681 | 3.74E-90 | 1.65E-88 |
| **CHRDL1** | -4.225655403 | 7.498152664 | 1.28E-83 | 4.68E-82 |
| **RDH5** | -4.22861552 | 3.074945503 | 7.24E-71 | 1.78E-69 |
| **ITIH5** | -4.253537206 | 5.580910486 | 7.22E-111 | 5.83E-109 |
| **CFD** | -4.267545068 | 8.798971049 | 4.44E-72 | 1.12E-70 |
| **ABCA9** | -4.2724476 | 3.777692572 | 9.84E-137 | 1.38E-134 |
| **CES1** | -4.319603287 | 6.772756253 | 7.13E-63 | 1.31E-61 |
| **SMYD1** | -4.319719179 | 3.976939904 | 1.06E-47 | 1.11E-46 |
| **PDK4** | -4.328369383 | 8.031266267 | 2.99E-90 | 1.33E-88 |
| **ADGRD2** | -4.348544875 | 2.309887086 | 8.53E-51 | 1.02E-49 |
| **ACSM5** | -4.348604787 | 3.489047901 | 5.19E-77 | 1.52E-75 |
| **APOB** | -4.389644836 | 2.056827313 | 1.52E-45 | 1.45E-44 |
| **ALDH1L1** | -4.424767699 | 3.399693464 | 6.01E-64 | 1.16E-62 |
| **CA3** | -4.444139473 | 7.121964271 | 3.46E-30 | 1.79E-29 |
| **CLEC3B** | -4.459488726 | 6.67412487 | 2.08E-100 | 1.32E-98 |
| **MYBPC2** | -4.465836312 | 4.183265694 | 8.97E-17 | 2.56E-16 |
| **ACACB** | -4.466993418 | 5.738833043 | 3.66E-117 | 3.33E-115 |
| **MYPN** | -4.479426224 | 2.072100594 | 1.74E-15 | 4.67E-15 |
| **MYOM1** | -4.485507295 | 4.491246393 | 1.35E-64 | 2.67E-63 |
| **SLC26A3** | -4.51468156 | 3.341949834 | 8.17E-33 | 4.73E-32 |
| **AOC3** | -4.521087305 | 7.885362987 | 3.02E-105 | 2.12E-103 |
| **ENO3** | -4.534537787 | 5.667553207 | 2.78E-33 | 1.64E-32 |
| **SERPINA6** | -4.544832303 | 3.689900941 | 1.27E-31 | 6.96E-31 |
| **GSTM5** | -4.578130391 | 4.361350127 | 4.21E-64 | 8.18E-63 |
| **LYVE1** | -4.59476483 | 5.925396043 | 4.62E-91 | 2.12E-89 |
| **NMRK2** | -4.615770637 | 1.999780655 | 5.62E-20 | 1.85E-19 |
| **HBA2** | -4.622316914 | 6.551475361 | 7.43E-51 | 8.9E-50 |
| **ANGPTL8** | -4.638931007 | 2.864151881 | 4.86E-55 | 6.81E-54 |
| **BTNL9** | -4.648587267 | 5.392627855 | 2.08E-127 | 2.25E-125 |
| **HEPACAM** | -4.653480244 | 2.570023819 | 5.09E-63 | 9.47E-62 |
| **ZBTB16** | -4.679373941 | 4.527139014 | 2.47E-102 | 1.64E-100 |
| **CAVIN2** | -4.708559138 | 6.416842756 | 2.36E-150 | 4.31E-148 |
| **SLC2A4** | -4.717767926 | 4.220001839 | 3.42E-79 | 1.08E-77 |
| **TNXB** | -4.718882792 | 5.200100449 | 1.89E-131 | 2.32E-129 |
| **ABCA8** | -4.725478605 | 4.44798416 | 5.08E-131 | 6.09E-129 |
| **PFKFB1** | -4.732037811 | 4.111700308 | 8.19E-85 | 3.07E-83 |
| **LVRN** | -4.736385049 | 2.978677748 | 3.44E-62 | 6.17E-61 |
| **HSD17B13** | -4.744751582 | 3.071188066 | 1.58E-65 | 3.22E-64 |
| **GPAM** | -4.752617917 | 6.933706349 | 8.9E-114 | 7.71E-112 |
| **MYOZ2** | -4.769638937 | 2.22779633 | 2.87E-31 | 1.56E-30 |
| **LGALS12** | -4.770594673 | 5.751856018 | 1.01E-54 | 1.4E-53 |
| **SCARA5** | -4.796091893 | 5.742062265 | 4.78E-91 | 2.19E-89 |
| **TTN** | -4.801405958 | 2.519099616 | 1.07E-31 | 5.89E-31 |
| **LMOD3** | -4.807948118 | 2.689384075 | 3.99E-28 | 1.88E-27 |
| **NPY2R** | -4.849604097 | 3.259361344 | 1.64E-55 | 2.34E-54 |
| **HSPB6** | -4.852918819 | 5.875704784 | 2.2E-81 | 7.42E-80 |
| **OXTR** | -4.900593288 | 6.706262779 | 2.52E-91 | 1.18E-89 |
| **KLB** | -4.909166846 | 4.122542297 | 2.18E-84 | 8.07E-83 |
| **SLC19A3** | -4.916239844 | 5.331569963 | 2.22E-77 | 6.55E-76 |
| **VEGFD** | -5.014795989 | 4.98628802 | 1.34E-171 | 3.92E-169 |
| **RBP4** | -5.025421103 | 8.359909236 | 3.73E-60 | 6.13E-59 |
| **FABP4** | -5.04418426 | 11.21301799 | 6.35E-52 | 7.96E-51 |
| **SGCG** | -5.057003173 | 3.743270433 | 4.87E-70 | 1.16E-68 |
| **CD36** | -5.061135675 | 8.127883521 | 1.01E-101 | 6.66E-100 |
| **CIDEA** | -5.079216598 | 6.50875282 | 2.97E-47 | 3.04E-46 |
| **ALB** | -5.101400442 | 5.521119045 | 2.76E-43 | 2.41E-42 |
| **LIPE** | -5.14212985 | 7.301801828 | 1.42E-83 | 5.15E-82 |
| **PPP1R27** | -5.161587991 | 2.872504135 | 1.49E-20 | 5.07E-20 |
| **TAT** | -5.227040046 | 5.54753573 | 1.91E-45 | 1.81E-44 |
| **AL845331.1** | -5.274595549 | 2.879971974 | 1.41E-51 | 1.75E-50 |
| **FHL1** | -5.281479843 | 8.728041656 | 1.47E-98 | 8.87E-97 |
| **HBB** | -5.327267418 | 8.517428134 | 4.39E-68 | 9.92E-67 |
| **ACVR1C** | -5.32773923 | 4.250767363 | 2.06E-86 | 8.16E-85 |
| **DES** | -5.370375316 | 7.256065222 | 1.06E-35 | 6.89E-35 |
| **SFTPB** | -5.375155489 | 3.367965821 | 6.4E-22 | 2.31E-21 |
| **KCNIP2** | -5.376690901 | 5.968152056 | 4E-99 | 2.45E-97 |
| **C14orf180** | -5.379435448 | 3.408498997 | 1.53E-62 | 2.77E-61 |
| **TMEM132C** | -5.393840136 | 4.561639806 | 3.55E-101 | 2.31E-99 |
| **MYH1** | -5.461712145 | 2.605792103 | 5.6E-37 | 3.83E-36 |
| **TNNI1** | -5.500368415 | 4.625828819 | 2.54E-36 | 1.69E-35 |
| **MYL3** | -5.518185235 | 4.269465479 | 8.1E-44 | 7.23E-43 |
| **ACTN2** | -5.549875203 | 5.728203182 | 9.41E-19 | 2.92E-18 |
| **TIMP4** | -5.584825596 | 7.242264876 | 3.39E-82 | 1.17E-80 |
| **KLHL40** | -5.586435686 | 2.009666728 | 2.94E-16 | 8.2E-16 |
| **ABRA** | -5.631569177 | 2.212122783 | 1.63E-22 | 6.05E-22 |
| **PPP1R3A** | -5.649258982 | 2.002809614 | 3.19E-14 | 8.09E-14 |
| **HSPB7** | -5.694004443 | 6.403901366 | 2.43E-87 | 9.9E-86 |
| **PCK1** | -5.695541488 | 4.722662167 | 1.45E-63 | 2.76E-62 |
| **HJV** | -5.708562358 | 2.25102495 | 4.25E-19 | 1.34E-18 |
| **LACRT** | -5.712123418 | 3.039076928 | 8.19E-29 | 3.97E-28 |
| **AQP7** | -5.736057815 | 5.537466104 | 7.54E-89 | 3.17E-87 |
| **PLIN4** | -5.780569266 | 9.264785926 | 4.21E-70 | 1.01E-68 |
| **ACTN3** | -5.817258695 | 3.077691071 | 3.64E-22 | 1.33E-21 |
| **ADH1B** | -5.825608766 | 7.920560979 | 1.45E-70 | 3.53E-69 |
| **LDB3** | -5.864636808 | 4.539202387 | 1.68E-47 | 1.73E-46 |
| **ADIPOQ** | -5.870070887 | 8.533431879 | 4.36E-59 | 7E-58 |
| **CD300LG** | -5.890699795 | 5.19898722 | 2.19E-173 | 6.95E-171 |
| **TRDN** | -5.8959857 | 3.28943379 | 4.16E-36 | 2.76E-35 |
| **GPD1** | -6.023252339 | 8.52519771 | 3.67E-80 | 1.19E-78 |
| **CA4** | -6.032427474 | 4.347601111 | 7.73E-131 | 9.12E-129 |
| **MYF6** | -6.058644714 | 2.550120072 | 1.21E-23 | 4.7E-23 |
| **C10orf71** | -6.067649375 | 2.427889875 | 1.13E-14 | 2.92E-14 |
| **TRARG1** | -6.073800298 | 7.289544299 | 1.45E-72 | 3.73E-71 |
| **ACTA1** | -6.132766285 | 10.13994233 | 2.4E-23 | 9.19E-23 |
| **APOBEC2** | -6.163773722 | 4.140975647 | 1.43E-36 | 9.66E-36 |
| **CIDEC** | -6.166631708 | 8.263145339 | 2.6E-75 | 7.31E-74 |
| **NEB** | -6.174138452 | 4.160092556 | 3.33E-31 | 1.8E-30 |
| **PLIN1** | -6.214292526 | 9.201386798 | 5.19E-74 | 1.41E-72 |
| **CACNA1S** | -6.260584403 | 2.476177504 | 1.49E-18 | 4.59E-18 |
| **ATP1A2** | -6.2797679 | 5.113254133 | 3.03E-99 | 1.86E-97 |
| **ATP2A1** | -6.284069789 | 5.715645745 | 2.4E-36 | 1.6E-35 |
| **TNNC1** | -6.341410518 | 6.940137118 | 7.85E-35 | 4.93E-34 |
| **MYOT** | -6.343244726 | 3.244600831 | 7.46E-26 | 3.19E-25 |
| **GLYAT** | -6.398732466 | 4.136783888 | 2.54E-96 | 1.4E-94 |
| **COX6A2** | -6.418360972 | 4.078330478 | 2.31E-20 | 7.76E-20 |
| **TNNC2** | -6.545437397 | 7.498789235 | 6.19E-44 | 5.54E-43 |
| **CASQ1** | -6.651734347 | 5.415276057 | 1.22E-45 | 1.17E-44 |
| **DEFB132** | -6.70177121 | 4.017569889 | 3.83E-74 | 1.05E-72 |
| **MYLPF** | -6.735542355 | 6.599827952 | 5.07E-34 | 3.08E-33 |
| **SLN** | -7.009402269 | 6.149707008 | 1.68E-26 | 7.42E-26 |
| **PYGM** | -7.121079783 | 5.947805039 | 1.45E-60 | 2.43E-59 |
| **STRIT1** | -7.161444139 | 4.051716614 | 2.28E-23 | 8.78E-23 |
| **LEP** | -7.280250923 | 7.679272373 | 2.43E-95 | 1.3E-93 |
| **KLHL41** | -7.377979864 | 5.65459094 | 8.8E-43 | 7.56E-42 |
| **TCAP** | -7.503655756 | 6.967878883 | 5.9E-45 | 5.5E-44 |
| **MYOC** | -7.566122408 | 4.715265316 | 7.19E-82 | 2.45E-80 |
| **LMOD2** | -7.997319898 | 4.548455937 | 3.63E-23 | 1.38E-22 |
| **XIRP2** | -8.024859867 | 4.042555062 | 2.53E-17 | 7.41E-17 |
| **NRAP** | -8.376495404 | 5.02101474 | 1.42E-24 | 5.73E-24 |
| **CSRP3** | -8.93550413 | 5.261220626 | 2.96E-20 | 9.9E-20 |
| **MYH7** | -9.368389143 | 7.229677495 | 2.02E-21 | 7.16E-21 |
| **CKM** | -9.673941055 | 9.038799659 | 4.54E-35 | 2.88E-34 |
| **MYL2** | -9.853898292 | 7.979150448 | 5.11E-31 | 2.75E-30 |
| **MYH2** | -10.67028322 | 7.271283055 | 3.01E-32 | 1.7E-31 |
| **MYL1** | -10.7002895 | 7.557064318 | 4.28E-18 | 1.29E-17 |
