# Supplementary material for: Integrating Bulk and Single-cell RNA-seq to Construct a Macrophage-related Prognostic Model for Prognostic Stratification in Triple-negative Breast Cancer
Source: J Cancer. 2024 Sep 23;15(18):6002–15. doi: 10.7150/jca.101042 (PMC11493015; doi:10.7150/jca.101042)
Supplement: Supplementary file 1 — Supplementary figure and tables. [file jcav15p6002s1.zip › Supplementary File/Table S3 Macrophage differential.docx]

| **Table S3 Macrophage differential-related genes** | | | | | | |
| --- | --- | --- | --- | --- | --- | --- |
|  | status | family | pval | qval | gene_short_name | use_for_ordering |
| **SPP1** | OK | negbinomial.size | 1.71E-143 | 3.82E-139 | SPP1 | TRUE |
| **CCL3L3** | OK | negbinomial.size | 5.42E-142 | 6.07E-138 | CCL3L3 | TRUE |
| **FABP5** | OK | negbinomial.size | 2.89E-139 | 2.16E-135 | FABP5 | TRUE |
| **PTGDS** | OK | negbinomial.size | 2.21E-128 | 1.24E-124 | PTGDS | TRUE |
| **FABP4** | OK | negbinomial.size | 5.95E-128 | 2.66E-124 | FABP4 | TRUE |
| **CCL4L2** | OK | negbinomial.size | 1.23E-125 | 4.59E-122 | CCL4L2 | TRUE |
| **IGKC** | OK | negbinomial.size | 1.12E-124 | 3.59E-121 | IGKC | TRUE |
| **CXCL8** | OK | negbinomial.size | 2.91E-113 | 8.15E-110 | CXCL8 | TRUE |
| **APOC1** | OK | negbinomial.size | 1.71E-100 | 4.25E-97 | APOC1 | TRUE |
| **APOE** | OK | negbinomial.size | 3.27E-76 | 7.31E-73 | APOE | TRUE |
| **RNASE1** | OK | negbinomial.size | 1.28E-74 | 2.6E-71 | RNASE1 | TRUE |
| **IL1B** | OK | negbinomial.size | 2.62E-73 | 4.89E-70 | IL1B | TRUE |
| **HLA-DRB5** | OK | negbinomial.size | 7.72E-71 | 1.33E-67 | HLA-DRB5 | TRUE |
| **RGS1** | OK | negbinomial.size | 1.52E-70 | 2.43E-67 | RGS1 | TRUE |
| **CCL3** | OK | negbinomial.size | 7.07E-69 | 1.06E-65 | CCL3 | TRUE |
| **SCGB2A2** | OK | negbinomial.size | 2.77E-68 | 3.87E-65 | SCGB2A2 | TRUE |
| **LYZ** | OK | negbinomial.size | 1.3E-62 | 1.71E-59 | LYZ | TRUE |
| **CCL4** | OK | negbinomial.size | 6.31E-62 | 7.85E-59 | CCL4 | TRUE |
| **LAIR2** | OK | negbinomial.size | 4.54E-61 | 5.35E-58 | LAIR2 | TRUE |
| **CXCL10** | OK | negbinomial.size | 3.55E-59 | 3.97E-56 | CXCL10 | TRUE |
| **CTSL** | OK | negbinomial.size | 2.47E-58 | 2.63E-55 | CTSL | TRUE |
| **S100A6** | OK | negbinomial.size | 5.13E-47 | 5.21E-44 | S100A6 | TRUE |
| **RGS2** | OK | negbinomial.size | 7.45E-44 | 7.25E-41 | RGS2 | TRUE |
| **ACP5** | OK | negbinomial.size | 5.57E-42 | 5.19E-39 | ACP5 | TRUE |
| **MMP9** | OK | negbinomial.size | 8.79E-42 | 7.87E-39 | MMP9 | TRUE |
| **RP11-1143G9.4** | OK | negbinomial.size | 1.33E-41 | 1.15E-38 | RP11-1143G9.4 | TRUE |
| **IER3** | OK | negbinomial.size | 1.14E-39 | 9.48E-37 | IER3 | TRUE |
| **CCL18** | OK | negbinomial.size | 1.55E-39 | 1.24E-36 | CCL18 | TRUE |
| **CXCR4** | OK | negbinomial.size | 1.51E-38 | 1.17E-35 | CXCR4 | TRUE |
| **IGLC3** | OK | negbinomial.size | 1.77E-38 | 1.32E-35 | IGLC3 | TRUE |
| **APOD** | OK | negbinomial.size | 1.56E-37 | 1.13E-34 | APOD | TRUE |
| **IGHG1** | OK | negbinomial.size | 1.64E-37 | 1.15E-34 | IGHG1 | TRUE |
| **VIM** | OK | negbinomial.size | 2.78E-36 | 1.89E-33 | VIM | TRUE |
| **GPR183** | OK | negbinomial.size | 8.89E-36 | 5.86E-33 | GPR183 | TRUE |
| **PRDX1** | OK | negbinomial.size | 9.28E-36 | 5.94E-33 | PRDX1 | TRUE |
| **HERPUD1** | OK | negbinomial.size | 2.31E-35 | 1.44E-32 | HERPUD1 | TRUE |
| **MT-ND3** | OK | negbinomial.size | 6.28E-35 | 3.8E-32 | MT-ND3 | TRUE |
| **ANXA2** | OK | negbinomial.size | 9.93E-35 | 5.85E-32 | ANXA2 | TRUE |
| **CCL5** | OK | negbinomial.size | 1.42E-32 | 8.16E-30 | CCL5 | TRUE |
| **CXCL9** | OK | negbinomial.size | 2.34E-32 | 1.31E-29 | CXCL9 | TRUE |
| **MT-CO1** | OK | negbinomial.size | 3.54E-32 | 1.93E-29 | MT-CO1 | TRUE |
| **GPR34** | OK | negbinomial.size | 4.08E-32 | 2.18E-29 | GPR34 | TRUE |
| **IFI27** | OK | negbinomial.size | 4.45E-32 | 2.32E-29 | IFI27 | TRUE |
| **ZFP36** | OK | negbinomial.size | 5.13E-32 | 2.61E-29 | ZFP36 | TRUE |
| **S100A10** | OK | negbinomial.size | 7.61E-31 | 3.79E-28 | S100A10 | TRUE |
| **SEPP1** | OK | negbinomial.size | 1.95E-30 | 9.48E-28 | SEPP1 | TRUE |
| **RPS27L** | OK | negbinomial.size | 6.42E-30 | 3.06E-27 | RPS27L | TRUE |
| **CSTB** | OK | negbinomial.size | 7.84E-30 | 3.66E-27 | CSTB | TRUE |
| **CHI3L1** | OK | negbinomial.size | 1.3E-29 | 5.95E-27 | CHI3L1 | TRUE |
| **MT-ATP6** | OK | negbinomial.size | 3.22E-29 | 1.44E-26 | MT-ATP6 | TRUE |
| **CXCL11** | OK | negbinomial.size | 5.44E-29 | 2.39E-26 | CXCL11 | TRUE |
| **TXN** | OK | negbinomial.size | 5.83E-29 | 2.51E-26 | TXN | TRUE |
| **MT-CO2** | OK | negbinomial.size | 7.54E-29 | 3.18E-26 | MT-CO2 | TRUE |
| **IGLC2** | OK | negbinomial.size | 8.48E-29 | 3.52E-26 | IGLC2 | TRUE |
| **DNAJB1** | OK | negbinomial.size | 1.5E-28 | 6.11E-26 | DNAJB1 | TRUE |
| **G0S2** | OK | negbinomial.size | 7.81E-28 | 3.12E-25 | G0S2 | TRUE |
| **MARCO** | OK | negbinomial.size | 8.04E-28 | 3.16E-25 | MARCO | TRUE |
| **CD52** | OK | negbinomial.size | 8.63E-28 | 3.33E-25 | CD52 | TRUE |
| **C10orf54** | OK | negbinomial.size | 2.17E-27 | 8.22E-25 | C10orf54 | TRUE |
| **FN1** | OK | negbinomial.size | 2.63E-27 | 9.81E-25 | FN1 | TRUE |
| **PDK4** | OK | negbinomial.size | 2.97E-27 | 1.09E-24 | PDK4 | TRUE |
| **HSPA6** | OK | negbinomial.size | 3.49E-27 | 1.26E-24 | HSPA6 | TRUE |
| **PSAP** | OK | negbinomial.size | 1.65E-26 | 5.87E-24 | PSAP | TRUE |
| **GCHFR** | OK | negbinomial.size | 1.48E-25 | 5.19E-23 | GCHFR | TRUE |
| **CD36** | OK | negbinomial.size | 2.48E-25 | 8.55E-23 | CD36 | TRUE |
| **RP11-480C22.1** | OK | negbinomial.size | 2.66E-25 | 9.02E-23 | RP11-480C22.1 | TRUE |
| **CHIT1** | OK | negbinomial.size | 6.81E-25 | 2.27E-22 | CHIT1 | TRUE |
| **MS4A7** | OK | negbinomial.size | 8.12E-25 | 2.67E-22 | MS4A7 | TRUE |
| **FOS** | OK | negbinomial.size | 1.88E-24 | 6.11E-22 | FOS | TRUE |
| **LGALS3** | OK | negbinomial.size | 2.68E-24 | 8.56E-22 | LGALS3 | TRUE |
| **GPNMB** | OK | negbinomial.size | 5.08E-24 | 1.6E-21 | GPNMB | TRUE |
| **CH25H** | OK | negbinomial.size | 6.73E-24 | 2.09E-21 | CH25H | TRUE |
| **HLA-DPB1** | OK | negbinomial.size | 8.8E-24 | 2.7E-21 | HLA-DPB1 | TRUE |
| **LGALS2** | OK | negbinomial.size | 8.93E-24 | 2.7E-21 | LGALS2 | TRUE |
| **S100A4** | OK | negbinomial.size | 1.46E-23 | 4.36E-21 | S100A4 | TRUE |
| **CHCHD2** | OK | negbinomial.size | 3.35E-23 | 9.88E-21 | CHCHD2 | TRUE |
| **MMP19** | OK | negbinomial.size | 6.38E-23 | 1.85E-20 | MMP19 | TRUE |
| **CTSZ** | OK | negbinomial.size | 6.49E-23 | 1.86E-20 | CTSZ | TRUE |
| **C3** | OK | negbinomial.size | 1.07E-22 | 3.03E-20 | C3 | TRUE |
| **SDS** | OK | negbinomial.size | 2.72E-22 | 7.62E-20 | SDS | TRUE |
| **FBP1** | OK | negbinomial.size | 3.23E-22 | 8.92E-20 | FBP1 | TRUE |
| **TSC22D3** | OK | negbinomial.size | 5.17E-22 | 1.41E-19 | TSC22D3 | TRUE |
| **LGMN** | OK | negbinomial.size | 7.81E-22 | 2.11E-19 | LGMN | TRUE |
| **HSP90AA1** | OK | negbinomial.size | 9E-22 | 2.4E-19 | HSP90AA1 | TRUE |
| **KLF6** | OK | negbinomial.size | 1.18E-21 | 3.1E-19 | KLF6 | TRUE |
| **SH3BGRL3** | OK | negbinomial.size | 1.3E-21 | 3.4E-19 | SH3BGRL3 | TRUE |
| **DNAJB6** | OK | negbinomial.size | 1.38E-21 | 3.54E-19 | DNAJB6 | TRUE |
| **RPS26** | OK | negbinomial.size | 1.73E-21 | 4.4E-19 | RPS26 | TRUE |
| **CAPG** | OK | negbinomial.size | 1.87E-21 | 4.7E-19 | CAPG | TRUE |
| **CEBPB** | OK | negbinomial.size | 1.94E-21 | 4.82E-19 | CEBPB | TRUE |
| **MT1G** | OK | negbinomial.size | 2.53E-21 | 6.23E-19 | MT1G | TRUE |
| **MT-ND1** | OK | negbinomial.size | 3.01E-21 | 7.33E-19 | MT-ND1 | TRUE |
| **CXCL2** | OK | negbinomial.size | 3.99E-21 | 9.61E-19 | CXCL2 | TRUE |
| **HLA-A** | OK | negbinomial.size | 7.71E-21 | 1.84E-18 | HLA-A | TRUE |
| **HLA-DQA2** | OK | negbinomial.size | 7.93E-21 | 1.87E-18 | HLA-DQA2 | TRUE |
| **LIPA** | OK | negbinomial.size | 8.21E-21 | 1.91E-18 | LIPA | TRUE |
| **LPL** | OK | negbinomial.size | 1E-20 | 2.32E-18 | LPL | TRUE |
| **ATOX1** | OK | negbinomial.size | 1.14E-20 | 2.6E-18 | ATOX1 | TRUE |
| **H3F3B** | OK | negbinomial.size | 1.16E-20 | 2.63E-18 | H3F3B | TRUE |
| **HSPE1** | OK | negbinomial.size | 1.42E-20 | 3.18E-18 | HSPE1 | TRUE |
| **CYBA** | OK | negbinomial.size | 1.44E-20 | 3.18E-18 | CYBA | TRUE |
| **IER2** | OK | negbinomial.size | 1.92E-20 | 4.21E-18 | IER2 | TRUE |
| **MT-ND2** | OK | negbinomial.size | 3.18E-20 | 6.91E-18 | MT-ND2 | TRUE |
| **ZFAND2A** | OK | negbinomial.size | 3.85E-20 | 8.28E-18 | ZFAND2A | TRUE |
| **PPP1R15A** | OK | negbinomial.size | 5.71E-20 | 1.22E-17 | PPP1R15A | TRUE |
| **CD40** | OK | negbinomial.size | 8.89E-20 | 1.88E-17 | CD40 | TRUE |
| **CD83** | OK | negbinomial.size | 5.79E-19 | 1.21E-16 | CD83 | TRUE |
| **IGSF6** | OK | negbinomial.size | 6E-19 | 1.24E-16 | IGSF6 | TRUE |
| **CREM** | OK | negbinomial.size | 7.3E-19 | 1.5E-16 | CREM | TRUE |
| **FPR3** | OK | negbinomial.size | 1.25E-18 | 2.55E-16 | FPR3 | TRUE |
| **MT-ND4** | OK | negbinomial.size | 1.34E-18 | 2.7E-16 | MT-ND4 | TRUE |
| **CTSD** | OK | negbinomial.size | 2.41E-18 | 4.82E-16 | CTSD | TRUE |
| **HSPB1** | OK | negbinomial.size | 4.99E-18 | 9.88E-16 | HSPB1 | TRUE |
| **TGFBI** | OK | negbinomial.size | 6.39E-18 | 1.25E-15 | TGFBI | TRUE |
| **DCN** | OK | negbinomial.size | 8.63E-18 | 1.68E-15 | DCN | TRUE |
| **PHACTR1** | OK | negbinomial.size | 1E-17 | 1.93E-15 | PHACTR1 | TRUE |
| **SLC40A1** | OK | negbinomial.size | 1.07E-17 | 2.05E-15 | SLC40A1 | TRUE |
| **PMAIP1** | OK | negbinomial.size | 1.42E-17 | 2.69E-15 | PMAIP1 | TRUE |
| **IL4I1** | OK | negbinomial.size | 1.76E-17 | 3.32E-15 | IL4I1 | TRUE |
| **IL32** | OK | negbinomial.size | 2.42E-17 | 4.51E-15 | IL32 | TRUE |
| **CCL8** | OK | negbinomial.size | 2.62E-17 | 4.85E-15 | CCL8 | TRUE |
| **PSME2** | OK | negbinomial.size | 2.67E-17 | 4.91E-15 | PSME2 | TRUE |
| **CD69** | OK | negbinomial.size | 2.98E-17 | 5.42E-15 | CD69 | TRUE |
| **PLA2G7** | OK | negbinomial.size | 4E-17 | 7.17E-15 | PLA2G7 | TRUE |
| **HSPA1A** | OK | negbinomial.size | 4.01E-17 | 7.17E-15 | HSPA1A | TRUE |
| **CTSH** | OK | negbinomial.size | 9.95E-17 | 1.77E-14 | CTSH | TRUE |
| **MT-CO3** | OK | negbinomial.size | 1.46E-16 | 2.57E-14 | MT-CO3 | TRUE |
| **IGHG3** | OK | negbinomial.size | 1.6E-16 | 2.81E-14 | IGHG3 | TRUE |
| **MT-CYB** | OK | negbinomial.size | 2.33E-16 | 4.04E-14 | MT-CYB | TRUE |
| **LAMP1** | OK | negbinomial.size | 2.57E-16 | 4.42E-14 | LAMP1 | TRUE |
| **PLIN2** | OK | negbinomial.size | 2.98E-16 | 5.09E-14 | PLIN2 | TRUE |
| **FTL** | OK | negbinomial.size | 3.26E-16 | 5.53E-14 | FTL | TRUE |
| **SAT1** | OK | negbinomial.size | 3.73E-16 | 6.28E-14 | SAT1 | TRUE |
| **LAIR1** | OK | negbinomial.size | 3.79E-16 | 6.33E-14 | LAIR1 | TRUE |
| **BTG2** | OK | negbinomial.size | 4.55E-16 | 7.55E-14 | BTG2 | TRUE |
| **TRA2B** | OK | negbinomial.size | 4.89E-16 | 8.05E-14 | TRA2B | TRUE |
| **CCL7** | OK | negbinomial.size | 4.98E-16 | 8.14E-14 | CCL7 | TRUE |
| **S100A8** | OK | negbinomial.size | 5.05E-16 | 8.2E-14 | S100A8 | TRUE |
| **TMEM176B** | OK | negbinomial.size | 1.19E-15 | 1.92E-13 | TMEM176B | TRUE |
| **LGALS1** | OK | negbinomial.size | 1.36E-15 | 2.17E-13 | LGALS1 | TRUE |
| **NR1H3** | OK | negbinomial.size | 1.57E-15 | 2.49E-13 | NR1H3 | TRUE |
| **MGP** | OK | negbinomial.size | 1.69E-15 | 2.66E-13 | MGP | TRUE |
| **CXCL12** | OK | negbinomial.size | 4.41E-15 | 6.91E-13 | CXCL12 | TRUE |
| **ELF1** | OK | negbinomial.size | 4.61E-15 | 7.16E-13 | ELF1 | TRUE |
| **EGR1** | OK | negbinomial.size | 8.44E-15 | 1.3E-12 | EGR1 | TRUE |
| **HSPD1** | OK | negbinomial.size | 9.78E-15 | 1.5E-12 | HSPD1 | TRUE |
| **TPT1** | OK | negbinomial.size | 1.02E-14 | 1.56E-12 | TPT1 | TRUE |
| **HES1** | OK | negbinomial.size | 1.24E-14 | 1.87E-12 | HES1 | TRUE |
| **HSP90AB1** | OK | negbinomial.size | 1.38E-14 | 2.07E-12 | HSP90AB1 | TRUE |
| **MT1H** | OK | negbinomial.size | 2.01E-14 | 3E-12 | MT1H | TRUE |
| **HBB** | OK | negbinomial.size | 2.14E-14 | 3.18E-12 | HBB | TRUE |
| **IL10** | OK | negbinomial.size | 2.25E-14 | 3.32E-12 | IL10 | TRUE |
| **EIF1** | OK | negbinomial.size | 3.38E-14 | 4.95E-12 | EIF1 | TRUE |
| **HLA-DRB1** | OK | negbinomial.size | 4.32E-14 | 6.28E-12 | HLA-DRB1 | TRUE |
| **PSMB9** | OK | negbinomial.size | 4.37E-14 | 6.31E-12 | PSMB9 | TRUE |
| **S100A9** | OK | negbinomial.size | 6.43E-14 | 9.22E-12 | S100A9 | TRUE |
| **CTSC** | OK | negbinomial.size | 9.53E-14 | 1.36E-11 | CTSC | TRUE |
| **RGCC** | OK | negbinomial.size | 9.95E-14 | 1.41E-11 | RGCC | TRUE |
| **IL1RN** | OK | negbinomial.size | 1.12E-13 | 1.58E-11 | IL1RN | TRUE |
| **ISG15** | OK | negbinomial.size | 1.62E-13 | 2.26E-11 | ISG15 | TRUE |
| **SNX10** | OK | negbinomial.size | 1.66E-13 | 2.3E-11 | SNX10 | TRUE |
| **S100A11** | OK | negbinomial.size | 4.07E-13 | 5.62E-11 | S100A11 | TRUE |
| **HMOX1** | OK | negbinomial.size | 4.15E-13 | 5.7E-11 | HMOX1 | TRUE |
| **EMP3** | OK | negbinomial.size | 4.48E-13 | 6.12E-11 | EMP3 | TRUE |
| **USP53** | OK | negbinomial.size | 4.83E-13 | 6.56E-11 | USP53 | TRUE |
| **JUND** | OK | negbinomial.size | 5.13E-13 | 6.91E-11 | JUND | TRUE |
| **C1QB** | OK | negbinomial.size | 5.21E-13 | 6.98E-11 | C1QB | TRUE |
| **MT-ND5** | OK | negbinomial.size | 7.07E-13 | 9.41E-11 | MT-ND5 | TRUE |
| **RILPL2** | OK | negbinomial.size | 1.15E-12 | 1.52E-10 | RILPL2 | TRUE |
| **FTH1** | OK | negbinomial.size | 1.33E-12 | 1.75E-10 | FTH1 | TRUE |
| **RPL13A** | OK | negbinomial.size | 1.37E-12 | 1.8E-10 | RPL13A | TRUE |
| **MSR1** | OK | negbinomial.size | 1.73E-12 | 2.25E-10 | MSR1 | TRUE |
| **NAMPT** | OK | negbinomial.size | 2.05E-12 | 2.65E-10 | NAMPT | TRUE |
| **C2** | OK | negbinomial.size | 2.55E-12 | 3.29E-10 | C2 | TRUE |
| **PDE4B** | OK | negbinomial.size | 2.8E-12 | 3.58E-10 | PDE4B | TRUE |
| **ADAMDEC1** | OK | negbinomial.size | 3.62E-12 | 4.6E-10 | ADAMDEC1 | TRUE |
| **HLA-DQB1** | OK | negbinomial.size | 4.44E-12 | 5.61E-10 | HLA-DQB1 | TRUE |
| **PPA1** | OK | negbinomial.size | 5.11E-12 | 6.43E-10 | PPA1 | TRUE |
| **GLIPR2** | OK | negbinomial.size | 5.83E-12 | 7.29E-10 | GLIPR2 | TRUE |
| **FCGR2A** | OK | negbinomial.size | 5.97E-12 | 7.42E-10 | FCGR2A | TRUE |
| **RHOB** | OK | negbinomial.size | 6.48E-12 | 8.01E-10 | RHOB | TRUE |
| **CCL2** | OK | negbinomial.size | 7.57E-12 | 9.31E-10 | CCL2 | TRUE |
| **TM4SF1** | OK | negbinomial.size | 8.6E-12 | 1.05E-09 | TM4SF1 | TRUE |
| **TIMP3** | OK | negbinomial.size | 8.64E-12 | 1.05E-09 | TIMP3 | TRUE |
| **FNIP2** | OK | negbinomial.size | 9.19E-12 | 1.11E-09 | FNIP2 | TRUE |
| **HSPH1** | OK | negbinomial.size | 9.61E-12 | 1.16E-09 | HSPH1 | TRUE |
| **ATF3** | OK | negbinomial.size | 9.69E-12 | 1.16E-09 | ATF3 | TRUE |
| **IGF1** | OK | negbinomial.size | 1.1E-11 | 1.32E-09 | IGF1 | TRUE |
| **PHLDA1** | OK | negbinomial.size | 1.33E-11 | 1.57E-09 | PHLDA1 | TRUE |
| **TNF** | OK | negbinomial.size | 1.53E-11 | 1.8E-09 | TNF | TRUE |
| **NR4A2** | OK | negbinomial.size | 1.55E-11 | 1.82E-09 | NR4A2 | TRUE |
| **IL6** | OK | negbinomial.size | 1.61E-11 | 1.88E-09 | IL6 | TRUE |
| **ATP6V1F** | OK | negbinomial.size | 1.7E-11 | 1.98E-09 | ATP6V1F | TRUE |
| **FCER1A** | OK | negbinomial.size | 1.8E-11 | 2.08E-09 | FCER1A | TRUE |
| **CD9** | OK | negbinomial.size | 1.88E-11 | 2.16E-09 | CD9 | TRUE |
| **GBP1** | OK | negbinomial.size | 2.34E-11 | 2.67E-09 | GBP1 | TRUE |
| **CTSK** | OK | negbinomial.size | 2.63E-11 | 2.99E-09 | CTSK | TRUE |
| **C15orf48** | OK | negbinomial.size | 2.93E-11 | 3.3E-09 | C15orf48 | TRUE |
| **TCHH** | OK | negbinomial.size | 2.94E-11 | 3.3E-09 | TCHH | TRUE |
| **PLAUR** | OK | negbinomial.size | 3.15E-11 | 3.52E-09 | PLAUR | TRUE |
| **UBE2C** | OK | negbinomial.size | 3.31E-11 | 3.69E-09 | UBE2C | TRUE |
| **HLA-DPA1** | OK | negbinomial.size | 3.46E-11 | 3.84E-09 | HLA-DPA1 | TRUE |
| **CALM2** | OK | negbinomial.size | 4.21E-11 | 4.65E-09 | CALM2 | TRUE |
| **SERPINA1** | OK | negbinomial.size | 4.52E-11 | 4.95E-09 | SERPINA1 | TRUE |
| **PLAU** | OK | negbinomial.size | 4.65E-11 | 5.08E-09 | PLAU | TRUE |
| **TNFAIP3** | OK | negbinomial.size | 5.02E-11 | 5.46E-09 | TNFAIP3 | TRUE |
| **PTTG1** | OK | negbinomial.size | 5.65E-11 | 6.11E-09 | PTTG1 | TRUE |
| **TSPO** | OK | negbinomial.size | 6.35E-11 | 6.83E-09 | TSPO | TRUE |
| **MARCKS** | OK | negbinomial.size | 7.37E-11 | 7.89E-09 | MARCKS | TRUE |
| **VAMP5** | OK | negbinomial.size | 8.62E-11 | 9.19E-09 | VAMP5 | TRUE |
| **CXCL14** | OK | negbinomial.size | 9.39E-11 | 9.96E-09 | CXCL14 | FALSE |
| **RPL37** | OK | negbinomial.size | 9.55E-11 | 1.01E-08 | RPL37 | TRUE |
| **S100A1** | OK | negbinomial.size | 1.09E-10 | 1.15E-08 | S100A1 | TRUE |
| **SEC61G** | OK | negbinomial.size | 1.1E-10 | 1.15E-08 | SEC61G | TRUE |
| **STK17B** | OK | negbinomial.size | 1.21E-10 | 1.26E-08 | STK17B | TRUE |
| **PLD3** | OK | negbinomial.size | 1.3E-10 | 1.35E-08 | PLD3 | TRUE |
| **RNASE6** | OK | negbinomial.size | 1.32E-10 | 1.36E-08 | RNASE6 | TRUE |
| **KCNQ1OT1** | OK | negbinomial.size | 1.53E-10 | 1.57E-08 | KCNQ1OT1 | TRUE |
| **IFI44L** | OK | negbinomial.size | 2.09E-10 | 2.14E-08 | IFI44L | TRUE |
| **CXCL3** | OK | negbinomial.size | 2.2E-10 | 2.24E-08 | CXCL3 | TRUE |
| **NCF1** | OK | negbinomial.size | 2.22E-10 | 2.25E-08 | NCF1 | TRUE |
| **COX6C** | OK | negbinomial.size | 2.57E-10 | 2.59E-08 | COX6C | TRUE |
| **MRPL18** | OK | negbinomial.size | 2.6E-10 | 2.61E-08 | MRPL18 | TRUE |
| **ELL2** | OK | negbinomial.size | 2.61E-10 | 2.61E-08 | ELL2 | TRUE |
| **RPS9** | OK | negbinomial.size | 2.78E-10 | 2.76E-08 | RPS9 | TRUE |
| **HSD11B1** | OK | negbinomial.size | 2.79E-10 | 2.76E-08 | HSD11B1 | FALSE |
| **DDX5** | OK | negbinomial.size | 2.86E-10 | 2.82E-08 | DDX5 | TRUE |
| **ADAM28** | OK | negbinomial.size | 3.54E-10 | 3.48E-08 | ADAM28 | TRUE |
| **CCL19** | OK | negbinomial.size | 3.56E-10 | 3.48E-08 | CCL19 | FALSE |
| **NUPR1** | OK | negbinomial.size | 3.71E-10 | 3.61E-08 | NUPR1 | TRUE |
| **ID2** | OK | negbinomial.size | 4.27E-10 | 4.14E-08 | ID2 | TRUE |
| **ACTB** | OK | negbinomial.size | 4.92E-10 | 4.75E-08 | ACTB | TRUE |
| **CD63** | OK | negbinomial.size | 5.31E-10 | 5.1E-08 | CD63 | TRUE |
| **RGS10** | OK | negbinomial.size | 8.25E-10 | 7.89E-08 | RGS10 | TRUE |
| **TIMP1** | OK | negbinomial.size | 9.04E-10 | 8.61E-08 | TIMP1 | TRUE |
| **LINC00936** | OK | negbinomial.size | 1.01E-09 | 9.55E-08 | LINC00936 | TRUE |
| **ATP5E** | OK | negbinomial.size | 1.15E-09 | 1.09E-07 | ATP5E | TRUE |
| **DNAJA1** | OK | negbinomial.size | 1.16E-09 | 1.09E-07 | DNAJA1 | TRUE |
| **AREG** | OK | negbinomial.size | 1.27E-09 | 1.19E-07 | AREG | TRUE |
| **RPSA** | OK | negbinomial.size | 1.43E-09 | 1.33E-07 | RPSA | TRUE |
| **REL** | OK | negbinomial.size | 1.46E-09 | 1.35E-07 | REL | TRUE |
| **ENO1** | OK | negbinomial.size | 1.66E-09 | 1.53E-07 | ENO1 | TRUE |
| **ATF4** | OK | negbinomial.size | 1.76E-09 | 1.63E-07 | ATF4 | TRUE |
| **SLAMF7** | OK | negbinomial.size | 1.78E-09 | 1.63E-07 | SLAMF7 | TRUE |
| **SRSF5** | OK | negbinomial.size | 1.83E-09 | 1.68E-07 | SRSF5 | TRUE |
| **COL1A1** | OK | negbinomial.size | 1.99E-09 | 1.81E-07 | COL1A1 | TRUE |
| **CLEC4E** | OK | negbinomial.size | 2.01E-09 | 1.82E-07 | CLEC4E | TRUE |
| **CCNH** | OK | negbinomial.size | 2.2E-09 | 1.98E-07 | CCNH | TRUE |
| **BHLHE41** | OK | negbinomial.size | 2.21E-09 | 1.99E-07 | BHLHE41 | TRUE |
| **CCL20** | OK | negbinomial.size | 2.32E-09 | 2.07E-07 | CCL20 | TRUE |
| **C1QA** | OK | negbinomial.size | 2.37E-09 | 2.1E-07 | C1QA | TRUE |
| **RPL29** | OK | negbinomial.size | 2.37E-09 | 2.1E-07 | RPL29 | TRUE |
| **MIF** | OK | negbinomial.size | 2.64E-09 | 2.34E-07 | MIF | TRUE |
| **EREG** | OK | negbinomial.size | 2.82E-09 | 2.49E-07 | EREG | TRUE |
| **KLF4** | OK | negbinomial.size | 3.09E-09 | 2.71E-07 | KLF4 | TRUE |
| **RARRES3** | OK | negbinomial.size | 3.21E-09 | 2.81E-07 | RARRES3 | TRUE |
| **ALDOA** | OK | negbinomial.size | 3.5E-09 | 3.05E-07 | ALDOA | TRUE |
| **IER5** | OK | negbinomial.size | 3.57E-09 | 3.09E-07 | IER5 | TRUE |
| **ATP5J2** | OK | negbinomial.size | 3.75E-09 | 3.24E-07 | ATP5J2 | TRUE |
| **SERF2** | OK | negbinomial.size | 3.85E-09 | 3.32E-07 | SERF2 | TRUE |
| **CYP27A1** | OK | negbinomial.size | 4.35E-09 | 3.73E-07 | CYP27A1 | TRUE |
| **SGK1** | OK | negbinomial.size | 4.48E-09 | 3.83E-07 | SGK1 | TRUE |
| **EEF1A1** | OK | negbinomial.size | 4.61E-09 | 3.92E-07 | EEF1A1 | TRUE |
| **SPARCL1** | OK | negbinomial.size | 5.56E-09 | 4.71E-07 | SPARCL1 | TRUE |
| **PRDM1** | OK | negbinomial.size | 5.73E-09 | 4.84E-07 | PRDM1 | TRUE |
| **LSP1** | OK | negbinomial.size | 5.88E-09 | 4.95E-07 | LSP1 | TRUE |
| **GSTO1** | OK | negbinomial.size | 5.98E-09 | 5.01E-07 | GSTO1 | TRUE |
| **IRF1** | OK | negbinomial.size | 6.71E-09 | 5.6E-07 | IRF1 | TRUE |
| **RPL21** | OK | negbinomial.size | 7.36E-09 | 6.13E-07 | RPL21 | TRUE |
| **NR4A3** | OK | negbinomial.size | 7.42E-09 | 6.15E-07 | NR4A3 | TRUE |
| **STAT1** | OK | negbinomial.size | 7.7E-09 | 6.36E-07 | STAT1 | TRUE |
| **HCST** | OK | negbinomial.size | 7.76E-09 | 6.38E-07 | HCST | TRUE |
| **RPL31** | OK | negbinomial.size | 7.83E-09 | 6.42E-07 | RPL31 | TRUE |
| **EMP1** | OK | negbinomial.size | 7.94E-09 | 6.49E-07 | EMP1 | TRUE |
| **ID3** | OK | negbinomial.size | 8.25E-09 | 6.72E-07 | ID3 | TRUE |
| **ZFP36L2** | OK | negbinomial.size | 8.32E-09 | 6.75E-07 | ZFP36L2 | TRUE |
| **GADD45B** | OK | negbinomial.size | 8.46E-09 | 6.82E-07 | GADD45B | TRUE |
| **ATP5EP2** | OK | negbinomial.size | 8.48E-09 | 6.82E-07 | ATP5EP2 | TRUE |
| **PFN1** | OK | negbinomial.size | 8.66E-09 | 6.95E-07 | PFN1 | TRUE |
| **IFIT1** | OK | negbinomial.size | 8.75E-09 | 7E-07 | IFIT1 | TRUE |
| **RPL18** | OK | negbinomial.size | 1.05E-08 | 8.38E-07 | RPL18 | TRUE |
| **CTSB** | OK | negbinomial.size | 1.1E-08 | 8.73E-07 | CTSB | TRUE |
| **TWISTNB** | OK | negbinomial.size | 1.14E-08 | 9.02E-07 | TWISTNB | TRUE |
| **HLA-DRA** | OK | negbinomial.size | 1.32E-08 | 1.04E-06 | HLA-DRA | TRUE |
| **MEG3** | OK | negbinomial.size | 1.34E-08 | 1.05E-06 | MEG3 | FALSE |
| **GABARAP** | OK | negbinomial.size | 1.37E-08 | 1.07E-06 | GABARAP | TRUE |
| **TNS3** | OK | negbinomial.size | 1.41E-08 | 1.1E-06 | TNS3 | TRUE |
| **RPS12** | OK | negbinomial.size | 1.47E-08 | 1.14E-06 | RPS12 | TRUE |
| **BAG3** | OK | negbinomial.size | 1.59E-08 | 1.23E-06 | BAG3 | TRUE |
| **MAFB** | OK | negbinomial.size | 1.59E-08 | 1.23E-06 | MAFB | TRUE |
| **IGHV1-18** | OK | negbinomial.size | 1.71E-08 | 1.31E-06 | IGHV1-18 | FALSE |
| **KLRB1** | OK | negbinomial.size | 1.85E-08 | 1.42E-06 | KLRB1 | TRUE |
| **DYNLL1** | OK | negbinomial.size | 1.92E-08 | 1.47E-06 | DYNLL1 | TRUE |
| **FOSB** | OK | negbinomial.size | 2.02E-08 | 1.54E-06 | FOSB | TRUE |
| **RPS23** | OK | negbinomial.size | 2.04E-08 | 1.55E-06 | RPS23 | TRUE |
| **TMSB10** | OK | negbinomial.size | 2.08E-08 | 1.58E-06 | TMSB10 | TRUE |
| **P2RY13** | OK | negbinomial.size | 2.3E-08 | 1.73E-06 | P2RY13 | TRUE |
| **BTG1** | OK | negbinomial.size | 2.46E-08 | 1.84E-06 | BTG1 | TRUE |
| **GADD45G** | OK | negbinomial.size | 2.46E-08 | 1.84E-06 | GADD45G | TRUE |
| **DDIT3** | OK | negbinomial.size | 2.49E-08 | 1.86E-06 | DDIT3 | TRUE |
| **SLAMF8** | OK | negbinomial.size | 2.49E-08 | 1.86E-06 | SLAMF8 | TRUE |
| **LPAR6** | OK | negbinomial.size | 2.51E-08 | 1.86E-06 | LPAR6 | TRUE |
| **JUNB** | OK | negbinomial.size | 2.52E-08 | 1.86E-06 | JUNB | TRUE |
| **AKAP13** | OK | negbinomial.size | 2.73E-08 | 2.01E-06 | AKAP13 | TRUE |
| **SYAP1** | OK | negbinomial.size | 2.89E-08 | 2.12E-06 | SYAP1 | TRUE |
| **RCAN1** | OK | negbinomial.size | 2.99E-08 | 2.18E-06 | RCAN1 | TRUE |
| **DCD** | OK | negbinomial.size | 3.13E-08 | 2.28E-06 | DCD | TRUE |
| **TSC22D1** | OK | negbinomial.size | 3.2E-08 | 2.32E-06 | TSC22D1 | TRUE |
| **SRGN** | OK | negbinomial.size | 3.28E-08 | 2.37E-06 | SRGN | TRUE |
| **CD38** | OK | negbinomial.size | 3.4E-08 | 2.45E-06 | CD38 | TRUE |
| **SLC11A1** | OK | negbinomial.size | 3.61E-08 | 2.6E-06 | SLC11A1 | TRUE |
| **METRNL** | OK | negbinomial.size | 3.62E-08 | 2.6E-06 | METRNL | TRUE |
| **HBA2** | OK | negbinomial.size | 3.64E-08 | 2.6E-06 | HBA2 | TRUE |
| **MALAT1** | OK | negbinomial.size | 4.22E-08 | 3.01E-06 | MALAT1 | TRUE |
| **RASSF4** | OK | negbinomial.size | 4.49E-08 | 3.19E-06 | RASSF4 | TRUE |
| **RAB13** | OK | negbinomial.size | 4.7E-08 | 3.33E-06 | RAB13 | TRUE |
| **ABL2** | OK | negbinomial.size | 4.73E-08 | 3.34E-06 | ABL2 | TRUE |
| **RMDN3** | OK | negbinomial.size | 4.76E-08 | 3.35E-06 | RMDN3 | TRUE |
| **WARS** | OK | negbinomial.size | 4.91E-08 | 3.45E-06 | WARS | TRUE |
| **ANKRD22** | OK | negbinomial.size | 5.05E-08 | 3.53E-06 | ANKRD22 | TRUE |
| **DENND4C** | OK | negbinomial.size | 5.29E-08 | 3.69E-06 | DENND4C | TRUE |
| **COL3A1** | OK | negbinomial.size | 5.83E-08 | 4.05E-06 | COL3A1 | TRUE |
| **ZNF331** | OK | negbinomial.size | 5.84E-08 | 4.05E-06 | ZNF331 | TRUE |
| **TNFSF10** | OK | negbinomial.size | 5.94E-08 | 4.11E-06 | TNFSF10 | TRUE |
| **EIF4A2** | OK | negbinomial.size | 6.26E-08 | 4.31E-06 | EIF4A2 | TRUE |
| **CHMP1B** | OK | negbinomial.size | 6.34E-08 | 4.35E-06 | CHMP1B | TRUE |
| **GM2A** | OK | negbinomial.size | 7.37E-08 | 5.05E-06 | GM2A | TRUE |
| **MT-ND4L** | OK | negbinomial.size | 7.69E-08 | 5.25E-06 | MT-ND4L | TRUE |
| **EZR** | OK | negbinomial.size | 8.03E-08 | 5.46E-06 | EZR | TRUE |
| **HSPA8** | OK | negbinomial.size | 8.06E-08 | 5.46E-06 | HSPA8 | TRUE |
| **FCN1** | OK | negbinomial.size | 8.16E-08 | 5.52E-06 | FCN1 | TRUE |
| **TIMM8B** | OK | negbinomial.size | 8.4E-08 | 5.66E-06 | TIMM8B | TRUE |
| **RPS19** | OK | negbinomial.size | 8.9E-08 | 5.98E-06 | RPS19 | TRUE |
| **TMBIM6** | OK | negbinomial.size | 9.4E-08 | 6.3E-06 | TMBIM6 | TRUE |
| **RPS3A** | OK | negbinomial.size | 9.55E-08 | 6.38E-06 | RPS3A | TRUE |
| **SIGLEC10** | OK | negbinomial.size | 1E-07 | 6.66E-06 | SIGLEC10 | TRUE |
| **FCGR1A** | OK | negbinomial.size | 1E-07 | 6.66E-06 | FCGR1A | TRUE |
| **RPLP0** | OK | negbinomial.size | 1.01E-07 | 6.69E-06 | RPLP0 | TRUE |
| **RPS16** | OK | negbinomial.size | 1.03E-07 | 6.77E-06 | RPS16 | TRUE |
| **HPCAL1** | OK | negbinomial.size | 1.03E-07 | 6.79E-06 | HPCAL1 | TRUE |
| **LILRB4** | OK | negbinomial.size | 1.05E-07 | 6.88E-06 | LILRB4 | TRUE |
| **TFRC** | OK | negbinomial.size | 1.05E-07 | 6.88E-06 | TFRC | TRUE |
| **NR4A1** | OK | negbinomial.size | 1.07E-07 | 6.97E-06 | NR4A1 | TRUE |
| **SLC2A3** | OK | negbinomial.size | 1.16E-07 | 7.58E-06 | SLC2A3 | TRUE |
| **TMEM176A** | OK | negbinomial.size | 1.24E-07 | 8.02E-06 | TMEM176A | TRUE |
| **RPL7A** | OK | negbinomial.size | 1.25E-07 | 8.07E-06 | RPL7A | TRUE |
| **ZFAND5** | OK | negbinomial.size | 1.25E-07 | 8.07E-06 | ZFAND5 | TRUE |
| **LHFPL2** | OK | negbinomial.size | 1.28E-07 | 8.21E-06 | LHFPL2 | TRUE |
| **SELK** | OK | negbinomial.size | 1.36E-07 | 8.7E-06 | SELK | TRUE |
| **NOP10** | OK | negbinomial.size | 1.45E-07 | 9.26E-06 | NOP10 | TRUE |
| **RGS16** | OK | negbinomial.size | 1.52E-07 | 9.68E-06 | RGS16 | TRUE |
| **ACP2** | OK | negbinomial.size | 1.62E-07 | 1.03E-05 | ACP2 | TRUE |
| **UCP2** | OK | negbinomial.size | 1.64E-07 | 1.04E-05 | UCP2 | TRUE |
| **HBEGF** | OK | negbinomial.size | 1.65E-07 | 1.04E-05 | HBEGF | TRUE |
| **JMJD1C** | OK | negbinomial.size | 1.7E-07 | 1.07E-05 | JMJD1C | TRUE |
| **EIF5** | OK | negbinomial.size | 1.72E-07 | 1.08E-05 | EIF5 | TRUE |
| **SLA** | OK | negbinomial.size | 1.81E-07 | 1.14E-05 | SLA | TRUE |
| **PLD4** | OK | negbinomial.size | 1.97E-07 | 1.23E-05 | PLD4 | TRUE |
| **CALD1** | OK | negbinomial.size | 2.04E-07 | 1.27E-05 | CALD1 | TRUE |
| **JUN** | OK | negbinomial.size | 2.16E-07 | 1.34E-05 | JUN | TRUE |
| **MTRNR2L8** | OK | negbinomial.size | 2.16E-07 | 1.34E-05 | MTRNR2L8 | TRUE |
| **LTA4H** | OK | negbinomial.size | 2.29E-07 | 1.42E-05 | LTA4H | TRUE |
| **RTN4** | OK | negbinomial.size | 2.35E-07 | 1.45E-05 | RTN4 | TRUE |
| **KRT15** | OK | negbinomial.size | 2.49E-07 | 1.53E-05 | KRT15 | TRUE |
| **GAPDH** | OK | negbinomial.size | 2.5E-07 | 1.53E-05 | GAPDH | TRUE |
| **CXCL1** | OK | negbinomial.size | 2.72E-07 | 1.66E-05 | CXCL1 | TRUE |
| **NRP1** | OK | negbinomial.size | 2.78E-07 | 1.7E-05 | NRP1 | TRUE |
| **NCEH1** | OK | negbinomial.size | 2.81E-07 | 1.71E-05 | NCEH1 | TRUE |
| **CCL13** | OK | negbinomial.size | 2.81E-07 | 1.71E-05 | CCL13 | TRUE |
| **TPM4** | OK | negbinomial.size | 2.83E-07 | 1.71E-05 | TPM4 | TRUE |
| **CTSS** | OK | negbinomial.size | 2.86E-07 | 1.72E-05 | CTSS | TRUE |
| **RPS6** | OK | negbinomial.size | 2.97E-07 | 1.78E-05 | RPS6 | TRUE |
| **VEGFA** | OK | negbinomial.size | 3.06E-07 | 1.84E-05 | VEGFA | TRUE |
| **DBI** | OK | negbinomial.size | 3.12E-07 | 1.87E-05 | DBI | TRUE |
| **MTRNR2L12** | OK | negbinomial.size | 3.14E-07 | 1.87E-05 | MTRNR2L12 | TRUE |
| **MITF** | OK | negbinomial.size | 3.17E-07 | 1.89E-05 | MITF | TRUE |
| **RPL3** | OK | negbinomial.size | 3.26E-07 | 1.93E-05 | RPL3 | TRUE |
| **RPS4X** | OK | negbinomial.size | 3.26E-07 | 1.93E-05 | RPS4X | TRUE |
| **SCGB1D2** | OK | negbinomial.size | 3.33E-07 | 1.96E-05 | SCGB1D2 | FALSE |
| **ROMO1** | OK | negbinomial.size | 3.51E-07 | 2.07E-05 | ROMO1 | TRUE |
| **RP11-386I14.4** | OK | negbinomial.size | 3.53E-07 | 2.07E-05 | RP11-386I14.4 | TRUE |
| **SCARB2** | OK | negbinomial.size | 3.65E-07 | 2.13E-05 | SCARB2 | TRUE |
| **NAGK** | OK | negbinomial.size | 3.65E-07 | 2.13E-05 | NAGK | TRUE |
| **IFI30** | OK | negbinomial.size | 3.97E-07 | 2.31E-05 | IFI30 | TRUE |
| **RPLP1** | OK | negbinomial.size | 4.12E-07 | 2.4E-05 | RPLP1 | TRUE |
| **PLTP** | OK | negbinomial.size | 4.68E-07 | 2.72E-05 | PLTP | TRUE |
| **TIMP2** | OK | negbinomial.size | 4.77E-07 | 2.76E-05 | TIMP2 | TRUE |
| **CRIP1** | OK | negbinomial.size | 4.9E-07 | 2.82E-05 | CRIP1 | TRUE |
| **AP2S1** | OK | negbinomial.size | 5.07E-07 | 2.92E-05 | AP2S1 | TRUE |
| **ALCAM** | OK | negbinomial.size | 5.11E-07 | 2.93E-05 | ALCAM | TRUE |
| **SERPING1** | OK | negbinomial.size | 5.24E-07 | 3E-05 | SERPING1 | TRUE |
| **RPS8** | OK | negbinomial.size | 5.35E-07 | 3.05E-05 | RPS8 | TRUE |
| **SARAF** | OK | negbinomial.size | 5.54E-07 | 3.16E-05 | SARAF | TRUE |
| **PLXDC1** | OK | negbinomial.size | 5.66E-07 | 3.22E-05 | PLXDC1 | TRUE |
| **RPS15A** | OK | negbinomial.size | 5.68E-07 | 3.22E-05 | RPS15A | TRUE |
| **HAMP** | OK | negbinomial.size | 5.83E-07 | 3.29E-05 | HAMP | TRUE |
| **APOBEC3A** | OK | negbinomial.size | 5.91E-07 | 3.33E-05 | APOBEC3A | TRUE |
| **MYL6** | OK | negbinomial.size | 6.43E-07 | 3.61E-05 | MYL6 | TRUE |
| **FOLR2** | OK | negbinomial.size | 6.56E-07 | 3.68E-05 | FOLR2 | TRUE |
| **KRT81** | OK | negbinomial.size | 7.71E-07 | 4.32E-05 | KRT81 | TRUE |
| **IDO1** | OK | negbinomial.size | 8.22E-07 | 4.59E-05 | IDO1 | FALSE |
| **RP11-81H14.2** | OK | negbinomial.size | 8.98E-07 | 5E-05 | RP11-81H14.2 | FALSE |
| **NEXN** | OK | negbinomial.size | 9.17E-07 | 5.09E-05 | NEXN | FALSE |
| **MT2A** | OK | negbinomial.size | 9.27E-07 | 5.14E-05 | MT2A | TRUE |
| **RANBP2** | OK | negbinomial.size | 9.35E-07 | 5.17E-05 | RANBP2 | TRUE |
| **F13A1** | OK | negbinomial.size | 9.66E-07 | 5.32E-05 | F13A1 | TRUE |
| **RP11-61J19.5** | OK | negbinomial.size | 9.84E-07 | 5.41E-05 | RP11-61J19.5 | TRUE |
| **C5AR1** | OK | negbinomial.size | 1.01E-06 | 5.55E-05 | C5AR1 | TRUE |
| **FCER1G** | OK | negbinomial.size | 1.02E-06 | 5.57E-05 | FCER1G | TRUE |
| **TMSB4X** | OK | negbinomial.size | 1.04E-06 | 5.68E-05 | TMSB4X | TRUE |
| **CORO1C** | OK | negbinomial.size | 1.07E-06 | 5.82E-05 | CORO1C | TRUE |
| **RPL34** | OK | negbinomial.size | 1.08E-06 | 5.84E-05 | RPL34 | TRUE |
| **IFNGR1** | OK | negbinomial.size | 1.1E-06 | 5.95E-05 | IFNGR1 | TRUE |
| **SFRP1** | OK | negbinomial.size | 1.11E-06 | 5.99E-05 | SFRP1 | FALSE |
| **H2AFY** | OK | negbinomial.size | 1.15E-06 | 6.22E-05 | H2AFY | TRUE |
| **RPL35A** | OK | negbinomial.size | 1.18E-06 | 6.37E-05 | RPL35A | TRUE |
| **RPL9** | OK | negbinomial.size | 1.19E-06 | 6.39E-05 | RPL9 | TRUE |
| **RPL13** | OK | negbinomial.size | 1.23E-06 | 6.6E-05 | RPL13 | TRUE |
| **SMAP2** | OK | negbinomial.size | 1.35E-06 | 7.17E-05 | SMAP2 | TRUE |
| **DYNLT1** | OK | negbinomial.size | 1.35E-06 | 7.17E-05 | DYNLT1 | TRUE |
| **RPL11** | OK | negbinomial.size | 1.37E-06 | 7.3E-05 | RPL11 | TRUE |
| **SDC3** | OK | negbinomial.size | 1.43E-06 | 7.59E-05 | SDC3 | TRUE |
| **ARL4C** | OK | negbinomial.size | 1.52E-06 | 8.05E-05 | ARL4C | TRUE |
| **ALAS1** | OK | negbinomial.size | 1.53E-06 | 8.07E-05 | ALAS1 | TRUE |
| **CCT6A** | OK | negbinomial.size | 1.56E-06 | 8.19E-05 | CCT6A | TRUE |
| **TXNIP** | OK | negbinomial.size | 1.71E-06 | 8.98E-05 | TXNIP | TRUE |
| **CD74** | OK | negbinomial.size | 1.77E-06 | 9.29E-05 | CD74 | TRUE |
| **APOC2** | OK | negbinomial.size | 1.8E-06 | 9.4E-05 | APOC2 | TRUE |
| **OLFML3** | OK | negbinomial.size | 1.86E-06 | 9.68E-05 | OLFML3 | TRUE |
| **DUSP1** | OK | negbinomial.size | 1.86E-06 | 9.69E-05 | DUSP1 | TRUE |
| **COTL1** | OK | negbinomial.size | 1.87E-06 | 9.7E-05 | COTL1 | TRUE |
| **HLA-DQA1** | OK | negbinomial.size | 1.88E-06 | 9.76E-05 | HLA-DQA1 | TRUE |
| **CKS2** | OK | negbinomial.size | 2.05E-06 | 0.000105654 | CKS2 | TRUE |
| **RP11-160E2.6** | OK | negbinomial.size | 2.05E-06 | 0.000105654 | RP11-160E2.6 | TRUE |
| **NEAT1** | OK | negbinomial.size | 2.13E-06 | 0.000109375 | NEAT1 | TRUE |
| **PKM** | OK | negbinomial.size | 2.15E-06 | 0.000110539 | PKM | TRUE |
| **SEMA3C** | OK | negbinomial.size | 2.17E-06 | 0.000111247 | SEMA3C | FALSE |
| **FCGR3A** | OK | negbinomial.size | 2.23E-06 | 0.000114003 | FCGR3A | TRUE |
| **CYR61** | OK | negbinomial.size | 2.29E-06 | 0.000116733 | CYR61 | FALSE |
| **SIGLEC8** | OK | negbinomial.size | 2.33E-06 | 0.000118239 | SIGLEC8 | TRUE |
| **SRGAP1** | OK | negbinomial.size | 2.33E-06 | 0.000118239 | SRGAP1 | TRUE |
| **PHPT1** | OK | negbinomial.size | 2.39E-06 | 0.0001208 | PHPT1 | TRUE |
| **CLTC** | OK | negbinomial.size | 2.39E-06 | 0.0001208 | CLTC | TRUE |
| **TALDO1** | OK | negbinomial.size | 2.44E-06 | 0.000122991 | TALDO1 | TRUE |
| **SLC7A5** | OK | negbinomial.size | 2.47E-06 | 0.000124442 | SLC7A5 | TRUE |
| **CSF1R** | OK | negbinomial.size | 2.5E-06 | 0.000125387 | CSF1R | TRUE |
| **PNRC1** | OK | negbinomial.size | 2.58E-06 | 0.000129155 | PNRC1 | TRUE |
| **H3F3A** | OK | negbinomial.size | 2.76E-06 | 0.00013799 | H3F3A | TRUE |
| **TCEB2** | OK | negbinomial.size | 2.8E-06 | 0.000139512 | TCEB2 | TRUE |
| **SLC25A24** | OK | negbinomial.size | 2.82E-06 | 0.000140095 | SLC25A24 | TRUE |
| **RP11-22N19.2** | OK | negbinomial.size | 2.84E-06 | 0.000140822 | RP11-22N19.2 | TRUE |
| **HLA-DMB** | OK | negbinomial.size | 2.85E-06 | 0.000141355 | HLA-DMB | TRUE |
| **PABPC4** | OK | negbinomial.size | 2.91E-06 | 0.000143926 | PABPC4 | TRUE |
| **MMP14** | OK | negbinomial.size | 2.97E-06 | 0.000146375 | MMP14 | TRUE |
| **ISG20** | OK | negbinomial.size | 2.99E-06 | 0.000147281 | ISG20 | TRUE |
| **MIDN** | OK | negbinomial.size | 3.01E-06 | 0.000147765 | MIDN | TRUE |
| **MFSD12** | OK | negbinomial.size | 3.07E-06 | 0.00015057 | MFSD12 | TRUE |
| **USP36** | OK | negbinomial.size | 3.13E-06 | 0.000152845 | USP36 | TRUE |
| **YPEL5** | OK | negbinomial.size | 3.25E-06 | 0.000158398 | YPEL5 | TRUE |
| **TAGLN2** | OK | negbinomial.size | 3.41E-06 | 0.000165694 | TAGLN2 | TRUE |
| **RASGEF1B** | OK | negbinomial.size | 3.5E-06 | 0.00017009 | RASGEF1B | TRUE |
| **CLK1** | OK | negbinomial.size | 3.55E-06 | 0.000172137 | CLK1 | TRUE |
| **AQP9** | OK | negbinomial.size | 3.58E-06 | 0.00017312 | AQP9 | TRUE |
| **HSPA1B** | OK | negbinomial.size | 3.6E-06 | 0.000173818 | HSPA1B | TRUE |
| **PSMA3** | OK | negbinomial.size | 3.71E-06 | 0.000178387 | PSMA3 | TRUE |
| **MYLIP** | OK | negbinomial.size | 3.74E-06 | 0.000179604 | MYLIP | TRUE |
| **TANK** | OK | negbinomial.size | 3.85E-06 | 0.000184737 | TANK | TRUE |
| **MGST3** | OK | negbinomial.size | 3.89E-06 | 0.000186079 | MGST3 | TRUE |
| **BCL2A1** | OK | negbinomial.size | 4.04E-06 | 0.000192689 | BCL2A1 | TRUE |
| **TAGLN** | OK | negbinomial.size | 4.1E-06 | 0.000195052 | TAGLN | TRUE |
| **RPN1** | OK | negbinomial.size | 4.17E-06 | 0.000198012 | RPN1 | TRUE |
| **PTGS2** | OK | negbinomial.size | 4.26E-06 | 0.000201928 | PTGS2 | TRUE |
| **DRAM1** | OK | negbinomial.size | 4.56E-06 | 0.000215691 | DRAM1 | TRUE |
| **PTGER4** | OK | negbinomial.size | 4.61E-06 | 0.000217454 | PTGER4 | TRUE |
| **KDM6B** | OK | negbinomial.size | 4.61E-06 | 0.000217454 | KDM6B | TRUE |
| **RNF122** | OK | negbinomial.size | 4.65E-06 | 0.000218439 | RNF122 | TRUE |
| **ALDH1A1** | OK | negbinomial.size | 4.71E-06 | 0.000221241 | ALDH1A1 | TRUE |
| **WTAP** | OK | negbinomial.size | 5.08E-06 | 0.000237845 | WTAP | TRUE |
| **IL7R** | OK | negbinomial.size | 5.1E-06 | 0.00023835 | IL7R | TRUE |
| **OPTN** | OK | negbinomial.size | 5.11E-06 | 0.0002384 | OPTN | TRUE |
| **MTSS1** | OK | negbinomial.size | 5.28E-06 | 0.000245585 | MTSS1 | TRUE |
| **RPS17** | OK | negbinomial.size | 5.4E-06 | 0.00025072 | RPS17 | TRUE |
| **IRAK1** | OK | negbinomial.size | 5.48E-06 | 0.000253996 | IRAK1 | TRUE |
| **SLC43A3** | OK | negbinomial.size | 5.7E-06 | 0.000263502 | SLC43A3 | TRUE |
| **IGHA1** | OK | negbinomial.size | 5.97E-06 | 0.000275426 | IGHA1 | TRUE |
| **CD44** | OK | negbinomial.size | 6.07E-06 | 0.000279668 | CD44 | TRUE |
| **RPL22L1** | OK | negbinomial.size | 6.15E-06 | 0.000282679 | RPL22L1 | TRUE |
| **HMGB2** | OK | negbinomial.size | 6.36E-06 | 0.000291945 | HMGB2 | TRUE |
| **OTOA** | OK | negbinomial.size | 6.67E-06 | 0.000304941 | OTOA | TRUE |
| **RNU12** | OK | negbinomial.size | 6.68E-06 | 0.000304941 | RNU12 | TRUE |
| **PDGFB** | OK | negbinomial.size | 6.71E-06 | 0.000305718 | PDGFB | TRUE |
| **PLEKHO2** | OK | negbinomial.size | 6.85E-06 | 0.000311788 | PLEKHO2 | TRUE |
| **SRSF7** | OK | negbinomial.size | 6.91E-06 | 0.000313319 | SRSF7 | TRUE |
| **HNRNPA1** | OK | negbinomial.size | 6.91E-06 | 0.000313319 | HNRNPA1 | TRUE |
| **CHD2** | OK | negbinomial.size | 7.01E-06 | 0.000316855 | CHD2 | TRUE |
| **SLC38A2** | OK | negbinomial.size | 7.06E-06 | 0.00031854 | SLC38A2 | TRUE |
| **CFLAR** | OK | negbinomial.size | 7.1E-06 | 0.000319806 | CFLAR | TRUE |
| **PNPLA8** | OK | negbinomial.size | 7.37E-06 | 0.000330714 | PNPLA8 | TRUE |
| **LRPAP1** | OK | negbinomial.size | 7.38E-06 | 0.000330714 | LRPAP1 | TRUE |
| **IGFBP5** | OK | negbinomial.size | 7.39E-06 | 0.000330714 | IGFBP5 | FALSE |
| **APLP2** | OK | negbinomial.size | 7.5E-06 | 0.000335211 | APLP2 | TRUE |
| **CCNL1** | OK | negbinomial.size | 7.6E-06 | 0.000338643 | CCNL1 | TRUE |
| **RP11-670E13.6** | OK | negbinomial.size | 7.61E-06 | 0.000338643 | RP11-670E13.6 | TRUE |
| **C1QC** | OK | negbinomial.size | 7.62E-06 | 0.000338643 | C1QC | TRUE |
| **NPL** | OK | negbinomial.size | 7.77E-06 | 0.000344381 | NPL | TRUE |
| **ST14** | OK | negbinomial.size | 7.98E-06 | 0.000353045 | ST14 | TRUE |
| **ZBTB43** | OK | negbinomial.size | 8.29E-06 | 0.000365832 | ZBTB43 | TRUE |
| **PLEK** | OK | negbinomial.size | 8.48E-06 | 0.000373567 | PLEK | TRUE |
| **YBX1** | OK | negbinomial.size | 8.68E-06 | 0.000381269 | YBX1 | TRUE |
| **SERPINB9** | OK | negbinomial.size | 8.69E-06 | 0.000381269 | SERPINB9 | TRUE |
| **CDA** | OK | negbinomial.size | 8.8E-06 | 0.000385427 | CDA | FALSE |
| **ATP5G3** | OK | negbinomial.size | 9.02E-06 | 0.000394215 | ATP5G3 | TRUE |
| **FLNA** | OK | negbinomial.size | 9.36E-06 | 0.000408192 | FLNA | TRUE |
| **LIMS1** | OK | negbinomial.size | 9.49E-06 | 0.000413138 | LIMS1 | TRUE |
| **AKR1B1** | OK | negbinomial.size | 9.85E-06 | 0.000428123 | AKR1B1 | TRUE |
| **LCN2** | OK | negbinomial.size | 1.03E-05 | 0.000446389 | LCN2 | FALSE |
| **AKR1A1** | OK | negbinomial.size | 1.04E-05 | 0.000448567 | AKR1A1 | TRUE |
| **FAM177A1** | OK | negbinomial.size | 1.05E-05 | 0.000454794 | FAM177A1 | TRUE |
| **SOD2** | OK | negbinomial.size | 1.07E-05 | 0.000463216 | SOD2 | TRUE |
| **CD68** | OK | negbinomial.size | 1.08E-05 | 0.000465272 | CD68 | TRUE |
| **RPS3** | OK | negbinomial.size | 1.09E-05 | 0.000469923 | RPS3 | TRUE |
| **ACSL1** | OK | negbinomial.size | 1.12E-05 | 0.00047886 | ACSL1 | TRUE |
| **GIMAP7** | OK | negbinomial.size | 1.14E-05 | 0.000487066 | GIMAP7 | TRUE |
| **CKS1B** | OK | negbinomial.size | 1.16E-05 | 0.000497479 | CKS1B | TRUE |
| **COL1A2** | OK | negbinomial.size | 1.19E-05 | 0.000506259 | COL1A2 | TRUE |
| **HLA-B** | OK | negbinomial.size | 1.19E-05 | 0.000507592 | HLA-B | TRUE |
| **CSTA** | OK | negbinomial.size | 1.21E-05 | 0.000512397 | CSTA | TRUE |
| **KRT19** | OK | negbinomial.size | 1.23E-05 | 0.000519543 | KRT19 | TRUE |
| **CD81** | OK | negbinomial.size | 1.26E-05 | 0.000534518 | CD81 | TRUE |
| **KRT23** | OK | negbinomial.size | 1.32E-05 | 0.000557416 | KRT23 | FALSE |
| **TSPYL2** | OK | negbinomial.size | 1.34E-05 | 0.000562917 | TSPYL2 | TRUE |
| **GALM** | OK | negbinomial.size | 1.42E-05 | 0.000597029 | GALM | TRUE |
| **OTUD1** | OK | negbinomial.size | 1.45E-05 | 0.000606899 | OTUD1 | TRUE |
| **OLR1** | OK | negbinomial.size | 1.52E-05 | 0.000638423 | OLR1 | TRUE |
| **VSIG4** | OK | negbinomial.size | 1.57E-05 | 0.000653847 | VSIG4 | TRUE |
| **FBLN5** | OK | negbinomial.size | 1.57E-05 | 0.000653847 | FBLN5 | FALSE |
| **RB1** | OK | negbinomial.size | 1.6E-05 | 0.000668388 | RB1 | TRUE |
| **RASGRP3** | OK | negbinomial.size | 1.69E-05 | 0.000703879 | RASGRP3 | TRUE |
| **CCR1** | OK | negbinomial.size | 1.83E-05 | 0.000757922 | CCR1 | TRUE |
| **CRYAB** | OK | negbinomial.size | 1.83E-05 | 0.00075867 | CRYAB | TRUE |
| **MGLL** | OK | negbinomial.size | 1.86E-05 | 0.000770839 | MGLL | TRUE |
| **PCID2** | OK | negbinomial.size | 1.91E-05 | 0.000790442 | PCID2 | TRUE |
| **INPPL1** | OK | negbinomial.size | 1.92E-05 | 0.000790442 | INPPL1 | TRUE |
| **STAB1** | OK | negbinomial.size | 2.09E-05 | 0.000860636 | STAB1 | TRUE |
| **CARD16** | OK | negbinomial.size | 2.1E-05 | 0.000864369 | CARD16 | TRUE |
| **AHNAK** | OK | negbinomial.size | 2.14E-05 | 0.00087529 | AHNAK | TRUE |
| **C1S** | OK | negbinomial.size | 2.14E-05 | 0.000877523 | C1S | TRUE |
| **SMPDL3A** | OK | negbinomial.size | 2.17E-05 | 0.000886283 | SMPDL3A | TRUE |
| **UBB** | OK | negbinomial.size | 2.23E-05 | 0.00090782 | UBB | TRUE |
| **VWF** | OK | negbinomial.size | 2.25E-05 | 0.000917205 | VWF | FALSE |
| **LCP1** | OK | negbinomial.size | 2.28E-05 | 0.000927345 | LCP1 | TRUE |
| **TGIF1** | OK | negbinomial.size | 2.29E-05 | 0.000929357 | TGIF1 | TRUE |
| **VPS37B** | OK | negbinomial.size | 2.35E-05 | 0.000951327 | VPS37B | TRUE |
| **PSPH** | OK | negbinomial.size | 2.43E-05 | 0.000980379 | PSPH | FALSE |
| **CD163** | OK | negbinomial.size | 2.44E-05 | 0.000984445 | CD163 | TRUE |
| **LITAF** | OK | negbinomial.size | 2.45E-05 | 0.000984501 | LITAF | TRUE |
| **MPP1** | OK | negbinomial.size | 2.53E-05 | 0.001015327 | MPP1 | TRUE |
| **RALA** | OK | negbinomial.size | 2.57E-05 | 0.001031507 | RALA | TRUE |
| **SCD** | OK | negbinomial.size | 2.6E-05 | 0.001041321 | SCD | TRUE |
| **MS4A4A** | OK | negbinomial.size | 2.61E-05 | 0.001044869 | MS4A4A | TRUE |
| **CES1** | OK | negbinomial.size | 2.69E-05 | 0.001072734 | CES1 | FALSE |
| **ITGB2** | OK | negbinomial.size | 2.73E-05 | 0.001085412 | ITGB2 | TRUE |
| **RNF144B** | OK | negbinomial.size | 2.88E-05 | 0.001145408 | RNF144B | TRUE |
| **FYB** | OK | negbinomial.size | 3.06E-05 | 0.001214739 | FYB | TRUE |
| **PLAC8** | OK | negbinomial.size | 3.07E-05 | 0.001217093 | PLAC8 | FALSE |
| **ACOT13** | OK | negbinomial.size | 3.11E-05 | 0.001230567 | ACOT13 | TRUE |
| **MS4A6A** | OK | negbinomial.size | 3.2E-05 | 0.001263589 | MS4A6A | TRUE |
| **TTYH3** | OK | negbinomial.size | 3.21E-05 | 0.001264002 | TTYH3 | TRUE |
| **LAMTOR2** | OK | negbinomial.size | 3.36E-05 | 0.001320364 | LAMTOR2 | TRUE |
| **RPPH1** | OK | negbinomial.size | 3.44E-05 | 0.001349389 | RPPH1 | TRUE |
| **RPL19** | OK | negbinomial.size | 3.51E-05 | 0.001375681 | RPL19 | TRUE |
| **PIK3IP1** | OK | negbinomial.size | 3.57E-05 | 0.001398589 | PIK3IP1 | TRUE |
| **TRAPPC4** | OK | negbinomial.size | 3.62E-05 | 0.001412841 | TRAPPC4 | TRUE |
| **RNASET2** | OK | negbinomial.size | 3.63E-05 | 0.001413661 | RNASET2 | TRUE |
| **ACOT7** | OK | negbinomial.size | 3.67E-05 | 0.001427829 | ACOT7 | TRUE |
| **BATF** | OK | negbinomial.size | 3.71E-05 | 0.001443032 | BATF | TRUE |
| **ZFAS1** | OK | negbinomial.size | 3.77E-05 | 0.001460805 | ZFAS1 | TRUE |
| **BLVRA** | OK | negbinomial.size | 3.98E-05 | 0.001540891 | BLVRA | TRUE |
| **PTPRE** | OK | negbinomial.size | 3.99E-05 | 0.001540981 | PTPRE | TRUE |
| **CTD-3252C9.4** | OK | negbinomial.size | 4.03E-05 | 0.001556242 | CTD-3252C9.4 | TRUE |
| **LINC00152** | OK | negbinomial.size | 4.07E-05 | 0.001568909 | LINC00152 | TRUE |
| **RPL17** | OK | negbinomial.size | 4.13E-05 | 0.001589401 | RPL17 | TRUE |
| **CALM1** | OK | negbinomial.size | 4.17E-05 | 0.001601087 | CALM1 | TRUE |
| **SDC2** | OK | negbinomial.size | 4.37E-05 | 0.001674953 | SDC2 | TRUE |
| **HNMT** | OK | negbinomial.size | 4.4E-05 | 0.001683491 | HNMT | TRUE |
| **TM4SF19** | OK | negbinomial.size | 4.47E-05 | 0.001705912 | TM4SF19 | FALSE |
| **ACTN1** | OK | negbinomial.size | 4.49E-05 | 0.001711071 | ACTN1 | TRUE |
| **RSRC2** | OK | negbinomial.size | 4.51E-05 | 0.001715079 | RSRC2 | TRUE |
| **C6orf62** | OK | negbinomial.size | 4.64E-05 | 0.001760864 | C6orf62 | TRUE |
| **CDV3** | OK | negbinomial.size | 4.64E-05 | 0.001760864 | CDV3 | TRUE |
| **ETS2** | OK | negbinomial.size | 4.67E-05 | 0.001769163 | ETS2 | TRUE |
| **DDIT4** | OK | negbinomial.size | 4.7E-05 | 0.001778445 | DDIT4 | TRUE |
| **DUSP4** | OK | negbinomial.size | 4.75E-05 | 0.001792033 | DUSP4 | TRUE |
| **FXYD2** | OK | negbinomial.size | 4.82E-05 | 0.001816281 | FXYD2 | FALSE |
| **DNAJB9** | OK | negbinomial.size | 4.85E-05 | 0.001825405 | DNAJB9 | TRUE |
| **CX3CR1** | OK | negbinomial.size | 5.05E-05 | 0.001895285 | CX3CR1 | FALSE |
| **ANKRD37** | OK | negbinomial.size | 5.06E-05 | 0.001897713 | ANKRD37 | TRUE |
| **GSPT1** | OK | negbinomial.size | 5.14E-05 | 0.001924195 | GSPT1 | TRUE |
| **LST1** | OK | negbinomial.size | 5.18E-05 | 0.001935882 | LST1 | TRUE |
| **BID** | OK | negbinomial.size | 5.2E-05 | 0.001939484 | BID | TRUE |
| **GIMAP4** | OK | negbinomial.size | 5.26E-05 | 0.001957415 | GIMAP4 | TRUE |
| **RPS27** | OK | negbinomial.size | 5.33E-05 | 0.001980448 | RPS27 | TRUE |
| **CLTA** | OK | negbinomial.size | 5.37E-05 | 0.001995252 | CLTA | TRUE |
| **TGM2** | OK | negbinomial.size | 5.58E-05 | 0.002069036 | TGM2 | TRUE |
| **ITGB5** | OK | negbinomial.size | 5.72E-05 | 0.002115946 | ITGB5 | TRUE |
| **HNRNPU-AS1** | OK | negbinomial.size | 5.79E-05 | 0.002138097 | HNRNPU-AS1 | TRUE |
| **RBM39** | OK | negbinomial.size | 6.05E-05 | 0.002230329 | RBM39 | TRUE |
| **HLA-DMA** | OK | negbinomial.size | 6.06E-05 | 0.002232209 | HLA-DMA | TRUE |
| **RPL23A** | OK | negbinomial.size | 6.25E-05 | 0.002296415 | RPL23A | TRUE |
| **ZBTB16** | OK | negbinomial.size | 6.26E-05 | 0.002298572 | ZBTB16 | TRUE |
| **GBP4** | OK | negbinomial.size | 6.38E-05 | 0.002336709 | GBP4 | TRUE |
| **TNFRSF11A** | OK | negbinomial.size | 6.42E-05 | 0.002349519 | TNFRSF11A | FALSE |
| **DDX21** | OK | negbinomial.size | 6.6E-05 | 0.002406902 | DDX21 | TRUE |
| **LHFP** | OK | negbinomial.size | 6.6E-05 | 0.002406902 | LHFP | FALSE |
| **DUSP2** | OK | negbinomial.size | 6.64E-05 | 0.002418435 | DUSP2 | TRUE |
| **MYL12A** | OK | negbinomial.size | 6.71E-05 | 0.002438509 | MYL12A | TRUE |
| **SLC12A5** | OK | negbinomial.size | 6.72E-05 | 0.00243913 | SLC12A5 | FALSE |
| **KIAA0101** | OK | negbinomial.size | 6.91E-05 | 0.002500971 | KIAA0101 | TRUE |
| **MMP12** | OK | negbinomial.size | 6.92E-05 | 0.002500971 | MMP12 | FALSE |
| **CYCS** | OK | negbinomial.size | 7.14E-05 | 0.002577649 | CYCS | TRUE |
| **PLK2** | OK | negbinomial.size | 7.16E-05 | 0.002580523 | PLK2 | TRUE |
| **LACTB** | OK | negbinomial.size | 7.25E-05 | 0.002609155 | LACTB | TRUE |
| **PDXK** | OK | negbinomial.size | 7.36E-05 | 0.002646017 | PDXK | TRUE |
| **CLDN4** | OK | negbinomial.size | 7.44E-05 | 0.00266631 | CLDN4 | TRUE |
| **PCNXL2** | OK | negbinomial.size | 7.44E-05 | 0.00266631 | PCNXL2 | FALSE |
| **ATP13A3** | OK | negbinomial.size | 7.5E-05 | 0.002680618 | ATP13A3 | TRUE |
| **PSMB3** | OK | negbinomial.size | 7.59E-05 | 0.002707944 | PSMB3 | TRUE |
| **IDH1** | OK | negbinomial.size | 7.61E-05 | 0.002712222 | IDH1 | TRUE |
| **MAPK1** | OK | negbinomial.size | 7.74E-05 | 0.002752952 | MAPK1 | TRUE |
| **LINC01480** | OK | negbinomial.size | 7.91E-05 | 0.002810579 | LINC01480 | FALSE |
| **PPP1R7** | OK | negbinomial.size | 7.95E-05 | 0.00282044 | PPP1R7 | TRUE |
| **ATG7** | OK | negbinomial.size | 8.06E-05 | 0.002854547 | ATG7 | TRUE |
| **TMEM107** | OK | negbinomial.size | 8.07E-05 | 0.002854672 | TMEM107 | TRUE |
| **APOL3** | OK | negbinomial.size | 8.21E-05 | 0.002896972 | APOL3 | TRUE |
| **RPL24** | OK | negbinomial.size | 8.26E-05 | 0.002912089 | RPL24 | TRUE |
| **CALM3** | OK | negbinomial.size | 8.45E-05 | 0.002975581 | CALM3 | TRUE |
| **FKBP5** | OK | negbinomial.size | 8.51E-05 | 0.002990775 | FKBP5 | TRUE |
| **B4GALT1** | OK | negbinomial.size | 8.56E-05 | 0.003002511 | B4GALT1 | TRUE |
| **ZBTB10** | OK | negbinomial.size | 8.67E-05 | 0.00303877 | ZBTB10 | TRUE |
| **CERS2** | OK | negbinomial.size | 8.71E-05 | 0.0030449 | CERS2 | FALSE |
| **RPL37A** | OK | negbinomial.size | 8.93E-05 | 0.003117584 | RPL37A | TRUE |
| **KCNMA1** | OK | negbinomial.size | 9E-05 | 0.003138945 | KCNMA1 | TRUE |
| **IFRD1** | OK | negbinomial.size | 9.03E-05 | 0.003143419 | IFRD1 | TRUE |
| **MRPS17** | OK | negbinomial.size | 9.11E-05 | 0.003165516 | MRPS17 | FALSE |
| **DENND4A** | OK | negbinomial.size | 9.2E-05 | 0.003192206 | DENND4A | FALSE |
| **PALD1** | OK | negbinomial.size | 9.21E-05 | 0.003192206 | PALD1 | TRUE |
| **THAP2** | OK | negbinomial.size | 9.32E-05 | 0.003221355 | THAP2 | TRUE |
| **FAM177B** | OK | negbinomial.size | 9.33E-05 | 0.003221355 | FAM177B | FALSE |
| **IFITM2** | OK | negbinomial.size | 9.59E-05 | 0.003306789 | IFITM2 | TRUE |
| **RPS18** | OK | negbinomial.size | 9.79E-05 | 0.003370148 | RPS18 | TRUE |
| **PSMB8** | OK | negbinomial.size | 9.94E-05 | 0.003418699 | PSMB8 | TRUE |
| **C1R** | OK | negbinomial.size | 0.000100154 | 0.003438425 | C1R | FALSE |
| **NDRG2** | OK | negbinomial.size | 0.000100617 | 0.00344903 | NDRG2 | TRUE |
| **CCDC107** | OK | negbinomial.size | 0.000101483 | 0.003473372 | CCDC107 | TRUE |
| **TAP1** | OK | negbinomial.size | 0.000102897 | 0.003515679 | TAP1 | TRUE |
| **IGHM** | OK | negbinomial.size | 0.000103033 | 0.003515679 | IGHM | FALSE |
| **RPL10** | OK | negbinomial.size | 0.000104483 | 0.003559752 | RPL10 | TRUE |
| **CIRBP** | OK | negbinomial.size | 0.000104856 | 0.003567009 | CIRBP | TRUE |
| **LILRB5** | OK | negbinomial.size | 0.000106842 | 0.003628623 | LILRB5 | TRUE |
| **SMCO4** | OK | negbinomial.size | 0.000106991 | 0.003628623 | SMCO4 | TRUE |
| **IFI6** | OK | negbinomial.size | 0.000107285 | 0.003633094 | IFI6 | TRUE |
| **H2AFJ** | OK | negbinomial.size | 0.000108286 | 0.003657871 | H2AFJ | TRUE |
| **SLC16A10** | OK | negbinomial.size | 0.000108344 | 0.003657871 | SLC16A10 | TRUE |
| **RPL23** | OK | negbinomial.size | 0.000109101 | 0.003677891 | RPL23 | TRUE |
| **CHD1** | OK | negbinomial.size | 0.000109301 | 0.003679085 | CHD1 | TRUE |
| **OGFRL1** | OK | negbinomial.size | 0.000110965 | 0.003729493 | OGFRL1 | TRUE |
| **COL15A1** | OK | negbinomial.size | 0.000111558 | 0.003743786 | COL15A1 | FALSE |
| **CARKD** | OK | negbinomial.size | 0.00011217 | 0.003758707 | CARKD | TRUE |
| **RAB20** | OK | negbinomial.size | 0.000114139 | 0.003818972 | RAB20 | TRUE |
| **ADRB2** | OK | negbinomial.size | 0.00011906 | 0.003977659 | ADRB2 | TRUE |
| **GIMAP2** | OK | negbinomial.size | 0.000121041 | 0.004037813 | GIMAP2 | TRUE |
| **CD48** | OK | negbinomial.size | 0.000125693 | 0.004186766 | CD48 | TRUE |
| **RNASEK** | OK | negbinomial.size | 0.000129163 | 0.004295958 | RNASEK | TRUE |
| **ACE** | OK | negbinomial.size | 0.00013275 | 0.004408724 | ACE | TRUE |
| **CHORDC1** | OK | negbinomial.size | 0.000133917 | 0.004440881 | CHORDC1 | TRUE |
| **RPS27A** | OK | negbinomial.size | 0.000140991 | 0.004668564 | RPS27A | TRUE |
| **RPS13** | OK | negbinomial.size | 0.000154786 | 0.005117781 | RPS13 | TRUE |
| **PLP2** | OK | negbinomial.size | 0.000156392 | 0.005163255 | PLP2 | TRUE |
| **JMY** | OK | negbinomial.size | 0.000157805 | 0.005202222 | JMY | TRUE |
| **USMG5** | OK | negbinomial.size | 0.000159443 | 0.005248492 | USMG5 | TRUE |
| **RPL28** | OK | negbinomial.size | 0.000161892 | 0.005318209 | RPL28 | TRUE |
| **CREG1** | OK | negbinomial.size | 0.000162036 | 0.005318209 | CREG1 | TRUE |
| **SLPI** | OK | negbinomial.size | 0.000163191 | 0.00534828 | SLPI | TRUE |
| **CD47** | OK | negbinomial.size | 0.00016497 | 0.005393711 | CD47 | TRUE |
| **AC090498.1** | OK | negbinomial.size | 0.000165059 | 0.005393711 | AC090498.1 | TRUE |
| **EGR2** | OK | negbinomial.size | 0.000169324 | 0.005524999 | EGR2 | TRUE |
| **RPL4** | OK | negbinomial.size | 0.000178625 | 0.00582 | RPL4 | TRUE |
| **HIF1A** | OK | negbinomial.size | 0.000179717 | 0.005847079 | HIF1A | TRUE |
| **ME2** | OK | negbinomial.size | 0.000181082 | 0.005882924 | ME2 | TRUE |
| **DHRS4L2** | OK | negbinomial.size | 0.000181632 | 0.005892242 | DHRS4L2 | TRUE |
| **SIRPB1** | OK | negbinomial.size | 0.000182117 | 0.00589943 | SIRPB1 | FALSE |
| **CAB39** | OK | negbinomial.size | 0.000182668 | 0.00590873 | CAB39 | TRUE |
| **RBMS1** | OK | negbinomial.size | 0.000184653 | 0.005964309 | RBMS1 | TRUE |
| **LAP3** | OK | negbinomial.size | 0.000184987 | 0.005966493 | LAP3 | TRUE |
| **BCAP31** | OK | negbinomial.size | 0.000185539 | 0.005975704 | BCAP31 | TRUE |
| **RPS25** | OK | negbinomial.size | 0.000187802 | 0.006039892 | RPS25 | TRUE |
| **GBP5** | OK | negbinomial.size | 0.000188265 | 0.006046084 | GBP5 | TRUE |
| **ITPKB** | OK | negbinomial.size | 0.000190098 | 0.006096218 | ITPKB | FALSE |
| **CTGF** | OK | negbinomial.size | 0.00019063 | 0.006104515 | CTGF | TRUE |
| **SHFM1** | OK | negbinomial.size | 0.000192628 | 0.006159697 | SHFM1 | TRUE |
| **GUK1** | OK | negbinomial.size | 0.000193116 | 0.006166475 | GUK1 | TRUE |
| **NKG7** | OK | negbinomial.size | 0.000197765 | 0.006305941 | NKG7 | TRUE |
| **NLRP3** | OK | negbinomial.size | 0.000199704 | 0.006358722 | NLRP3 | TRUE |
| **AURKB** | OK | negbinomial.size | 0.000201101 | 0.006394105 | AURKB | FALSE |
| **EPC1** | OK | negbinomial.size | 0.000202868 | 0.006441144 | EPC1 | TRUE |
| **CDC42SE1** | OK | negbinomial.size | 0.000208096 | 0.006597531 | CDC42SE1 | TRUE |
| **FGF7** | OK | negbinomial.size | 0.000208383 | 0.006597531 | FGF7 | FALSE |
| **ALDH2** | OK | negbinomial.size | 0.000210654 | 0.006660007 | ALDH2 | TRUE |
| **COPZ1** | OK | negbinomial.size | 0.000211153 | 0.006666355 | COPZ1 | TRUE |
| **REEP5** | OK | negbinomial.size | 0.000211571 | 0.006670157 | REEP5 | TRUE |
| **CD300A** | OK | negbinomial.size | 0.000213173 | 0.006711198 | CD300A | TRUE |
| **KCTD12** | OK | negbinomial.size | 0.000213722 | 0.00671905 | KCTD12 | TRUE |
| **ATP5J** | OK | negbinomial.size | 0.000216119 | 0.006784857 | ATP5J | TRUE |
| **CD93** | OK | negbinomial.size | 0.000216719 | 0.006794182 | CD93 | TRUE |
| **F11R** | OK | negbinomial.size | 0.000218778 | 0.006849134 | F11R | TRUE |
| **SPRY1** | OK | negbinomial.size | 0.000219427 | 0.00685986 | SPRY1 | TRUE |
| **CCR7** | OK | negbinomial.size | 0.000222345 | 0.006941386 | CCR7 | TRUE |
| **ARHGAP10** | OK | negbinomial.size | 0.000227727 | 0.007091152 | ARHGAP10 | FALSE |
| **CYP27B1** | OK | negbinomial.size | 0.000227831 | 0.007091152 | CYP27B1 | FALSE |
| **MCL1** | OK | negbinomial.size | 0.000228093 | 0.007091152 | MCL1 | TRUE |
| **COMT** | OK | negbinomial.size | 0.000228666 | 0.007099107 | COMT | TRUE |
| **SOAT1** | OK | negbinomial.size | 0.000232753 | 0.007215985 | SOAT1 | TRUE |
| **RPS29** | OK | negbinomial.size | 0.00023323 | 0.007220769 | RPS29 | TRUE |
| **PRPF38B** | OK | negbinomial.size | 0.000233665 | 0.007224247 | PRPF38B | TRUE |
| **NR3C1** | OK | negbinomial.size | 0.000240228 | 0.007416908 | NR3C1 | TRUE |
| **EEF1B2** | OK | negbinomial.size | 0.000240999 | 0.00743048 | EEF1B2 | TRUE |
| **DAGLB** | OK | negbinomial.size | 0.000241346 | 0.007430928 | DAGLB | TRUE |
| **RPL12** | OK | negbinomial.size | 0.000242483 | 0.007455672 | RPL12 | TRUE |
| **ENPP2** | OK | negbinomial.size | 0.000243933 | 0.007489966 | ENPP2 | TRUE |
| **CPVL** | OK | negbinomial.size | 0.000244621 | 0.007500825 | CPVL | TRUE |
| **MALT1** | OK | negbinomial.size | 0.000246689 | 0.007553866 | MALT1 | FALSE |
| **NDUFA13** | OK | negbinomial.size | 0.000248882 | 0.007610628 | NDUFA13 | TRUE |
| **SRSF3** | OK | negbinomial.size | 0.000249721 | 0.007625871 | SRSF3 | TRUE |
| **PLA2G2D** | OK | negbinomial.size | 0.000250777 | 0.007647665 | PLA2G2D | FALSE |
| **MSRB1** | OK | negbinomial.size | 0.000251683 | 0.007664868 | MSRB1 | TRUE |
| **KLF2** | OK | negbinomial.size | 0.000253177 | 0.007699869 | KLF2 | TRUE |
| **PHACTR4** | OK | negbinomial.size | 0.000256693 | 0.007796227 | PHACTR4 | TRUE |
| **RP11-473M20.16** | OK | negbinomial.size | 0.000259661 | 0.007875679 | RP11-473M20.16 | FALSE |
| **SEC14L1** | OK | negbinomial.size | 0.000261289 | 0.00791075 | SEC14L1 | TRUE |
| **EDF1** | OK | negbinomial.size | 0.000261524 | 0.00791075 | EDF1 | TRUE |
| **MTUS1** | OK | negbinomial.size | 0.000264944 | 0.008003368 | MTUS1 | TRUE |
| **CD14** | OK | negbinomial.size | 0.000267354 | 0.008065288 | CD14 | TRUE |
| **RPL15** | OK | negbinomial.size | 0.000267926 | 0.008071673 | RPL15 | TRUE |
| **SDHB** | OK | negbinomial.size | 0.00026876 | 0.008085917 | SDHB | TRUE |
| **IFIH1** | OK | negbinomial.size | 0.000272027 | 0.008173214 | IFIH1 | TRUE |
| **POMP** | OK | negbinomial.size | 0.000276043 | 0.008278235 | POMP | TRUE |
| **TACSTD2** | OK | negbinomial.size | 0.000276262 | 0.008278235 | TACSTD2 | FALSE |
| **RPL6** | OK | negbinomial.size | 0.000282098 | 0.008441821 | RPL6 | TRUE |
| **CD274** | OK | negbinomial.size | 0.000284639 | 0.008496109 | CD274 | FALSE |
| **MED30** | OK | negbinomial.size | 0.000285002 | 0.008496109 | MED30 | TRUE |
| **OAS1** | OK | negbinomial.size | 0.000285051 | 0.008496109 | OAS1 | TRUE |
| **MAP1LC3B** | OK | negbinomial.size | 0.000299936 | 0.008920545 | MAP1LC3B | TRUE |
| **FAM198B** | OK | negbinomial.size | 0.000300088 | 0.008920545 | FAM198B | TRUE |
| **HNRNPDL** | OK | negbinomial.size | 0.00030551 | 0.009069666 | HNRNPDL | TRUE |
| **LINC00116** | OK | negbinomial.size | 0.000310597 | 0.009208488 | LINC00116 | TRUE |
| **GPR137B** | OK | negbinomial.size | 0.000317339 | 0.009395928 | GPR137B | TRUE |
| **NDUFB3** | OK | negbinomial.size | 0.000321336 | 0.009501708 | NDUFB3 | TRUE |
| **TRBC1** | OK | negbinomial.size | 0.000323243 | 0.009541523 | TRBC1 | FALSE |
| **TNFSF13B** | OK | negbinomial.size | 0.000323535 | 0.009541523 | TNFSF13B | TRUE |
| **AASDHPPT** | OK | negbinomial.size | 0.000326158 | 0.009606199 | AASDHPPT | TRUE |
| **FBLN1** | OK | negbinomial.size | 0.000326774 | 0.009611709 | FBLN1 | FALSE |
| **BNIP3L** | OK | negbinomial.size | 0.00032756 | 0.009622178 | BNIP3L | TRUE |
| **GADD45GIP1** | OK | negbinomial.size | 0.000330781 | 0.009704077 | GADD45GIP1 | TRUE |
| **KCNN4** | OK | negbinomial.size | 0.000332626 | 0.009745431 | KCNN4 | FALSE |
| **BRI3** | OK | negbinomial.size | 0.000334745 | 0.009794683 | BRI3 | TRUE |
| **MDK** | OK | negbinomial.size | 0.000336838 | 0.009843052 | MDK | TRUE |
| **CCNB1** | OK | negbinomial.size | 0.000338066 | 0.009866056 | CCNB1 | FALSE |
| **CAPNS1** | OK | negbinomial.size | 0.00033913 | 0.009884231 | CAPNS1 | TRUE |
| **DDX24** | OK | negbinomial.size | 0.000340097 | 0.009899516 | DDX24 | TRUE |
| **ATF5** | OK | negbinomial.size | 0.000343854 | 0.00999588 | ATF5 | TRUE |
| **FKBP4** | OK | negbinomial.size | 0.000348981 | 0.010131778 | FKBP4 | TRUE |
| **OPN3** | OK | negbinomial.size | 0.000363293 | 0.010533605 | OPN3 | TRUE |
| **CD151** | OK | negbinomial.size | 0.0003641 | 0.010543343 | CD151 | TRUE |
| **CD99** | OK | negbinomial.size | 0.000366571 | 0.010601206 | CD99 | TRUE |
| **DNAJA4** | OK | negbinomial.size | 0.000369467 | 0.010671153 | DNAJA4 | TRUE |
| **TMEM39B** | OK | negbinomial.size | 0.000372147 | 0.010734716 | TMEM39B | FALSE |
| **LRRC75A** | OK | negbinomial.size | 0.000375983 | 0.010821674 | LRRC75A | FALSE |
| **ZFP36L1** | OK | negbinomial.size | 0.000376129 | 0.010821674 | ZFP36L1 | TRUE |
| **NFKBIZ** | OK | negbinomial.size | 0.000378706 | 0.010879319 | NFKBIZ | TRUE |
| **KRT18** | OK | negbinomial.size | 0.000379104 | 0.010879319 | KRT18 | TRUE |
| **C19orf53** | OK | negbinomial.size | 0.000383897 | 0.01100276 | C19orf53 | TRUE |
| **ARPC2** | OK | negbinomial.size | 0.000385256 | 0.011027581 | ARPC2 | TRUE |
| **NRIP3** | OK | negbinomial.size | 0.000387814 | 0.011086629 | NRIP3 | TRUE |
| **NDUFV2** | OK | negbinomial.size | 0.000391374 | 0.011174132 | NDUFV2 | TRUE |
| **PPDPF** | OK | negbinomial.size | 0.000392767 | 0.011199624 | PPDPF | TRUE |
| **C1orf35** | OK | negbinomial.size | 0.000398486 | 0.011348244 | C1orf35 | TRUE |
| **RIN2** | OK | negbinomial.size | 0.000403737 | 0.011483155 | RIN2 | TRUE |
| **MDFIC** | OK | negbinomial.size | 0.000404352 | 0.011486059 | MDFIC | TRUE |
| **KRT17** | OK | negbinomial.size | 0.000412223 | 0.011684289 | KRT17 | TRUE |
| **SIRPA** | OK | negbinomial.size | 0.000412374 | 0.011684289 | SIRPA | TRUE |
| **GPX4** | OK | negbinomial.size | 0.000415109 | 0.011746899 | GPX4 | TRUE |
| **PLGRKT** | OK | negbinomial.size | 0.000417594 | 0.01180231 | PLGRKT | TRUE |
| **RPL14** | OK | negbinomial.size | 0.000429373 | 0.012119907 | RPL14 | TRUE |
| **SH3GLB1** | OK | negbinomial.size | 0.000431883 | 0.012175399 | SH3GLB1 | TRUE |
| **PILRA** | OK | negbinomial.size | 0.000436599 | 0.012292863 | PILRA | TRUE |
| **KRT14** | OK | negbinomial.size | 0.000443868 | 0.012481847 | KRT14 | FALSE |
| **MXD1** | OK | negbinomial.size | 0.000445875 | 0.012522552 | MXD1 | TRUE |
| **RAB10** | OK | negbinomial.size | 0.000446792 | 0.01253258 | RAB10 | TRUE |
| **JMJD6** | OK | negbinomial.size | 0.00045249 | 0.01266683 | JMJD6 | TRUE |
| **BCAS2** | OK | negbinomial.size | 0.00045295 | 0.01266683 | BCAS2 | TRUE |
| **GIMAP1** | OK | negbinomial.size | 0.000453276 | 0.01266683 | GIMAP1 | TRUE |
| **SMG1** | OK | negbinomial.size | 0.00045695 | 0.012753575 | SMG1 | TRUE |
| **SAMSN1** | OK | negbinomial.size | 0.000465216 | 0.012968125 | SAMSN1 | TRUE |
| **PYCARD** | OK | negbinomial.size | 0.000468488 | 0.013032999 | PYCARD | TRUE |
| **TBXAS1** | OK | negbinomial.size | 0.000468708 | 0.013032999 | TBXAS1 | TRUE |
| **GLUL** | OK | negbinomial.size | 0.000476015 | 0.013219748 | GLUL | TRUE |
| **CLEC5A** | OK | negbinomial.size | 0.000478694 | 0.013277689 | CLEC5A | FALSE |
| **SKI** | OK | negbinomial.size | 0.000482978 | 0.013379926 | SKI | TRUE |
| **CDKN1A** | OK | negbinomial.size | 0.000485559 | 0.013419853 | CDKN1A | TRUE |
| **3-Mar** | OK | negbinomial.size | 0.000485965 | 0.013419853 | 3-Mar | FALSE |
| **ARHGDIB** | OK | negbinomial.size | 0.000486316 | 0.013419853 | ARHGDIB | TRUE |
| **LEPROTL1** | OK | negbinomial.size | 0.000486817 | 0.013419853 | LEPROTL1 | TRUE |
| **TMEM208** | OK | negbinomial.size | 0.000491599 | 0.013535 | TMEM208 | TRUE |
| **FCGRT** | OK | negbinomial.size | 0.00049543 | 0.013623725 | FCGRT | TRUE |
| **SUCNR1** | OK | negbinomial.size | 0.000504924 | 0.013867763 | SUCNR1 | FALSE |
| **COX7B** | OK | negbinomial.size | 0.000517162 | 0.014186477 | COX7B | TRUE |
| **COX4I1** | OK | negbinomial.size | 0.000519577 | 0.014235261 | COX4I1 | TRUE |
| **LYN** | OK | negbinomial.size | 0.000526867 | 0.014411483 | LYN | TRUE |
| **TMCO3** | OK | negbinomial.size | 0.000527297 | 0.014411483 | TMCO3 | TRUE |
| **SLC25A6** | OK | negbinomial.size | 0.000529303 | 0.014448681 | SLC25A6 | TRUE |
| **PKIB** | OK | negbinomial.size | 0.000531258 | 0.014484387 | PKIB | TRUE |
| **CCRL2** | OK | negbinomial.size | 0.000538553 | 0.014665403 | CCRL2 | TRUE |
| **PIGR** | OK | negbinomial.size | 0.000542799 | 0.014763084 | PIGR | FALSE |
| **COX8A** | OK | negbinomial.size | 0.000555841 | 0.015099458 | COX8A | TRUE |
| **EXOC1** | OK | negbinomial.size | 0.000574322 | 0.015582581 | EXOC1 | TRUE |
| **ZNF812P** | OK | negbinomial.size | 0.00057637 | 0.015619218 | ZNF812P | FALSE |
| **NDUFB2** | OK | negbinomial.size | 0.000578989 | 0.01567121 | NDUFB2 | TRUE |
| **MAF** | OK | negbinomial.size | 0.000579876 | 0.015676256 | MAF | TRUE |
| **DHRS3** | OK | negbinomial.size | 0.000584908 | 0.015793232 | DHRS3 | TRUE |
| **CBR1** | OK | negbinomial.size | 0.000587363 | 0.015840397 | CBR1 | TRUE |
| **ITM2C** | OK | negbinomial.size | 0.000589766 | 0.015886062 | ITM2C | TRUE |
| **ADAM9** | OK | negbinomial.size | 0.000593536 | 0.015968398 | ADAM9 | TRUE |
| **MERTK** | OK | negbinomial.size | 0.000594525 | 0.015975816 | MERTK | TRUE |
| **C1orf162** | OK | negbinomial.size | 0.000595524 | 0.015983459 | C1orf162 | TRUE |
| **CECR1** | OK | negbinomial.size | 0.000610577 | 0.016367847 | CECR1 | TRUE |
| **HEXB** | OK | negbinomial.size | 0.000615439 | 0.016478461 | HEXB | TRUE |
| **CCDC59** | OK | negbinomial.size | 0.000625037 | 0.01671544 | CCDC59 | TRUE |
| **NFKB1** | OK | negbinomial.size | 0.000631738 | 0.016874483 | NFKB1 | TRUE |
| **SCPEP1** | OK | negbinomial.size | 0.000632688 | 0.016879735 | SCPEP1 | TRUE |
| **PDGFRL** | OK | negbinomial.size | 0.000639372 | 0.017037748 | PDGFRL | FALSE |
| **FOXN2** | OK | negbinomial.size | 0.000641017 | 0.017061271 | FOXN2 | TRUE |
| **S100A7** | OK | negbinomial.size | 0.000646106 | 0.017176287 | S100A7 | FALSE |
| **TOR3A** | OK | negbinomial.size | 0.000654566 | 0.017380558 | TOR3A | TRUE |
| **DICER1** | OK | negbinomial.size | 0.000669605 | 0.017758808 | DICER1 | TRUE |
| **RSU1** | OK | negbinomial.size | 0.000675466 | 0.017893047 | RSU1 | TRUE |
| **RSAD2** | OK | negbinomial.size | 0.000678519 | 0.017952685 | RSAD2 | TRUE |
| **LYVE1** | OK | negbinomial.size | 0.000682597 | 0.018039265 | LYVE1 | FALSE |
| **FCGR2B** | OK | negbinomial.size | 0.000686116 | 0.018110867 | FCGR2B | TRUE |
| **TMEM154** | OK | negbinomial.size | 0.000705592 | 0.018603018 | TMEM154 | FALSE |
| **RND3** | OK | negbinomial.size | 0.000710987 | 0.018723218 | RND3 | TRUE |
| **ATP2B1** | OK | negbinomial.size | 0.000724139 | 0.019047163 | ATP2B1 | TRUE |
| **TTC39B** | OK | negbinomial.size | 0.000727609 | 0.019115958 | TTC39B | FALSE |
| **MRPL41** | OK | negbinomial.size | 0.000737585 | 0.019355326 | MRPL41 | TRUE |
| **SF3B6** | OK | negbinomial.size | 0.000749434 | 0.019643257 | SF3B6 | TRUE |
| **CPM** | OK | negbinomial.size | 0.00075286 | 0.019709972 | CPM | TRUE |
| **RNF213** | OK | negbinomial.size | 0.000755755 | 0.019762652 | RNF213 | TRUE |
| **SAA1** | OK | negbinomial.size | 0.000756991 | 0.019771853 | SAA1 | TRUE |
| **FOXN3** | OK | negbinomial.size | 0.000758383 | 0.019785138 | FOXN3 | TRUE |
| **P2RY14** | OK | negbinomial.size | 0.000760772 | 0.019824349 | P2RY14 | FALSE |
| **NDUFA12** | OK | negbinomial.size | 0.000762299 | 0.019831257 | NDUFA12 | TRUE |
| **LRRC61** | OK | negbinomial.size | 0.000762809 | 0.019831257 | LRRC61 | FALSE |
| **INTS1** | OK | negbinomial.size | 0.000765189 | 0.019870064 | INTS1 | FALSE |
| **MZT2B** | OK | negbinomial.size | 0.000769739 | 0.019965041 | MZT2B | TRUE |
| **ITM2B** | OK | negbinomial.size | 0.000781089 | 0.020212613 | ITM2B | TRUE |
| **ECHS1** | OK | negbinomial.size | 0.00078109 | 0.020212613 | ECHS1 | TRUE |
| **RPL32** | OK | negbinomial.size | 0.000809374 | 0.020899105 | RPL32 | TRUE |
| **CLEC4A** | OK | negbinomial.size | 0.000809486 | 0.020899105 | CLEC4A | TRUE |
| **GNB2L1** | OK | negbinomial.size | 0.000810434 | 0.020899476 | GNB2L1 | TRUE |
| **IGFBP4** | OK | negbinomial.size | 0.000817773 | 0.021064487 | IGFBP4 | TRUE |
| **PTX3** | OK | negbinomial.size | 0.000820745 | 0.02110527 | PTX3 | FALSE |
| **AXL** | OK | negbinomial.size | 0.000821242 | 0.02110527 | AXL | TRUE |
| **NDUFB4** | OK | negbinomial.size | 0.000824674 | 0.021150632 | NDUFB4 | TRUE |
| **CCL23** | OK | negbinomial.size | 0.00082561 | 0.021150632 | CCL23 | FALSE |
| **RPL41** | OK | negbinomial.size | 0.000825842 | 0.021150632 | RPL41 | TRUE |
| **CHMP2A** | OK | negbinomial.size | 0.000828889 | 0.021202856 | CHMP2A | TRUE |
| **COX17** | OK | negbinomial.size | 0.000829776 | 0.021202856 | COX17 | TRUE |
| **PHLDA3** | OK | negbinomial.size | 0.000835047 | 0.021313221 | PHLDA3 | FALSE |
| **CEP170** | OK | negbinomial.size | 0.000840667 | 0.021429794 | CEP170 | TRUE |
| **FBXO6** | OK | negbinomial.size | 0.000841529 | 0.021429794 | FBXO6 | TRUE |
| **GSN** | OK | negbinomial.size | 0.000850587 | 0.021635829 | GSN | TRUE |
| **GEM** | OK | negbinomial.size | 0.000854587 | 0.021712917 | GEM | TRUE |
| **COX6A1** | OK | negbinomial.size | 0.000860011 | 0.021825935 | COX6A1 | TRUE |
| **TRMT61A** | OK | negbinomial.size | 0.00086409 | 0.02190463 | TRMT61A | FALSE |
| **ST3GAL6** | OK | negbinomial.size | 0.00087168 | 0.02207204 | ST3GAL6 | TRUE |
| **MT1X** | OK | negbinomial.size | 0.00087782 | 0.022202399 | MT1X | TRUE |
| **SRD5A3** | OK | negbinomial.size | 0.000901561 | 0.022777126 | SRD5A3 | TRUE |
| **LFNG** | OK | negbinomial.size | 0.000904582 | 0.022827689 | LFNG | FALSE |
| **AVPI1** | OK | negbinomial.size | 0.000912031 | 0.022963788 | AVPI1 | TRUE |
| **CAPS** | OK | negbinomial.size | 0.000912607 | 0.022963788 | CAPS | TRUE |
| **RPL30** | OK | negbinomial.size | 0.000913053 | 0.022963788 | RPL30 | TRUE |
| **NKAP** | OK | negbinomial.size | 0.0009247 | 0.023230621 | NKAP | TRUE |
| **ADPRM** | OK | negbinomial.size | 0.000926063 | 0.023238772 | ADPRM | FALSE |
| **STX11** | OK | negbinomial.size | 0.000938481 | 0.023524024 | STX11 | TRUE |
| **TOP2A** | OK | negbinomial.size | 0.000944955 | 0.023659801 | TOP2A | FALSE |
| **FAM210A** | OK | negbinomial.size | 0.000953515 | 0.023847456 | FAM210A | TRUE |
| **CADM1** | OK | negbinomial.size | 0.00097337 | 0.024316871 | CADM1 | TRUE |
| **MCOLN3** | OK | negbinomial.size | 0.000982828 | 0.02452577 | MCOLN3 | FALSE |
| **ACTR2** | OK | negbinomial.size | 0.000991811 | 0.024722373 | ACTR2 | TRUE |
| **TK1** | OK | negbinomial.size | 0.000996049 | 0.024800407 | TK1 | FALSE |
| **ZNF90** | OK | negbinomial.size | 0.00100224 | 0.024926811 | ZNF90 | FALSE |
| **CALML5** | OK | negbinomial.size | 0.001004528 | 0.024955994 | CALML5 | TRUE |
| **RPL27A** | OK | negbinomial.size | 0.001008114 | 0.024995804 | RPL27A | TRUE |
| **ITGAV** | OK | negbinomial.size | 0.001008364 | 0.024995804 | ITGAV | TRUE |
| **SLC7A8** | OK | negbinomial.size | 0.001012857 | 0.025079422 | SLC7A8 | TRUE |
| **SCAMP3** | OK | negbinomial.size | 0.001015028 | 0.025084539 | SCAMP3 | TRUE |
| **S100P** | OK | negbinomial.size | 0.001015305 | 0.025084539 | S100P | FALSE |
| **OSER1** | OK | negbinomial.size | 0.001022255 | 0.025228402 | OSER1 | TRUE |
| **CLDN5** | OK | negbinomial.size | 0.001024459 | 0.02525494 | CLDN5 | FALSE |
| **ADM** | OK | negbinomial.size | 0.001028429 | 0.025324927 | ADM | TRUE |
| **HTATIP2** | OK | negbinomial.size | 0.001034292 | 0.025441316 | HTATIP2 | TRUE |
| **TNFRSF4** | OK | negbinomial.size | 0.001037292 | 0.025485243 | TNFRSF4 | FALSE |
| **DDX60L** | OK | negbinomial.size | 0.001038355 | 0.025485243 | DDX60L | TRUE |
| **ATP6V0E1** | OK | negbinomial.size | 0.001049454 | 0.025729433 | ATP6V0E1 | TRUE |
| **ATP1B3** | OK | negbinomial.size | 0.001076604 | 0.026366208 | ATP1B3 | TRUE |
| **OFD1** | OK | negbinomial.size | 0.001079029 | 0.026396697 | OFD1 | TRUE |
| **ABHD11** | OK | negbinomial.size | 0.001085414 | 0.026523926 | ABHD11 | TRUE |
| **UBC** | OK | negbinomial.size | 0.001091558 | 0.02664496 | UBC | TRUE |
| **CEACAM4** | OK | negbinomial.size | 0.001093337 | 0.026659317 | CEACAM4 | FALSE |
| **RHOH** | OK | negbinomial.size | 0.001099606 | 0.026767207 | RHOH | TRUE |
| **HNRNPA0** | OK | negbinomial.size | 0.001100153 | 0.026767207 | HNRNPA0 | TRUE |
| **SERINC1** | OK | negbinomial.size | 0.00110243 | 0.026768908 | SERINC1 | TRUE |
| **PREX1** | OK | negbinomial.size | 0.001102615 | 0.026768908 | PREX1 | TRUE |
| **AIF1** | OK | negbinomial.size | 0.001104302 | 0.026780826 | AIF1 | TRUE |
| **TBC1D12** | OK | negbinomial.size | 0.001114449 | 0.026997639 | TBC1D12 | FALSE |
| **DNAJB4** | OK | negbinomial.size | 0.001122388 | 0.027160568 | DNAJB4 | TRUE |
| **CARS2** | OK | negbinomial.size | 0.001131832 | 0.027347994 | CARS2 | FALSE |
| **MMP7** | OK | negbinomial.size | 0.001132576 | 0.027347994 | MMP7 | FALSE |
| **USP8** | OK | negbinomial.size | 0.00113879 | 0.027468403 | USP8 | TRUE |
| **HVCN1** | OK | negbinomial.size | 0.001148326 | 0.027668602 | HVCN1 | TRUE |
| **SLC6A6** | OK | negbinomial.size | 0.001157538 | 0.027860565 | SLC6A6 | TRUE |
| **GTF3C6** | OK | negbinomial.size | 0.001165249 | 0.028016041 | GTF3C6 | TRUE |
| **SAMHD1** | OK | negbinomial.size | 0.001171716 | 0.028141296 | SAMHD1 | TRUE |
| **GSDMD** | OK | negbinomial.size | 0.001183568 | 0.028395484 | GSDMD | TRUE |
| **EPB41L4A-AS1** | OK | negbinomial.size | 0.001187186 | 0.028451796 | EPB41L4A-AS1 | TRUE |
| **GOLGA7** | OK | negbinomial.size | 0.001199008 | 0.028685087 | GOLGA7 | TRUE |
| **EIF2S2** | OK | negbinomial.size | 0.001199484 | 0.028685087 | EIF2S2 | TRUE |
| **NDUFA3** | OK | negbinomial.size | 0.001209352 | 0.02886505 | NDUFA3 | TRUE |
| **TRA2A** | OK | negbinomial.size | 0.001209588 | 0.02886505 | TRA2A | TRUE |
| **TNFAIP6** | OK | negbinomial.size | 0.001212088 | 0.028866874 | TNFAIP6 | TRUE |
| **CLEC2B** | OK | negbinomial.size | 0.001212244 | 0.028866874 | CLEC2B | TRUE |
| **SLC15A4** | OK | negbinomial.size | 0.001219037 | 0.028995493 | SLC15A4 | TRUE |
| **CTH** | OK | negbinomial.size | 0.001220236 | 0.028995493 | CTH | FALSE |
| **MRPL27** | OK | negbinomial.size | 0.001222488 | 0.029018219 | MRPL27 | TRUE |
| **GNPTAB** | OK | negbinomial.size | 0.001225349 | 0.029032107 | GNPTAB | TRUE |
| **ZNF664** | OK | negbinomial.size | 0.001225667 | 0.029032107 | ZNF664 | FALSE |
| **GATM** | OK | negbinomial.size | 0.00123301 | 0.029165633 | GATM | TRUE |
| **FOSL2** | OK | negbinomial.size | 0.001233911 | 0.029165633 | FOSL2 | TRUE |
| **YAF2** | OK | negbinomial.size | 0.001235297 | 0.029167601 | YAF2 | TRUE |
| **CCT5** | OK | negbinomial.size | 0.001255052 | 0.029602829 | CCT5 | TRUE |
| **SDC4** | OK | negbinomial.size | 0.001258606 | 0.029655417 | SDC4 | TRUE |
| **FAM133B** | OK | negbinomial.size | 0.001261674 | 0.029696437 | FAM133B | TRUE |
| **HEXA-AS1** | OK | negbinomial.size | 0.001264601 | 0.029734073 | HEXA-AS1 | FALSE |
| **RGS13** | OK | negbinomial.size | 0.001274843 | 0.029893078 | RGS13 | FALSE |
| **MTMR10** | OK | negbinomial.size | 0.001275022 | 0.029893078 | MTMR10 | FALSE |
| **PITPNA** | OK | negbinomial.size | 0.00127537 | 0.029893078 | PITPNA | TRUE |
| **ZFHX3** | OK | negbinomial.size | 0.001277367 | 0.029908557 | ZFHX3 | TRUE |
| **CYC1** | OK | negbinomial.size | 0.001284505 | 0.030044255 | CYC1 | TRUE |
| **PDGFRA** | OK | negbinomial.size | 0.001307096 | 0.030540757 | PDGFRA | FALSE |
| **AZIN1** | OK | negbinomial.size | 0.00133671 | 0.031200125 | AZIN1 | TRUE |
| **WASF2** | OK | negbinomial.size | 0.001361575 | 0.031747393 | WASF2 | TRUE |
| **TPI1** | OK | negbinomial.size | 0.00137222 | 0.031962308 | TPI1 | TRUE |
| **RPLP2** | OK | negbinomial.size | 0.001380858 | 0.032130079 | RPLP2 | TRUE |
| **SAP30** | OK | negbinomial.size | 0.001383862 | 0.032166521 | SAP30 | TRUE |
| **QPCT** | OK | negbinomial.size | 0.0013949 | 0.032389451 | QPCT | FALSE |
| **ZNF394** | OK | negbinomial.size | 0.001398741 | 0.032444991 | ZNF394 | TRUE |
| **CAPN2** | OK | negbinomial.size | 0.001415764 | 0.0327462 | CAPN2 | TRUE |
| **TFF3** | OK | negbinomial.size | 0.001416115 | 0.0327462 | TFF3 | FALSE |
| **IGHG4** | OK | negbinomial.size | 0.001416115 | 0.0327462 | IGHG4 | FALSE |
| **MIR4435-2HG** | OK | negbinomial.size | 0.001435879 | 0.033168959 | MIR4435-2HG | TRUE |
| **VOPP1** | OK | negbinomial.size | 0.001443944 | 0.033320875 | VOPP1 | TRUE |
| **SYVN1** | OK | negbinomial.size | 0.001463903 | 0.033743102 | SYVN1 | FALSE |
| **CD302** | OK | negbinomial.size | 0.001466679 | 0.033743102 | CD302 | TRUE |
| **NUPR2** | OK | negbinomial.size | 0.00146716 | 0.033743102 | NUPR2 | FALSE |
| **SEC24A** | OK | negbinomial.size | 0.00146906 | 0.033743102 | SEC24A | FALSE |
| **LAMP2** | OK | negbinomial.size | 0.001469779 | 0.033743102 | LAMP2 | TRUE |
| **CNIH4** | OK | negbinomial.size | 0.001480179 | 0.033947049 | CNIH4 | TRUE |
| **HTRA1** | OK | negbinomial.size | 0.001486343 | 0.034049447 | HTRA1 | TRUE |
| **MYLK** | OK | negbinomial.size | 0.001487686 | 0.034049447 | MYLK | FALSE |
| **TNS1** | OK | negbinomial.size | 0.001492566 | 0.034126239 | TNS1 | FALSE |
| **CYSTM1** | OK | negbinomial.size | 0.001498773 | 0.034233194 | CYSTM1 | TRUE |
| **BASP1** | OK | negbinomial.size | 0.001502671 | 0.034274257 | BASP1 | TRUE |
| **ZBTB7A** | OK | negbinomial.size | 0.001503633 | 0.034274257 | ZBTB7A | TRUE |
| **PIGC** | OK | negbinomial.size | 0.001506585 | 0.034287221 | PIGC | TRUE |
| **SOD1** | OK | negbinomial.size | 0.001507265 | 0.034287221 | SOD1 | TRUE |
| **SNN** | OK | negbinomial.size | 0.00152044 | 0.03455181 | SNN | TRUE |
| **TCIRG1** | OK | negbinomial.size | 0.001544761 | 0.035068895 | TCIRG1 | TRUE |
| **AQP1** | OK | negbinomial.size | 0.001548974 | 0.035128907 | AQP1 | FALSE |
| **CACYBP** | OK | negbinomial.size | 0.001556483 | 0.035263481 | CACYBP | TRUE |
| **CCBL2** | OK | negbinomial.size | 0.001568838 | 0.035507456 | CCBL2 | FALSE |
| **ATP5I** | OK | negbinomial.size | 0.001575662 | 0.03562587 | ATP5I | TRUE |
| **SPRED1** | OK | negbinomial.size | 0.001577905 | 0.035640591 | SPRED1 | TRUE |
| **ETV3** | OK | negbinomial.size | 0.001615057 | 0.036442983 | ETV3 | TRUE |
| **YBX3** | OK | negbinomial.size | 0.001624401 | 0.036616908 | YBX3 | TRUE |
| **MAP2K3** | OK | negbinomial.size | 0.001647432 | 0.037098703 | MAP2K3 | TRUE |
| **INHBA** | OK | negbinomial.size | 0.00165181 | 0.037130881 | INHBA | FALSE |
| **HOMER1** | OK | negbinomial.size | 0.001652178 | 0.037130881 | HOMER1 | FALSE |
| **JAML** | OK | negbinomial.size | 0.001658857 | 0.037243595 | JAML | TRUE |
| **AP3S1** | OK | negbinomial.size | 0.001662314 | 0.037283799 | AP3S1 | TRUE |
| **CFD** | OK | negbinomial.size | 0.001668016 | 0.037374251 | CFD | TRUE |
| **IGSF21** | OK | negbinomial.size | 0.001685427 | 0.037726597 | IGSF21 | FALSE |
| **PITHD1** | OK | negbinomial.size | 0.001693152 | 0.037861643 | PITHD1 | TRUE |
| **BIRC3** | OK | negbinomial.size | 0.001700611 | 0.037990486 | BIRC3 | TRUE |
| **RPA3** | OK | negbinomial.size | 0.001715761 | 0.03829072 | RPA3 | TRUE |
| **AOAH** | OK | negbinomial.size | 0.001736568 | 0.038716462 | AOAH | TRUE |
| **COL14A1** | OK | negbinomial.size | 0.00174069 | 0.038731244 | COL14A1 | FALSE |
| **GLT8D2** | OK | negbinomial.size | 0.001740691 | 0.038731244 | GLT8D2 | FALSE |
| **DPM1** | OK | negbinomial.size | 0.001747734 | 0.038824665 | DPM1 | TRUE |
| **PSMD7** | OK | negbinomial.size | 0.001749631 | 0.038824665 | PSMD7 | TRUE |
| **SEC23IP** | OK | negbinomial.size | 0.001751266 | 0.038824665 | SEC23IP | FALSE |
| **HBA1** | OK | negbinomial.size | 0.001753278 | 0.038824665 | HBA1 | FALSE |
| **AES** | OK | negbinomial.size | 0.001753562 | 0.038824665 | AES | TRUE |
| **RANBP1** | OK | negbinomial.size | 0.001762783 | 0.038990254 | RANBP1 | TRUE |
| **ARID5A** | OK | negbinomial.size | 0.00177327 | 0.039119065 | ARID5A | TRUE |
| **TMEM173** | OK | negbinomial.size | 0.001774075 | 0.039119065 | TMEM173 | TRUE |
| **GRASP** | OK | negbinomial.size | 0.001775061 | 0.039119065 | GRASP | TRUE |
| **PRMT9** | OK | negbinomial.size | 0.001775597 | 0.039119065 | PRMT9 | TRUE |
| **PSMB1** | OK | negbinomial.size | 0.001781722 | 0.039215396 | PSMB1 | TRUE |
| **NEIL2** | OK | negbinomial.size | 0.001793688 | 0.039439986 | NEIL2 | FALSE |
| **AHCYL1** | OK | negbinomial.size | 0.001803067 | 0.039540673 | AHCYL1 | TRUE |
| **COX7A2** | OK | negbinomial.size | 0.001803267 | 0.039540673 | COX7A2 | TRUE |
| **PSTPIP2** | OK | negbinomial.size | 0.001803566 | 0.039540673 | PSTPIP2 | TRUE |
| **MRPS18C** | OK | negbinomial.size | 0.001824254 | 0.039955082 | MRPS18C | TRUE |
| **TXNDC17** | OK | negbinomial.size | 0.001834312 | 0.040136112 | TXNDC17 | TRUE |
| **SOS 1** | OK | negbinomial.size | 0.001857491 | 0.040603595 | SOS 1 | FALSE |
| **NUB1** | OK | negbinomial.size | 0.001864759 | 0.040722701 | NUB1 | TRUE |
| **NENF** | OK | negbinomial.size | 0.001877166 | 0.040884705 | NENF | TRUE |
| **SIDT1** | OK | negbinomial.size | 0.001877271 | 0.040884705 | SIDT1 | FALSE |
| **MMP24-AS1** | OK | negbinomial.size | 0.001877657 | 0.040884705 | MMP24-AS1 | FALSE |
| **FKBP2** | OK | negbinomial.size | 0.00188413 | 0.040985774 | FKBP2 | TRUE |
| **SHTN1** | OK | negbinomial.size | 0.001891518 | 0.041077818 | SHTN1 | TRUE |
| **ELK3** | OK | negbinomial.size | 0.001892031 | 0.041077818 | ELK3 | FALSE |
| **UBXN1** | OK | negbinomial.size | 0.001899262 | 0.041194849 | UBXN1 | TRUE |
| **COX5A** | OK | negbinomial.size | 0.001910205 | 0.041392091 | COX5A | TRUE |
| **MREG** | OK | negbinomial.size | 0.001917953 | 0.041509907 | MREG | FALSE |
| **YWHAZ** | OK | negbinomial.size | 0.001919351 | 0.041509907 | YWHAZ | TRUE |
| **CKAP4** | OK | negbinomial.size | 0.00194418 | 0.042006302 | CKAP4 | FALSE |
| **INSIG1** | OK | negbinomial.size | 0.00195364 | 0.042169989 | INSIG1 | TRUE |
| **C3AR1** | OK | negbinomial.size | 0.001970929 | 0.042502189 | C3AR1 | TRUE |
| **SRI** | OK | negbinomial.size | 0.001975989 | 0.042570297 | SRI | TRUE |
| **DOCK7** | OK | negbinomial.size | 0.001979782 | 0.042611008 | DOCK7 | FALSE |
| **RASSF5** | OK | negbinomial.size | 0.001982329 | 0.042624826 | RASSF5 | TRUE |
| **NDUFB9** | OK | negbinomial.size | 0.002000311 | 0.042970222 | NDUFB9 | TRUE |
| **PPP3R1** | OK | negbinomial.size | 0.00200563 | 0.043043169 | PPP3R1 | TRUE |
| **AGTPBP1** | OK | negbinomial.size | 0.002008339 | 0.043060017 | AGTPBP1 | FALSE |
| **TAF7** | OK | negbinomial.size | 0.002011499 | 0.043086494 | TAF7 | TRUE |
| **PTPN6** | OK | negbinomial.size | 0.002043184 | 0.043723352 | PTPN6 | TRUE |
| **PLEKHO1** | OK | negbinomial.size | 0.002045143 | 0.043723484 | PLEKHO1 | TRUE |
| **CLEC7A** | OK | negbinomial.size | 0.002050076 | 0.043787127 | CLEC7A | TRUE |
| **SLC18B1** | OK | negbinomial.size | 0.002054071 | 0.043804565 | SLC18B1 | TRUE |
| **RPL10A** | OK | negbinomial.size | 0.002054807 | 0.043804565 | RPL10A | TRUE |
| **PSMA7** | OK | negbinomial.size | 0.002069097 | 0.044067229 | PSMA7 | TRUE |
| **TES** | OK | negbinomial.size | 0.002078055 | 0.044215948 | TES | TRUE |
| **FNDC3B** | OK | negbinomial.size | 0.002086671 | 0.044357112 | FNDC3B | TRUE |
| **PDE8A** | OK | negbinomial.size | 0.002101671 | 0.044633587 | PDE8A | TRUE |
| **PGAM1** | OK | negbinomial.size | 0.002109571 | 0.044758899 | PGAM1 | TRUE |
| **SBDS** | OK | negbinomial.size | 0.002119047 | 0.044917376 | SBDS | TRUE |
| **EIF4E** | OK | negbinomial.size | 0.002122797 | 0.044954283 | EIF4E | TRUE |
| **UBE2M** | OK | negbinomial.size | 0.002143841 | 0.045357028 | UBE2M | TRUE |
| **TFPT** | OK | negbinomial.size | 0.002148366 | 0.045409851 | TFPT | TRUE |
| **SVBP** | OK | negbinomial.size | 0.002164633 | 0.045710522 | SVBP | TRUE |
| **TBCC** | OK | negbinomial.size | 0.002174989 | 0.045843038 | TBCC | TRUE |
| **TAGAP** | OK | negbinomial.size | 0.002175005 | 0.045843038 | TAGAP | TRUE |
| **CBX3** | OK | negbinomial.size | 0.002179928 | 0.045903578 | CBX3 | TRUE |
| **RP11-1094M14.11** | OK | negbinomial.size | 0.002189948 | 0.046071246 | RP11-1094M14.11 | TRUE |
| **SLC3A2** | OK | negbinomial.size | 0.002234056 | 0.046955039 | SLC3A2 | TRUE |
| **ANPEP** | OK | negbinomial.size | 0.002249589 | 0.047218881 | ANPEP | TRUE |
| **SDHD** | OK | negbinomial.size | 0.002250829 | 0.047218881 | SDHD | TRUE |
| **FCGR1B** | OK | negbinomial.size | 0.00227136 | 0.047604979 | FCGR1B | TRUE |
| **CANX** | OK | negbinomial.size | 0.002277518 | 0.047689395 | CANX | TRUE |
| **SNAPC1** | OK | negbinomial.size | 0.002298603 | 0.048085924 | SNAPC1 | TRUE |
| **RFK** | OK | negbinomial.size | 0.002305834 | 0.048192156 | RFK | FALSE |
| **H2AFY2** | OK | negbinomial.size | 0.002329973 | 0.048651229 | H2AFY2 | FALSE |
| **EPB41L2** | OK | negbinomial.size | 0.002339041 | 0.04879506 | EPB41L2 | TRUE |
| **UBE2D3** | OK | negbinomial.size | 0.002348171 | 0.048939911 | UBE2D3 | TRUE |
| **RPL38** | OK | negbinomial.size | 0.002371369 | 0.049377422 | RPL38 | TRUE |
| **CD109** | OK | negbinomial.size | 0.002399337 | 0.049913339 | CD109 | FALSE |
