# Supplementary material for: Integrating Bulk and Single-cell RNA-seq to Construct a Macrophage-related Prognostic Model for Prognostic Stratification in Triple-negative Breast Cancer
Source: J Cancer. 2024 Sep 23;15(18):6002–15. doi: 10.7150/jca.101042 (PMC11493015; doi:10.7150/jca.101042)
Supplement: Supplementary file 1 — Supplementary figure and tables. [file jcav15p6002s1.zip › Supplementary File/Table S4 The results of multivariate COX regression.docx]

| **Table S4 The results of multivariate COX regression** | | | | | |
| --- | --- | --- | --- | --- | --- |
|  | coef | exp(coef) | se(coef) | z | Pr(>\|z\|) |
| **HSPA6** | -0.204042148 | 0.815428001 | 0.1160167 | -1.75873084 | 0.078623237 |
| **LPL** | -0.12889278 | 0.879068214 | 0.07963716 | -1.618500468 | 0.105554786 |
| **IDO1** | -0.162297419 | 0.850188305 | 0.071692056 | -2.263813161 | 0.023585609 |
| **ALDH2** | -0.143447144 | 0.866366591 | 0.09767212 | -1.468660092 | 0.141925007 |
| **TK1** | 0.214092012 | 1.238736628 | 0.09771118 | 2.191069761 | 0.028446745 |
| **QPCT** | -0.130609533 | 0.877560366 | 0.067141613 | -1.945284411 | 0.051740757 |
