# Supplementary material for: Integrating Bulk and Single-cell RNA-seq to Construct a Macrophage-related Prognostic Model for Prognostic Stratification in Triple-negative Breast Cancer
Source: J Cancer. 2024 Sep 23;15(18):6002–15. doi: 10.7150/jca.101042 (PMC11493015; doi:10.7150/jca.101042)
Supplement: Supplementary file 1 — Supplementary figure and tables. [file jcav15p6002s1.zip › Supplementary File/Table S5 The differential expression analysis of high.docx]

| **Table S5 The differential expression analysis of high- and low- risk groups in METABRIC dataset** | | |
| --- | --- | --- |
| **gene name** | logFC | P.Value |
| **CXCL8** | 0.422287975 | 0.023085492 |
| **H19** | 0.411821984 | 0.023238284 |
| **STAC2** | 0.378022118 | 0.01142138 |
| **TNC** | 0.374218181 | 0.003278887 |
| **SERPINE2** | 0.327325923 | 0.014332227 |
| **SNRPN** | 0.321318249 | 0.003330357 |
| **TCEAL2** | 0.31800856 | 0.005603909 |
| **TMEM98** | 0.299011168 | 0.010831788 |
| **EGR1** | 0.27668787 | 0.023955435 |
| **IRX2** | 0.260163677 | 0.042197441 |
| **C1QTNF1** | 0.258582564 | 0.012971648 |
| **TXNDC5** | 0.258261515 | 0.01208764 |
| **SPOCK1** | 0.258085859 | 0.035566829 |
| **CYP26B1** | 0.252533488 | 0.014848137 |
| **IL6** | 0.24927113 | 0.015122803 |
| **SERPINE1** | 0.246629676 | 0.015570847 |
| **CDH19** | 0.239147918 | 0.015650666 |
| **STEAP1B** | 0.235552145 | 0.036745395 |
| **LOX** | 0.228956837 | 0.024665784 |
| **STEAP1** | 0.227126261 | 0.020427341 |
| **ENC1** | 0.226284054 | 0.004082047 |
| **NLGN4X** | 0.223336301 | 0.029250501 |
| **KDELR3** | 0.219180883 | 0.040148 |
| **MAPRE2** | 0.212466509 | 0.003625556 |
| **SLC22A17** | 0.21243912 | 0.022122242 |
| **RHOBTB3** | 0.212279483 | 0.018958574 |
| **CPXM2** | 0.208150017 | 0.049299317 |
| **BEX4** | 0.207955896 | 0.018947657 |
| **AKR1B1** | 0.205327352 | 0.010823958 |
| **SNAP25** | 0.202488838 | 0.014831829 |
| **ITGB1** | 0.202169353 | 0.036204412 |
| **STEAP2** | 0.200626565 | 0.010392322 |
| **NAP1L5** | 0.200542025 | 0.003587764 |
| **CTNNB1** | 0.198296261 | 0.028615649 |
| **ETV5** | 0.197830118 | 0.016257222 |
| **OPN3** | 0.19698195 | 0.03061304 |
| **ACTB** | 0.19649432 | 0.024231908 |
| **SOCS3** | 0.196018347 | 0.002495364 |
| **PID1** | 0.195510562 | 0.005575822 |
| **WIF1** | 0.194597238 | 0.008826115 |
| **FGF9** | 0.191001881 | 0.03588381 |
| **BEX5** | 0.190395853 | 0.003742504 |
| **SPANXB2** | 0.190241275 | 0.01887082 |
| **CEMIP2** | 0.189026905 | 0.001882612 |
| **FEZ1** | 0.188629163 | 0.039718629 |
| **CASC4** | 0.185669198 | 0.004932603 |
| **KRT10** | 0.185372224 | 0.009574153 |
| **SMAD6** | 0.185310215 | 0.011500501 |
| **MANSC1** | 0.183889731 | 0.015830727 |
| **P4HA2** | 0.18211003 | 0.029414986 |
| **CHN1** | 0.181581385 | 0.035856648 |
| **GNG7** | 0.178214229 | 0.029898055 |
| **FAM118A** | 0.17617062 | 0.002000561 |
| **KLF9** | 0.175947475 | 0.046302666 |
| **DNER** | 0.174108802 | 0.038740715 |
| **SECISBP2L** | 0.173863136 | 0.001927901 |
| **TMED10** | 0.173180168 | 0.022194384 |
| **MPRIP** | 0.173179773 | 0.002485346 |
| **HNRNPA0** | 0.172808717 | 0.016821398 |
| **VCL** | 0.170505709 | 0.021784284 |
| **GNAQ** | 0.170140312 | 0.005564696 |
| **SNAP23** | 0.168859076 | 0.021537919 |
| **HOXC6** | 0.168712547 | 0.043346671 |
| **PKIA** | 0.16636676 | 0.046952571 |
| **GLRB** | 0.16630319 | 0.013765607 |
| **TCEAL1** | 0.163847959 | 0.019819519 |
| **TENM3** | 0.163605478 | 0.047686592 |
| **ITGA10** | 0.160177486 | 0.04487088 |
| **SPANXA2** | 0.160036386 | 0.020821682 |
| **GLS** | 0.159327382 | 0.033224092 |
| **OSMR** | 0.159321345 | 0.005836156 |
| **AXIN2** | 0.158582672 | 0.049987368 |
| **TMEM131** | 0.157238164 | 0.005308525 |
| **RBPMS2** | 0.156490873 | 0.019554911 |
| **EIF3L** | 0.154910511 | 0.010211246 |
| **NTF3** | 0.153960277 | 0.027282992 |
| **MORF4L2** | 0.153946507 | 0.044469059 |
| **PI4KAP1** | 0.15384728 | 0.003990517 |
| **ARRDC3** | 0.153019249 | 0.005927129 |
| **PCDHB17P** | 0.152955767 | 0.047537841 |
| **DENND5B** | 0.152774657 | 0.008273996 |
| **SHISAL1** | 0.152591655 | 0.030817175 |
| **NPR 2.00** | 0.151136234 | 0.006471595 |
| **FZD1** | 0.150063586 | 0.012779749 |
| **PJA1** | 0.150024468 | 0.012219908 |
| **PITPNC1** | 0.148760802 | 0.017237105 |
| **SLAIN1** | 0.14811746 | 0.028891383 |
| **CANT1** | 0.146000566 | 0.021391479 |
| **CTNNA1** | 0.144306275 | 0.015649036 |
| **ETS2** | 0.144199551 | 0.039871282 |
| **SKAP2** | 0.144193712 | 0.035430726 |
| **SPANXB1** | 0.144034005 | 0.014439464 |
| **STRN3** | 0.143701366 | 0.005817814 |
| **SGSM1** | 0.143239447 | 0.022026893 |
| **WFDC1** | 0.141429866 | 0.039401599 |
| **DYSF** | 0.139828278 | 0.048412448 |
| **SMTN** | 0.139766392 | 0.015906663 |
| **ATP8B2** | 0.13860701 | 0.006022081 |
| **RNF144A** | 0.138571173 | 0.040213872 |
| **MANBA** | 0.138508037 | 0.026613746 |
| **PLPPR4** | 0.134823217 | 0.003999876 |
| **SGSH** | 0.132766925 | 0.03929933 |
| **TMEM203** | 0.132421581 | 0.003599303 |
| **TCEAL3** | 0.132251191 | 0.049678952 |
| **SPTBN1** | 0.132064908 | 0.035042674 |
| **PNMT** | 0.13149979 | 0.025046544 |
| **SEC22C** | 0.131350897 | 0.036502022 |
| **CXCL1** | 0.129899107 | 0.004918214 |
| **GSTM5** | 0.129631248 | 0.048449625 |
| **MDFIC** | 0.128850351 | 0.025758338 |
| **AQP1** | 0.128611739 | 0.049385415 |
| **PELI2** | 0.127180585 | 0.032919262 |
| **WDR45B** | 0.127026503 | 0.035480404 |
| **CRIM1** | 0.126923852 | 0.030618524 |
| **MATR3** | 0.126507141 | 0.041313058 |
| **EMC4** | 0.124727778 | 0.020334611 |
| **ERCC1** | 0.124091072 | 0.040688224 |
| **TBCD** | 0.123633667 | 0.030230066 |
| **CLASP2** | 0.123465159 | 0.004324767 |
| **CSNK2A1** | 0.123307166 | 0.045693495 |
| **RBM4** | 0.122372594 | 0.045518214 |
| **ARMH4** | 0.121682097 | 0.020650612 |
| **STEAP4** | 0.121421437 | 0.046876751 |
| **FBXO3** | 0.120929633 | 0.033966305 |
| **CUX1** | 0.120288344 | 0.035215343 |
| **NT5C1B** | 0.119873364 | 0.022675846 |
| **SRP68** | 0.119181536 | 0.015734298 |
| **DLGAP1** | 0.118795644 | 0.032189026 |
| **XRN2** | 0.118047306 | 0.03284832 |
| **VDAC1** | 0.117914085 | 0.034549761 |
| **MAEA** | 0.11755238 | 0.026506924 |
| **NORAD** | 0.116770447 | 0.033916747 |
| **RHOT1** | 0.116437727 | 0.015184928 |
| **SLC26A11** | 0.115059662 | 0.03924285 |
| **ABCC1** | 0.114428282 | 0.020540079 |
| **PIGK** | 0.114193647 | 0.049114753 |
| **RTCB** | 0.114159322 | 0.00964772 |
| **PRRC2C** | 0.113805301 | 0.02159615 |
| **XYLT2** | 0.113483782 | 0.022537086 |
| **CLINT1** | 0.11311473 | 0.025123807 |
| **PPP2CA** | 0.112668094 | 0.019395427 |
| **NIPA2** | 0.111290483 | 0.019761078 |
| **ZNF106** | 0.11051035 | 0.036470094 |
| **MAP4K4** | 0.109670865 | 0.044265305 |
| **SEC23A** | 0.109342711 | 0.031219979 |
| **UBR1** | 0.108878336 | 0.005357585 |
| **LRPAP1** | 0.108427599 | 0.028624267 |
| **SRSF2** | 0.108394529 | 0.012773208 |
| **NPLOC4** | 0.108143771 | 0.033370297 |
| **PTPRZ1** | 0.107984809 | 0.0463736 |
| **FNIP1** | 0.107039682 | 0.010820423 |
| **TANC1** | 0.105085774 | 0.032649491 |
| **B3GAT1** | 0.105007434 | 0.018639512 |
| **ADAM10** | 0.104065939 | 0.030210774 |
| **ZNF662** | 0.103401069 | 0.025292667 |
| **RFNG** | 0.10313853 | 0.048894567 |
| **PFDN1** | 0.103009105 | 0.011490968 |
| **OGFOD3** | 0.102832182 | 0.049404309 |
| **NUDT6** | 0.102592538 | 0.021852458 |
| **LCMT2** | 0.102290881 | 0.040148053 |
| **SPANXD** | 0.102026128 | 0.032247902 |
| **RNF4** | 0.101893307 | 0.018223586 |
| **ZNRF3** | 0.101554122 | 0.003794643 |
| **CENPO** | 0.101296568 | 0.024318757 |
| **PIGB** | 0.101291446 | 0.020733092 |
| **AAR2** | 0.101267431 | 0.023877355 |
| **SPANXC** | 0.100216346 | 0.046448203 |
| **ZNF548** | 0.099507058 | 0.025632912 |
| **CLEC4G** | 0.098124522 | 0.032386491 |
| **QSOX2** | 0.097169398 | 0.040656426 |
| **PI4KAP2** | 0.09667554 | 0.014422535 |
| **SP2** | 0.095725101 | 0.022132777 |
| **EMC7** | 0.095479882 | 0.044048402 |
| **HAS2** | 0.095344278 | 0.021739781 |
| **DCUN1D3** | 0.095100771 | 0.028317264 |
| **CTBP1** | 0.094417463 | 0.046522369 |
| **AKAP13** | 0.094317396 | 0.049984865 |
| **MAGED4** | 0.093844776 | 0.046457607 |
| **TP53BP1** | 0.09287317 | 0.030966071 |
| **GRM3** | 0.092834679 | 0.020392814 |
| **BRINP2** | 0.091763861 | 0.016316623 |
| **BRD8** | 0.091414262 | 0.021019519 |
| **PIGG** | 0.089675605 | 0.033425188 |
| **THAP4** | 0.089296735 | 0.045136818 |
| **BICRA** | 0.088314387 | 0.025557892 |
| **CTCF** | 0.087526975 | 0.012146861 |
| **TUBGCP4** | 0.086423865 | 0.020082163 |
| **PAX9** | 0.086372536 | 0.046906079 |
| **EIF3J** | 0.085716636 | 0.043235936 |
| **CHMP6** | 0.085654055 | 0.027792546 |
| **HIVEP1** | 0.085383161 | 0.043701578 |
| **ZC3H7A** | 0.084812288 | 0.039875312 |
| **NPPC** | 0.08480266 | 0.02380609 |
| **MSANTD2** | 0.08459416 | 0.037826093 |
| **SCN1B** | 0.084514233 | 0.04322127 |
| **RGN** | 0.083769061 | 0.030848456 |
| **HIC2** | 0.083615147 | 0.042894835 |
| **B4GAT1** | 0.083163278 | 0.046454936 |
| **PLD5** | 0.083008183 | 0.008492874 |
| **MRC2** | 0.079935147 | 0.018760905 |
| **PRR16** | 0.079774838 | 0.042807166 |
| **PTCH1** | 0.079188251 | 0.015632063 |
| **NTRK3** | 0.079167993 | 0.038706281 |
| **TRIM23** | 0.078100718 | 0.022744806 |
| **PROK2** | 0.077934922 | 0.02323126 |
| **MICAL2** | 0.077768535 | 0.028642568 |
| **CARNS1** | 0.077645964 | 0.044829043 |
| **CDPF1** | 0.076824169 | 0.010120111 |
| **PTPRN** | 0.076498386 | 0.034969885 |
| **OPALIN** | 0.07499228 | 0.005814712 |
| **ALKBH1** | 0.074538688 | 0.043975833 |
| **SPTBN5** | 0.07395335 | 0.024598941 |
| **CSNK1G3** | 0.073927725 | 0.041923607 |
| **NR4A1** | 0.073780291 | 0.040375874 |
| **NPFFR2** | 0.07352452 | 0.049397064 |
| **PARG** | 0.073145955 | 0.042570956 |
| **MYO1E** | 0.073063677 | 0.018659681 |
| **EDAR** | 0.072195334 | 0.033014759 |
| **ARC** | 0.071516767 | 0.035865179 |
| **RAD50** | 0.071044882 | 0.04650258 |
| **DTD2** | 0.070728955 | 0.006521599 |
| **AGFG2** | 0.069991301 | 0.005578995 |
| **LINC01119** | 0.06937816 | 0.043619449 |
| **HMGXB4** | 0.069181277 | 0.010217239 |
| **ABCB4** | 0.06911478 | 0.032914824 |
| **LOC285147** | 0.06831009 | 0.009987568 |
| **SLC17A6** | 0.068082414 | 0.033935611 |
| **PIK3C2A** | 0.067653039 | 0.038333235 |
| **SEPTIN8** | 0.067467307 | 0.006903487 |
| **ELP4** | 0.06722394 | 0.049819586 |
| **FRAT1** | 0.067127445 | 0.012170539 |
| **COL9A1** | 0.066347678 | 0.04689312 |
| **EPS8L2** | 0.066013635 | 0.001565432 |
| **IL17RA** | 0.065640948 | 0.046885945 |
| **NELFA** | 0.064791816 | 0.031258126 |
| **UGT3A2** | 0.064738389 | 0.004247846 |
| **RFPL3S** | 0.064603587 | 0.046723658 |
| **GCC2** | 0.064397423 | 0.018029708 |
| **IREB2** | 0.063656858 | 0.026443133 |
| **RBFOX1** | 0.063622816 | 0.000499439 |
| **ELAVL4** | 0.06349627 | 0.044658226 |
| **C5orf24** | 0.063035674 | 0.034770521 |
| **NOTUM** | 0.062664767 | 0.004564617 |
| **GPR176** | 0.062542367 | 0.012509322 |
| **CHL1** | 0.062203941 | 0.034276166 |
| **FPR2** | 0.061211317 | 0.017723319 |
| **HHATL** | 0.061133476 | 0.035260465 |
| **UBXN7** | 0.059561948 | 0.028178001 |
| **ERO1B** | 0.057974824 | 0.048478127 |
| **SLC22A14** | 0.05780659 | 0.006325979 |
| **ZXDB** | 0.05751306 | 0.026632336 |
| **PDYN** | 0.057128394 | 0.020470405 |
| **MIA2** | 0.056492894 | 0.035133171 |
| **KRTAP5-9** | 0.056400894 | 0.002494147 |
| **ELOVL2** | 0.055452819 | 0.036806583 |
| **CT45A1** | 0.054935851 | 0.030890184 |
| **PCDHA3** | 0.054167825 | 0.005151309 |
| **DGKG** | 0.053975565 | 0.01739619 |
| **CACUL1** | 0.053184821 | 0.030484203 |
| **HS3ST4** | 0.051486956 | 0.004633392 |
| **ARHGEF28** | 0.05130566 | 0.018460225 |
| **HIPK3** | 0.051188566 | 0.040551192 |
| **LINC00052** | 0.050941445 | 0.015258807 |
| **ADAMTS12** | 0.050369814 | 0.000829606 |
| **LOC643201** | 0.050269762 | 0.000405332 |
| **OPN1SW** | 0.049837631 | 0.01089789 |
| **DKFZp451A211** | 0.049674813 | 0.042368493 |
| **LRRTM1** | 0.049625529 | 0.013956238 |
| **ETV1** | 0.049094431 | 0.003011591 |
| **ROS1** | 0.048711292 | 0.049378359 |
| **TCAF2** | 0.048317601 | 0.025070204 |
| **MSTN** | 0.047707511 | 0.035939672 |
| **PLEKHG7** | 0.047459456 | 0.027893431 |
| **CFL2** | 0.047413227 | 0.049848126 |
| **KRTAP10-11** | 0.047365265 | 0.019981655 |
| **TULP2** | 0.047247724 | 0.031983956 |
| **MIER3** | 0.047179727 | 0.012064371 |
| **IL17RE** | 0.047152844 | 0.035605087 |
| **RETN** | 0.047041266 | 0.012436967 |
| **DNAJB3** | 0.04584403 | 0.035647345 |
| **FLJ22447** | 0.04553997 | 0.041383641 |
| **TIGD4** | 0.044742959 | 0.001865696 |
| **AK7** | 0.04459294 | 0.003163145 |
| **KAT7** | 0.04441951 | 0.0362007 |
| **C11orf44** | 0.04439116 | 0.005871944 |
| **AKAP14** | 0.044382471 | 0.018500968 |
| **APOA1** | 0.043734593 | 0.032917314 |
| **REG4** | 0.042891711 | 0.006444791 |
| **C15orf21** | 0.04268886 | 0.008371862 |
| **ARHGEF26** | 0.042419962 | 0.012867416 |
| **ADH6** | 0.042280141 | 0.005438083 |
| **DUSP7** | 0.041784855 | 0.008307157 |
| **LIM2** | 0.041525665 | 0.010877732 |
| **C9orf174** | 0.041341921 | 0.00434124 |
| **TAF8** | 0.040533163 | 0.04864604 |
| **OR8K3** | 0.04050756 | 0.024505835 |
| **ZNF80** | 0.040136247 | 0.040786112 |
| **KRT74** | 0.039960079 | 0.042398253 |
| **OTOP1** | 0.039778429 | 0.031618818 |
| **LINC00628** | 0.039706461 | 0.029282912 |
| **FAM66C** | 0.039668362 | 0.019145803 |
| **CNGA4** | 0.039419552 | 0.018124724 |
| **STX1B** | 0.039399949 | 0.00618548 |
| **ADAM20** | 0.039386171 | 0.012763406 |
| **MIXL1** | 0.039069495 | 0.03646182 |
| **TAS2R46** | 0.039050712 | 0.013244728 |
| **ZNF157** | 0.03896968 | 0.013701966 |
| **NEK4** | 0.038694658 | 0.046447061 |
| **CDKN2A-DT** | 0.038657833 | 0.016569428 |
| **WAKMAR2** | 0.038623126 | 0.017787092 |
| **LOC441666** | 0.038411213 | 0.013168041 |
| **CCDC96** | 0.037857422 | 0.026755076 |
| **RPL13AP17** | 0.037355733 | 0.016729868 |
| **EME2** | 0.037346618 | 0.017478869 |
| **DDX53** | 0.037249984 | 0.018194462 |
| **KIF5A** | 0.037153139 | 0.049843723 |
| **KMT5C** | 0.037026703 | 0.031496114 |
| **C16orf97** | 0.037004795 | 0.015658866 |
| **OR4L1** | 0.036979624 | 0.039972593 |
| **XKR5** | 0.036945554 | 0.035859193 |
| **KHDRBS2** | 0.036501236 | 0.014252441 |
| **FLJ31945** | 0.036212227 | 0.046958197 |
| **C11orf97** | 0.035913068 | 0.035657289 |
| **CCDC38** | 0.035908715 | 0.025800198 |
| **IL5** | 0.035649326 | 0.02651172 |
| **TRPV3** | 0.035555296 | 0.040650001 |
| **AK096549** | 0.03547417 | 0.01177298 |
| **GALR3** | 0.035355799 | 0.032944626 |
| **RAB6D** | 0.035264804 | 0.022999186 |
| **OR4N4** | 0.035234758 | 0.039575851 |
| **ZNF187** | 0.035000037 | 0.025362175 |
| **SOX3** | 0.034991755 | 0.047515472 |
| **LRIT1** | 0.034508805 | 0.03104091 |
| **C5orf49** | 0.034481977 | 0.028743821 |
| **SH3TC2-DT** | 0.034459223 | 0.020844162 |
| **LINC00160** | 0.034347843 | 0.034236658 |
| **MCIDAS** | 0.034244053 | 0.014263902 |
| **LINC02740** | 0.034207594 | 0.023848215 |
| **SPP2** | 0.034155177 | 0.047889261 |
| **FAM197Y2** | 0.034067691 | 0.029859146 |
| **LRRD1** | 0.03403591 | 0.025075912 |
| **PAX4** | 0.03395198 | 0.022555462 |
| **LHX2-AS1** | 0.033917079 | 0.01597866 |
| **SYN3** | 0.033851338 | 0.027213064 |
| **FAM38B** | 0.033709671 | 0.031842693 |
| **PMCHL1** | 0.033391052 | 0.034514878 |
| **FAM171A2** | 0.033367341 | 0.025331999 |
| **OR4S2** | 0.033033026 | 0.034440145 |
| **TRPA1** | 0.032849686 | 0.025755117 |
| **OR8B8** | 0.032165035 | 0.034891212 |
| **ATOH7** | 0.032108954 | 0.048526764 |
| **TMEM72** | 0.031895056 | 0.024053329 |
| **OR6Q1** | 0.031887168 | 0.027464743 |
| **TCEB3C** | 0.031793782 | 0.043030615 |
| **PCDHGC4** | 0.031719475 | 0.038836931 |
| **TANGO6** | 0.031423074 | 0.044464095 |
| **BC048428** | 0.030597962 | 0.048427719 |
| **H2BC10** | 0.030408086 | 0.037974273 |
| **TMEM14EP** | 0.03035702 | 0.03439523 |
| **LOC100507271** | 0.030112457 | 0.037720616 |
| **NOXRED1** | 0.030027656 | 0.049773534 |
| **RBMXL3** | 0.02984652 | 0.038223973 |
| **FAM71E2** | 0.02979838 | 0.046976404 |
| **LOC154449** | 0.029744877 | 0.047260829 |
| **NEU2** | 0.029386305 | 0.03594126 |
| **OR2T3** | 0.028986658 | 0.041240085 |
| **GABRQ** | 0.027965787 | 0.04506779 |
| **OR4D9** | -0.026605666 | 0.049133196 |
| **H2BW4P** | -0.027936862 | 0.039252353 |
| **FAM63A** | -0.028466829 | 0.036437686 |
| **FBXO39** | -0.02888635 | 0.037073231 |
| **LOC392232** | -0.029247341 | 0.021721528 |
| **PRAMEF20** | -0.029502978 | 0.016836301 |
| **ESRRB** | -0.029645558 | 0.044705337 |
| **KRTAP2-1** | -0.029723959 | 0.048886182 |
| **DEFB136** | -0.029949229 | 0.035867866 |
| **FFAR1** | -0.030076598 | 0.031780362 |
| **OR2Z1** | -0.030622481 | 0.036684141 |
| **CT45A2** | -0.031050146 | 0.028859208 |
| **C14orf144** | -0.031104054 | 0.038556688 |
| **PDE7B-AS1** | -0.031609606 | 0.041615211 |
| **STATH** | -0.031907718 | 0.045736894 |
| **KRT82** | -0.03212253 | 0.04948563 |
| **GDF3** | -0.032128358 | 0.03081086 |
| **PRRT4** | -0.032174748 | 0.018308148 |
| **USP28** | -0.032349276 | 0.046934529 |
| **ZNF117** | -0.032457664 | 0.031159556 |
| **WFDC11** | -0.032538144 | 0.042422168 |
| **SCN10A** | -0.032578085 | 0.020416805 |
| **BX648939** | -0.032666692 | 0.040636284 |
| **C10orf120** | -0.033007346 | 0.027230856 |
| **UOX** | -0.033193047 | 0.028597668 |
| **IRGQ** | -0.033347461 | 0.025595725 |
| **CPO** | -0.033350218 | 0.024400871 |
| **PPCDC** | -0.033427338 | 0.047825532 |
| **HIGD2B** | -0.033432762 | 0.012372946 |
| **MICU3** | -0.033568243 | 0.024416777 |
| **CFHR3** | -0.03363604 | 0.042522679 |
| **TNP2** | -0.033880176 | 0.018064228 |
| **FOXR2** | -0.033989749 | 0.039143983 |
| **CLDN22** | -0.034179412 | 0.034980505 |
| **STPG1** | -0.034250002 | 0.045508385 |
| **LOC100131581** | -0.034641939 | 0.030623547 |
| **BAIAP3** | -0.034787248 | 0.045035777 |
| **GRIN3A** | -0.035116914 | 0.032203872 |
| **HDX** | -0.035503902 | 0.045367965 |
| **C21orf136** | -0.035558278 | 0.03591504 |
| **LOC284009** | -0.035656345 | 0.021355844 |
| **METTL8** | -0.035714544 | 0.025367213 |
| **C9orf47** | -0.035816784 | 0.049861415 |
| **WBP11P1** | -0.036089544 | 0.01667585 |
| **OR5H14** | -0.036292232 | 0.019939615 |
| **GP9** | -0.036419026 | 0.012124019 |
| **HES7** | -0.036795827 | 0.038302691 |
| **FTMT** | -0.036848196 | 0.035074631 |
| **GPN3** | -0.037222743 | 0.033053485 |
| **OR6F1** | -0.037418531 | 0.017664825 |
| **OR13C3** | -0.037485222 | 0.015417274 |
| **OR52H1** | -0.037862594 | 0.025257897 |
| **MRGPRG-AS1** | -0.037932125 | 0.004588767 |
| **IL31** | -0.037951742 | 0.02813963 |
| **LPAAT-delta** | -0.038120116 | 0.011049954 |
| **LRRC4** | -0.038406523 | 0.026945043 |
| **SNORD42A** | -0.038649175 | 0.00968102 |
| **ADAM33** | -0.039031116 | 0.04767965 |
| **TBX4** | -0.039067131 | 0.014785751 |
| **LINC01100** | -0.039208482 | 0.007337726 |
| **OR10K2** | -0.039418875 | 0.016542322 |
| **GNG8** | -0.039622809 | 0.047519386 |
| **EML5** | -0.039686927 | 0.007698365 |
| **HTR3D** | -0.039736526 | 0.007562057 |
| **HCAR1** | -0.039811434 | 0.0355116 |
| **PGR** | -0.039847381 | 0.020078291 |
| **FAM21EP** | -0.040108084 | 0.007006334 |
| **PHF21B** | -0.040432789 | 0.014961013 |
| **RBMY1A1** | -0.040448597 | 0.023833333 |
| **TTLL10** | -0.040653104 | 0.03153416 |
| **GRIK3** | -0.040728574 | 0.022904931 |
| **PRM3** | -0.040788717 | 0.006792914 |
| **STH** | -0.041246036 | 0.037031555 |
| **MYMK** | -0.041551949 | 0.014516738 |
| **ZNF90** | -0.042069553 | 0.006056169 |
| **SPEM1** | -0.042212871 | 0.00870463 |
| **CAMTA2** | -0.042262421 | 0.04841756 |
| **LINC01589** | -0.042320788 | 0.018084865 |
| **PEX12** | -0.04242729 | 0.009250727 |
| **LOC441426** | -0.042430278 | 0.037642049 |
| **TGFBR3L** | -0.04276659 | 0.023383064 |
| **C1orf211** | -0.043366276 | 0.021435664 |
| **LINC00841** | -0.043439392 | 0.002726351 |
| **CCDC177** | -0.043560181 | 0.008065578 |
| **EEF1AKMT2** | -0.043716908 | 0.043172849 |
| **SERPINC1** | -0.044316009 | 0.011047144 |
| **AMZ1** | -0.044331924 | 0.004124926 |
| **H4C4** | -0.044428192 | 0.011379689 |
| **CALML6** | -0.04459212 | 0.01650208 |
| **PCYOX1L** | -0.044632813 | 0.035447758 |
| **SPATA31D1** | -0.044675009 | 0.007068726 |
| **NBPF22P** | -0.045892818 | 0.041128208 |
| **TRPV5** | -0.046001615 | 0.014863503 |
| **EDDM3A** | -0.046009019 | 0.015702732 |
| **XPA** | -0.046171877 | 0.019425412 |
| **MAS1** | -0.046885354 | 0.02143635 |
| **CACNA1G-AS1** | -0.046966056 | 0.036921983 |
| **GLI1** | -0.04703638 | 0.032871151 |
| **PRSS40B** | -0.04733988 | 0.026332048 |
| **TFAP2E** | -0.047848167 | 0.034511832 |
| **MTG2** | -0.048127167 | 0.01636986 |
| **SERPINI2** | -0.048227344 | 0.001672737 |
| **C1orf146** | -0.048362741 | 0.001553619 |
| **TNK1** | -0.048763599 | 0.015504649 |
| **C17orf98** | -0.04903518 | 0.038756219 |
| **TMEM65** | -0.049667839 | 0.005530639 |
| **KAT5** | -0.05034807 | 0.04163469 |
| **LINC00346** | -0.050716316 | 0.041819343 |
| **TAB2** | -0.051267129 | 0.042983386 |
| **TIGD6** | -0.051442536 | 0.003030753 |
| **HOXB1** | -0.051809308 | 0.009714415 |
| **FGF19** | -0.05286936 | 0.008273072 |
| **C2orf69** | -0.054642586 | 0.021057026 |
| **ZNF420** | -0.054885832 | 0.009916534 |
| **AGRP** | -0.055190141 | 0.037406274 |
| **CCAR1** | -0.055483948 | 0.041642743 |
| **GBP3** | -0.055744646 | 0.031749987 |
| **ZBTB1** | -0.055919 | 0.001065837 |
| **DMP1** | -0.056021134 | 0.001763825 |
| **LINC01602** | -0.056068537 | 0.017524967 |
| **E2F1** | -0.056178435 | 0.005071846 |
| **FAM76A** | -0.056255222 | 0.029604129 |
| **TMA16** | -0.056847387 | 0.045324583 |
| **SPIC** | -0.057413336 | 0.025150423 |
| **APOBEC3A** | -0.057452654 | 0.01500084 |
| **CCDC153** | -0.057675106 | 0.003276579 |
| **MMAA** | -0.057889162 | 0.032502523 |
| **CD80** | -0.059022706 | 0.038548024 |
| **ADH4** | -0.05944113 | 0.025074033 |
| **NR2E1** | -0.060257628 | 0.025308852 |
| **ZNF813** | -0.060608903 | 0.005289025 |
| **SESN2** | -0.060835754 | 0.015272991 |
| **SEMG1** | -0.0610173 | 0.028013906 |
| **USP41** | -0.061277241 | 0.011724151 |
| **ORAI3** | -0.061648221 | 0.014432463 |
| **FOXI2** | -0.062304293 | 0.017475451 |
| **KY** | -0.062392574 | 0.039881277 |
| **C11orf58** | -0.062560999 | 0.043733594 |
| **TMEM171** | -0.062973491 | 0.03822736 |
| **TIMMDC1** | -0.063017161 | 0.043010429 |
| **GTPBP2** | -0.064450283 | 0.020709262 |
| **DDX20** | -0.064763696 | 0.005459818 |
| **CCL1** | -0.066042217 | 0.042607802 |
| **ZNF79** | -0.066194734 | 0.049183825 |
| **CREM** | -0.06657635 | 0.008857822 |
| **HCFC2** | -0.066655604 | 0.036329886 |
| **ZNF383** | -0.067220445 | 0.045884552 |
| **PXN** | -0.067570464 | 0.04676885 |
| **MUC21** | -0.067570945 | 0.044731965 |
| **CCR5** | -0.069136073 | 0.000657038 |
| **NAA35** | -0.069684583 | 0.046558698 |
| **DCAF17** | -0.070235253 | 0.016126745 |
| **HSPA13** | -0.070381608 | 0.04814107 |
| **PRR22** | -0.070737388 | 0.048379131 |
| **PMS1** | -0.071356768 | 0.03511782 |
| **OSBPL11** | -0.071644979 | 0.043065194 |
| **SFI1** | -0.071885391 | 0.027246059 |
| **RAD1** | -0.072107293 | 0.045300787 |
| **ESCO1** | -0.072640809 | 0.040449208 |
| **UBE3B** | -0.07385021 | 0.038959748 |
| **NKX2-3** | -0.075077858 | 0.023584778 |
| **TCP10L** | -0.076090988 | 0.041990457 |
| **MUS81** | -0.076120458 | 0.034613372 |
| **HESX1** | -0.076317077 | 0.04663609 |
| **ZNF75A** | -0.077417774 | 0.024618289 |
| **CUL4B** | -0.077784674 | 0.045636184 |
| **IFIT5** | -0.077957516 | 0.025804474 |
| **KIAA0100** | -0.078025145 | 0.008696406 |
| **VAPB** | -0.078336884 | 0.037712876 |
| **IFNL1** | -0.078655103 | 0.019645142 |
| **MED21** | -0.078894723 | 0.01651863 |
| **CENPQ** | -0.079309408 | 0.033662483 |
| **RTF2** | -0.079476867 | 0.030176273 |
| **SNORD15B** | -0.080381605 | 0.039428385 |
| **WEE2-AS1** | -0.08054855 | 0.006569229 |
| **ZFP69B** | -0.080925366 | 0.040305776 |
| **ZBTB11** | -0.081392842 | 0.004537097 |
| **GSTA5** | -0.082140254 | 0.012948764 |
| **CAAP1** | -0.082616953 | 0.0230203 |
| **C9orf64** | -0.08305797 | 0.036410293 |
| **WDR81** | -0.083497939 | 0.027684207 |
| **PIK3R3** | -0.08378202 | 0.014951701 |
| **TMEM50A** | -0.083837563 | 0.016209038 |
| **COG3** | -0.084410275 | 0.042026725 |
| **MEIOB** | -0.085486954 | 0.042005847 |
| **RDM1** | -0.08590451 | 0.031304651 |
| **TMEM198B** | -0.08595888 | 0.030287178 |
| **FAS** | -0.086341303 | 0.029223267 |
| **SNIP1** | -0.086533229 | 0.012362591 |
| **TAF4B** | -0.086853439 | 0.030239862 |
| **TAF1D** | -0.086866282 | 0.012253382 |
| **HYI** | -0.087262173 | 0.045836296 |
| **FMO9P** | -0.087763699 | 0.015267662 |
| **TLK1** | -0.087905416 | 0.036334655 |
| **UNC13B** | -0.088808042 | 0.028725999 |
| **ARG2** | -0.089594982 | 0.00757897 |
| **ANXA9** | -0.09113611 | 0.03914503 |
| **ZNF140** | -0.091399879 | 0.017704335 |
| **PSENEN** | -0.092028974 | 0.029140128 |
| **SLF1** | -0.092216592 | 0.002325875 |
| **TMEM31** | -0.092689452 | 0.010833995 |
| **PCNX3** | -0.093033969 | 0.023932357 |
| **ERMAP** | -0.093036297 | 0.033841295 |
| **NAA38** | -0.09376137 | 0.035294413 |
| **LIPT2-AS1** | -0.093822899 | 0.032190734 |
| **MANEAL** | -0.095689518 | 0.029957416 |
| **DIP2A** | -0.095782149 | 0.029392537 |
| **ZCWPW1** | -0.095916446 | 0.037650554 |
| **ZNF263** | -0.096013153 | 0.007616007 |
| **PHF23** | -0.096386916 | 0.014134639 |
| **GRHPR** | -0.096736232 | 0.027611684 |
| **TMEM219** | -0.097215047 | 0.040428753 |
| **ERI3** | -0.09803788 | 0.042896762 |
| **NME2** | -0.098119133 | 0.040464078 |
| **RNF167** | -0.098623189 | 0.029464168 |
| **CSTA** | -0.099793636 | 0.02503868 |
| **GPBP1L1** | -0.100540228 | 0.013048677 |
| **CARS2** | -0.100613073 | 0.039263504 |
| **EIF3C** | -0.10064736 | 0.040686351 |
| **SS18L1** | -0.100978232 | 0.041934053 |
| **RNF114** | -0.101187469 | 0.044556328 |
| **TAF13** | -0.101296114 | 0.04886237 |
| **ETV7** | -0.101422088 | 0.036186779 |
| **LOC650293** | -0.102193315 | 0.040637022 |
| **CD164L2** | -0.102780455 | 0.045770274 |
| **RNASEH2C** | -0.102966681 | 0.042430501 |
| **NDUFS2** | -0.103095398 | 0.031553831 |
| **TELO2** | -0.103173289 | 0.029256297 |
| **IFT172** | -0.103426456 | 0.037571614 |
| **HNRNPLL** | -0.103446613 | 0.045292215 |
| **MSH2** | -0.103932491 | 0.009489636 |
| **KCNJ6** | -0.104275476 | 0.035842428 |
| **SPATA2L** | -0.1043616 | 0.019481354 |
| **ZNHIT2** | -0.104385017 | 0.02326768 |
| **PCMT1** | -0.10500443 | 0.047438937 |
| **CNTD2** | -0.105209561 | 0.026108563 |
| **ZIC3** | -0.105244081 | 0.01318612 |
| **NUDT15** | -0.105392697 | 0.03230735 |
| **MAPKAPK2** | -0.106724874 | 0.018496549 |
| **DCAF13** | -0.106779662 | 0.029769545 |
| **STK19** | -0.106844113 | 0.033482644 |
| **ATG5** | -0.1072073 | 0.034766989 |
| **FAM27E3** | -0.108109077 | 0.018403375 |
| **MTF1** | -0.108970532 | 0.035438108 |
| **MRPL47** | -0.109067181 | 0.034600383 |
| **SPAG1** | -0.109531778 | 0.030500598 |
| **AARS1** | -0.109829374 | 0.048148541 |
| **TRAPPC3** | -0.110113797 | 0.020293308 |
| **APPL1** | -0.110174428 | 0.006128565 |
| **POMP** | -0.110252788 | 0.023082742 |
| **TIMM8B** | -0.110331249 | 0.010840227 |
| **VPS26A** | -0.111187962 | 0.049535487 |
| **SLC35C2** | -0.111743512 | 0.023654025 |
| **MANBAL** | -0.111772031 | 0.035099463 |
| **C12orf65** | -0.112083792 | 0.003538092 |
| **PKD1L2** | -0.112448455 | 0.017651598 |
| **HINT2** | -0.112830451 | 0.03792678 |
| **RMI1** | -0.112900898 | 0.046382193 |
| **NHLRC3** | -0.113271293 | 0.025184406 |
| **TSR3** | -0.114214657 | 0.005304031 |
| **TMEM116** | -0.114347934 | 0.020116362 |
| **DPP9** | -0.114933106 | 0.046063329 |
| **ZBTB8A** | -0.114976113 | 0.014877845 |
| **SNX14** | -0.115035349 | 0.029409533 |
| **SSBP1** | -0.115488695 | 0.049989982 |
| **SLC30A8** | -0.115552478 | 0.005857023 |
| **CHFR** | -0.115781927 | 0.018817652 |
| **MLF 1.00** | -0.115825813 | 0.034997656 |
| **MUTYH** | -0.115927719 | 0.028691538 |
| **TTC39B** | -0.116717758 | 0.042181701 |
| **PPDPF** | -0.117085691 | 0.028520267 |
| **NUP43** | -0.118469411 | 0.027896504 |
| **ARMC1** | -0.118817631 | 0.036743011 |
| **XPO4** | -0.119616116 | 0.036437408 |
| **GID8** | -0.119764381 | 0.037369466 |
| **PKN2** | -0.119838679 | 0.043149724 |
| **MGMT** | -0.119847003 | 0.043560616 |
| **SOD1** | -0.120376857 | 0.027375986 |
| **ENO3** | -0.121836467 | 0.038971911 |
| **PUS3** | -0.121900216 | 0.009954741 |
| **FAM72D** | -0.122116517 | 0.027407183 |
| **DRAP1** | -0.122146712 | 0.011380044 |
| **EED** | -0.122170968 | 0.008551739 |
| **PUS1** | -0.122214359 | 0.040629183 |
| **PIN4** | -0.122553784 | 0.035900584 |
| **EXOSC8** | -0.122643522 | 0.019791956 |
| **CALHM6** | -0.123080984 | 0.034274 |
| **DCAF12** | -0.123658526 | 0.024424122 |
| **ESR1** | -0.124081991 | 0.04913569 |
| **ADPRHL2** | -0.124783627 | 0.009968295 |
| **RPP25L** | -0.125594046 | 0.018902811 |
| **VRK2** | -0.125645003 | 0.035868711 |
| **VAMP8** | -0.125693557 | 0.036051922 |
| **TRIT1** | -0.125913405 | 0.021390865 |
| **ACYP2** | -0.126405732 | 0.04754051 |
| **PARPBP** | -0.127160002 | 0.027938928 |
| **EXOSC3** | -0.127706493 | 0.036117547 |
| **CISD2** | -0.127721971 | 0.009934181 |
| **RPL26L1** | -0.127744665 | 0.02011118 |
| **TRPM2** | -0.128504412 | 0.016771961 |
| **DDX27** | -0.128723861 | 0.026441213 |
| **LOC389641** | -0.129105444 | 0.017474884 |
| **SNX16** | -0.129139078 | 0.024099351 |
| **PPIL3** | -0.129937064 | 0.006086647 |
| **ASPHD1** | -0.130370012 | 0.034568158 |
| **TXNDC17** | -0.13069642 | 0.02501916 |
| **HAGHL** | -0.130901895 | 0.042172692 |
| **SV2B** | -0.130910668 | 0.033785153 |
| **ALG5** | -0.132088096 | 0.032761038 |
| **NUDT2** | -0.133354845 | 0.026309658 |
| **CKAP2** | -0.133483386 | 0.022342117 |
| **PEBP1** | -0.134161261 | 0.029871877 |
| **OTOF** | -0.135005705 | 0.023004426 |
| **TDRD7** | -0.135217641 | 0.015423586 |
| **AURKAIP1** | -0.136852177 | 0.013762617 |
| **RNASEH2B** | -0.138173665 | 0.034718 |
| **TAF6** | -0.13876855 | 0.02182645 |
| **PGAM5** | -0.139149331 | 0.031302755 |
| **ZCCHC7** | -0.139411504 | 0.008948936 |
| **SS18** | -0.139918798 | 0.039050515 |
| **BRWD1** | -0.140012931 | 0.019747438 |
| **THOC2** | -0.140269568 | 0.035527825 |
| **DHX58** | -0.140540218 | 0.027707838 |
| **MAPKAPK5** | -0.142805465 | 0.006311053 |
| **C21orf91** | -0.143195066 | 0.001301604 |
| **RPUSD1** | -0.144339296 | 0.006993306 |
| **CHST9** | -0.145389482 | 0.048827638 |
| **RHBDL1** | -0.14635146 | 0.017613841 |
| **ZC2HC1C** | -0.146476921 | 0.01509238 |
| **DENND4C** | -0.146786425 | 0.032736738 |
| **HENMT1** | -0.1475398 | 0.037343001 |
| **TMEM51** | -0.147838804 | 0.033971352 |
| **AGL** | -0.148164569 | 0.037829034 |
| **MTF2** | -0.148503768 | 0.022753279 |
| **TRIM46** | -0.148782091 | 0.032119354 |
| **RRP7A** | -0.148832862 | 0.04514266 |
| **CHODL** | -0.149176248 | 0.008517468 |
| **GLRX2** | -0.149440631 | 0.032253409 |
| **GNL2** | -0.15024401 | 0.010385555 |
| **HECA** | -0.152061931 | 0.009620537 |
| **ZDHHC14** | -0.152391583 | 0.01428657 |
| **NMI** | -0.152702644 | 0.048307316 |
| **IRF2BPL** | -0.152928183 | 0.045667879 |
| **ERGIC2** | -0.153253223 | 0.045926557 |
| **NFX1** | -0.153884814 | 0.003503368 |
| **SHFL** | -0.153977191 | 0.020786886 |
| **FANCG** | -0.154536686 | 0.021679391 |
| **ST6GALNAC4** | -0.154914545 | 0.01649523 |
| **CDA** | -0.155427511 | 0.041954959 |
| **ZNF232** | -0.15710231 | 0.013635503 |
| **PNPO** | -0.15744412 | 0.035976696 |
| **IFT74** | -0.16033298 | 0.0096251 |
| **BHLHE41** | -0.162646967 | 0.028020535 |
| **EPHX3** | -0.163873536 | 0.048121107 |
| **CPB1** | -0.165362843 | 0.04761558 |
| **TM4SF4** | -0.166076734 | 0.031706994 |
| **PCK2** | -0.166494214 | 0.023514728 |
| **ZMYND15** | -0.167223638 | 0.020032392 |
| **ZBTB8OS** | -0.16739782 | 0.009293998 |
| **AK2** | -0.167814649 | 0.013845297 |
| **DDX58** | -0.169981054 | 0.010561936 |
| **CYP4V2** | -0.170417476 | 0.011833269 |
| **YBX2** | -0.17132996 | 0.046258114 |
| **KIAA1522** | -0.172420112 | 0.043073413 |
| **WSB2** | -0.173170994 | 0.008755251 |
| **LARP4** | -0.174491444 | 0.012556465 |
| **EIF2AK2** | -0.174685781 | 0.027094689 |
| **EPHX2** | -0.1752291 | 0.008726692 |
| **PSTPIP2** | -0.177506872 | 0.019786743 |
| **SIGMAR1** | -0.178212355 | 0.011422053 |
| **TOMM5** | -0.178936929 | 0.023162268 |
| **APTX** | -0.180793686 | 0.001724065 |
| **ATP5IF1** | -0.181549054 | 0.001535085 |
| **IFI35** | -0.184019342 | 0.036812423 |
| **PARP12** | -0.18442301 | 0.020748208 |
| **STOML2** | -0.184690825 | 0.007782088 |
| **FAM183A** | -0.185640532 | 0.048404062 |
| **CABYR** | -0.186135248 | 0.045272904 |
| **RTP4** | -0.189506867 | 0.036011788 |
| **NUPR1** | -0.190689541 | 0.023830973 |
| **ABRACL** | -0.191084079 | 0.006973053 |
| **POLR1E** | -0.191138721 | 0.015002432 |
| **HERC6** | -0.198019589 | 0.043941753 |
| **OPTN** | -0.199703097 | 0.004212882 |
| **PARP10** | -0.201722617 | 0.015876642 |
| **RPS21** | -0.203416793 | 0.005182053 |
| **AFDN** | -0.208166344 | 0.038491892 |
| **DENND2D** | -0.209417163 | 0.010849656 |
| **ABCB9** | -0.215551014 | 0.024018043 |
| **SAMD9** | -0.217676564 | 0.013543075 |
| **UQCRH** | -0.218858163 | 0.029679577 |
| **HELZ2** | -0.220497653 | 0.012481914 |
| **OAS3** | -0.222196787 | 0.016296873 |
| **PYCARD** | -0.225290408 | 0.028381453 |
| **EPSTI1** | -0.227601727 | 0.042136676 |
| **FMO2** | -0.228599248 | 0.039738959 |
| **KLHL35** | -0.236168947 | 0.009524815 |
| **MAFA** | -0.23630402 | 0.007911381 |
| **GBP1** | -0.236323139 | 0.044919614 |
| **RSAD2** | -0.243521951 | 0.012876775 |
| **STC2** | -0.244342182 | 0.034798226 |
| **XAF1** | -0.251398853 | 0.01815343 |
| **TCEA3** | -0.253222867 | 0.030512085 |
| **BBOX1** | -0.261439011 | 0.035805442 |
| **IFIH1** | -0.265485461 | 0.008534332 |
| **IFIT3** | -0.268579644 | 0.014370926 |
| **IFI44** | -0.268895203 | 0.013680546 |
| **LAMP3** | -0.270900359 | 0.04001692 |
| **HSD17B1** | -0.279799687 | 0.006392045 |
| **IFIT2** | -0.284304408 | 0.010547112 |
| **OASL** | -0.287157915 | 0.008143989 |
| **OAS1** | -0.287311791 | 0.003606596 |
| **RHEX** | -0.288848577 | 0.038776867 |
| **IFI44L** | -0.290586898 | 0.037464424 |
| **HERC5** | -0.293214605 | 0.009177109 |
| **OAS2** | -0.304720582 | 0.015972017 |
| **BST2** | -0.306953805 | 0.021529102 |
| **MX1** | -0.308975797 | 0.025894165 |
| **CHI3L2** | -0.355114442 | 0.041153915 |
| **GSTA1** | -0.365413246 | 0.020978292 |
| **IFIT1** | -0.365514975 | 0.007539904 |
| **ISG15** | -0.393676229 | 0.005327655 |
| **OLFM4** | -0.428716951 | 0.046533144 |
| **IFI27** | -0.431296498 | 0.001454389 |
